# Energy transfer (EnT) photocatalysis enabled by gold-N-heterocyclic carbene (NHC) complexes†

**DOI:** 10.1039/d2sc00864e

**Published:** 2022-05-18

**Authors:** Ekaterina A. Martynova, Vladislav A. Voloshkin, Sébastien G. Guillet, Francis Bru, Marek Beliš, Kristof Van Hecke, Catherine S. J. Cazin, Steven P. Nolan

**Affiliations:** Department of Chemistry, Centre for Sustainable Chemistry, Ghent University Krijgslaan 281, S3 9000 Ghent Belgium steven.nolan@ugent.be

## Abstract

We present the use of gold sensitizers [Au(SIPr)(Cbz)] (PhotAu 1) and [Au(IPr)(Cbz)] (PhotAu 2) as attractive alternatives to state-of-the-art iridium-based systems. These novel photocatalysts are deployed in [2 + 2] cycloadditions of diallyl ethers and *N*-tosylamides. The reactions proceed in short reaction times and in environmentally friendly solvents. [Au(SIPr)Cbz] and [Au(IPr)(Cbz)] have higher triplet energy (*E*_T_) values (66.6 and 66.3 kcal mol^−1^, respectively) compared to commonly used iridium photosensitizers. These *E*_T_ values permit the use of these gold complexes as sensitizers enabling energy transfer catalysis involving unprotected indole derivatives, a substrate class previously inaccessible with state-of-the-art Ir photocatalysts. The photosynthesis of unprotected tetracyclic spiroindolines *via* intramolecular [2 + 2] cycloaddition using our simple mononuclear gold sensitizer is readily achieved. Mechanistic studies support the involvement of triplet–triplet energy transfer (TTEnT) for both [2 + 2] photocycloadditions.

## Abstract 2

We present the use of gold sensitizers [Au(SIPr)(Cbz)] (PhotAu 1) and [Au(IPr)(Cbz)] (PhotAu 2) as attractive alternatives to state-of-the-art iridium-based systems.

## Graphical abstract

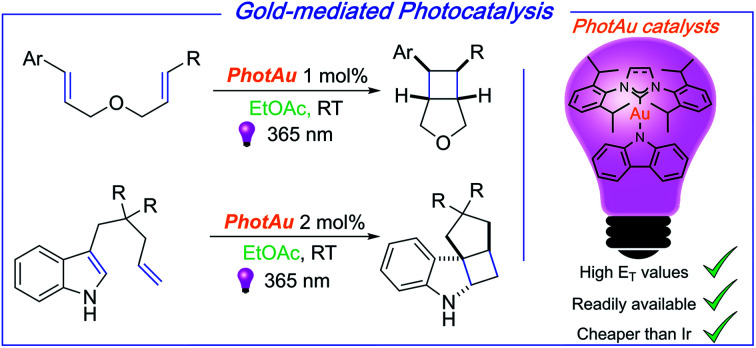

## Introduction

Over the last two decades, the field of photocatalysis has significantly evolved and is nowadays considered a powerful tool in the chemist's synthetic arsenal.^1–5^ While photoredox catalysis has received much attention and has grown into a mature area,^6–10^ energy transfer (EnT) catalysis still remains comparatively underexplored but the area is rapidly evolving.^11–14^ The EnT approach makes use of a sensitizer in order to transfer photoenergy to an organic substrate under mild conditions.

Although numerous organosensitizers,^15–18^ ruthenium^19–21^ and other transition metal-based photocatalysts^22–25^ have been deployed in EnT photocatalysis, iridium complexes remain the state-of-the-art in this area. This is due to the complexes' significantly long excited triplet state lifetime (μs), high quantum yields and ligand design opportunities permitting to modify their properties. But most importantly, they possess impressively high triplet energy (*E*_T_) values, which allow excitation of a wide range of organic molecules.^26^ Nevertheless, the triplet energy level (*E*_T_) of 64 kcal mol^−1^ has represented a plateau until very recent advances for iridium systems.^27^ In addition to this limitation, the cationic nature of iridium complexes and the corresponding low solubility in many organic solvents often prevents the use of first choice sustainable solvents for catalytic reactions.^28^ In the context of solvent compatibility, the exact amount required to enable catalysis is oftentimes overestimated. This is of no small consequence as the price of later generation iridium photocatalysts is exceedingly high.^29^ For these reasons, it would prove beneficial to provide the community with more affordable but as efficient alternatives.

**Fig. 1:**
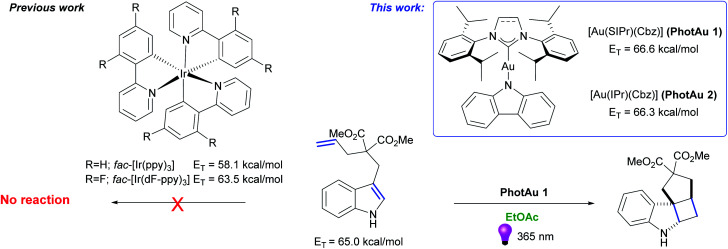
State-of-the-art Ir photosensitizers and novel gold photosensitizers [Au(SIPr)(Cbz)] (PhotAu 1) and [Au(IPr)(Cbz)] (PhotAu 2).

In our recent work, a simple and sustainable procedure towards carbene–metal–amido (CMA) complexes was developed.^30^ Noteworthy, two gold carbazolyl complexes – [Au(SIPr)(Cbz)] (PhotAu 1) (SIPr: [*N*,*N*-bis(2,6-diisopropylphenyl)imidazolin-2-ylidene]; Cbz = carbazolyl) and [Au(IPr)(Cbz)] (PhotAu 2) (IPr = [*N*,*N*-bis(2,6-diisopropylphenyl)imidazol-2-ylidene]) showed intriguing photophysical properties: remarkable long lifetimes in solution – 266 and 335 μs respectively – and impressively high energy emission. Preliminary photocatalytic reaction of intermolecular cycloaddition of dicinnamyl ether proceeded smoothly under non-optimized conditions. Full conversion was achieved after 4 hours with 5 mol% of PhotAu 1 as photocatalyst. Upon close examination of the emission spectra (see Fig. S2 and S3†) of these carbazolyl complexes, we observed that their *E*_T_ values significantly exceed the 64 kcal mol^−1^ barrier that was the upper limit of Ir-based systems at the time this study was initiated. For the two systems initially studied, *E*_T_ values of 66.3 and 66.6 kcal mol^−1^ for IPr and SIPr congeners respectively were obtained from emission spectra. Taking into account these impressing results, the ease of synthesis of such complexes *via* the operationally simple and cost-effective weak base route^31–33^ and the market price of gold *vs.* iridium, we were eager to investigate in detail the photocatalytic behavior of these two complexes in reactions mediated by iridium and possibly in reactions where iridium photocatalysts had proven thus far ineffective (Fig. 1). Therefore, we present our initial photocatalytic findings as well as some advantages and limitations of our new gold photocatalysts.

## Results and discussion

We initiated our studies with the [2 + 2] cycloaddition reaction involving styrenes, the benchmark in metal-mediated photocatalysis occurring *via* EnT initially reported by Yoon.^34^ Originally, 1 mol% of [Ir(dF(CF_3_)ppy)_2_(dtbbpy)][PF_6_] was used as sensitizer and the reaction proceeded in very dilute DMSO solution (0.01 M). Ruthenium sensitizers were unable to mediate this reaction as triplet energies of styrenes are estimated *ca.* 60 kcal mol^−1^ (ref. 35 and 36) which prove higher than that of well-known Ru-based photocatalysts.^19,20,37^

Our optimization began with decreasing the catalyst loading and shortening the reaction time while using irradiation at 365 nm to establish best conditions. Surprisingly, in the case of both PhotAu 1 and PhotAu 2, we were able to reduce the catalyst amount to 1 mol% and the reaction time to 1 hour (Table 1, for details see Table S1†). Further decreasing reaction time and/or catalyst loading led to decreased conversions (Table 1, entries 3 and 4). The initial choice of the irradiation wavelength was based on absorption spectra of the photocatalysts. However, a 380 nm lamp, closer to the visible light range also proved efficient, but required longer reaction times (Table 1, entry 5). Nevertheless, this wavelength variation shows the applicability of irradiation at longer wavelength.

**Table 1:** Optimization of reaction conditions

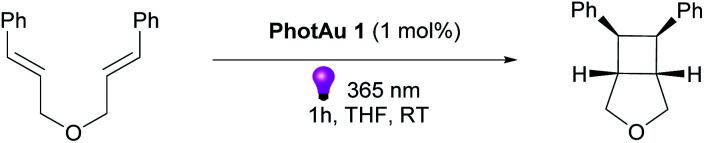		
Entry	Deviations from standard conditions	Conversion^a^ (%)
1	None	98
2	PhotAu 2 instead of PhotAu 1	98
3	30 min	94
4	0.5 mol% PhotAu 1	95
5	380 nm LED	80 (97^b^)
6	405 nm LED	20
7	Me-THF	98
8	EtOAc	99
9	i-PrOAc	96
10	Acetone	96
11	MeCN	89
12	MeOH	99
13	Without PhotAu 1	<10
14	In darkness	<10

a Conversions were determined by GC using dodecane as internal standard and are the average of 2 reactions.

b 2 h reaction time.

We next investigated the influence of solvents on the model reaction, as this remains one of the most notable limitations of iridium sensitizers. To our delight, the conversion proved nearly unaffected by the reaction medium being commonly used organic solvents, including greener solvents such as MeOH, i-PrOAc and EtOAc (Table 1, entries 8, 9 and 12). Control experiments confirmed the need for both sensitizer and irradiation for the reaction to proceed (Table 1, entries 13 and 14).

**Scheme 1:**
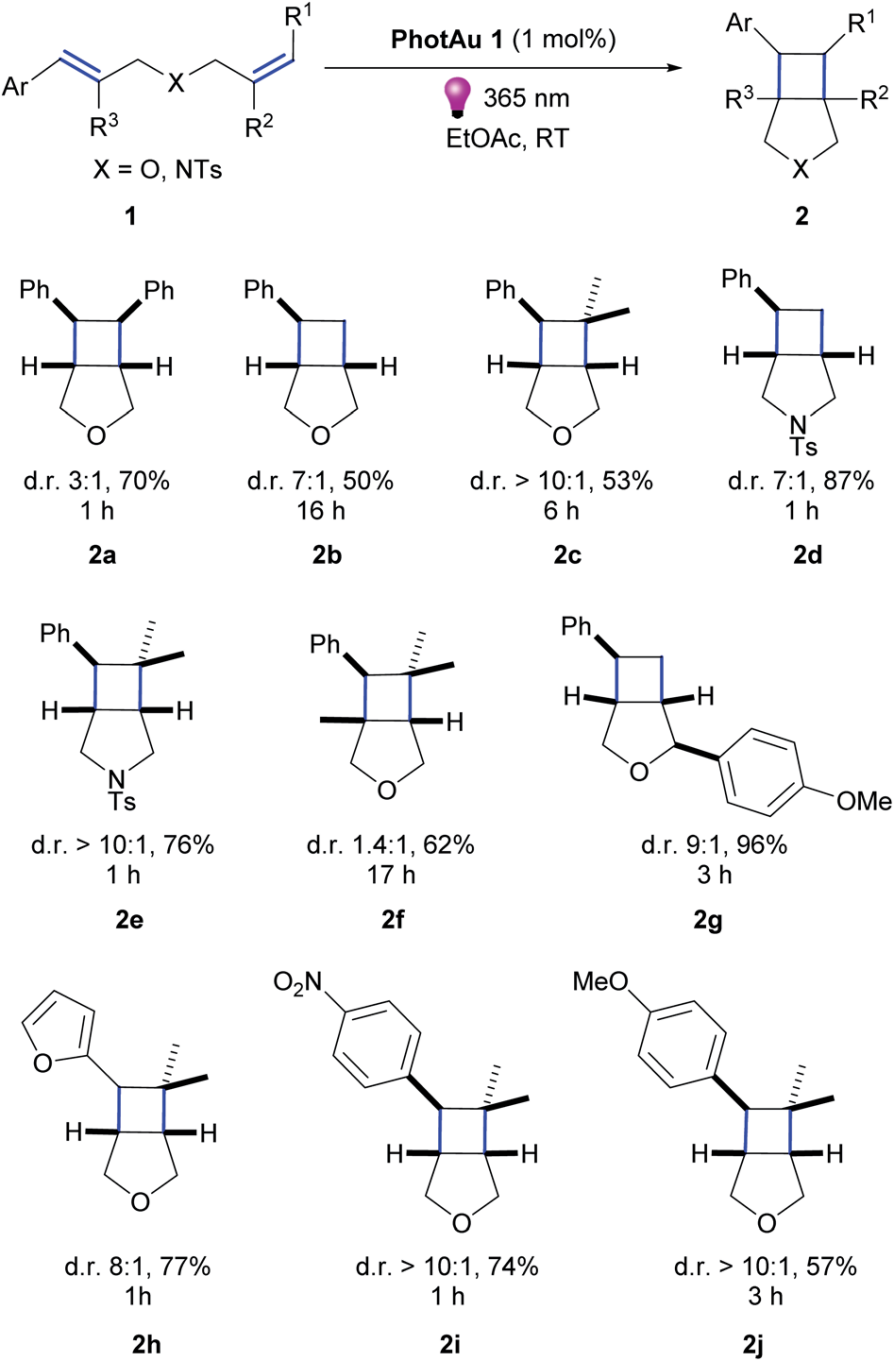
Substrate scope of the [2 + 2] cycloaddition of diallyl ethers and *N*-tosylamides. Isolated yields are average of 2 reaction runs. d.r. were determined by ^1^H NMR of reaction mixtures prior to isolation.

Having optimized conditions in hand, we explored the scope of the reaction with various diallyl ethers.(Scheme 1).The reaction was successfully performed with various styrenes (2a–c), including ones bearing electron withdrawing and electron donating groups (2i–j). The presence of *gem*-dimethyl groupings on a double-bond (2c, e, f, h–j) as well as methyl group in the β-position of styrene (2f) and PMP (*p*-methoxyphenyl) group in the β-position of the allyl fragment (2g) were well tolerated. Vinylfuran reacted rapidly leading to product in good 77% yield (2h). Noteworthy, diallyl *N*-tosylamides also proved suitable substrates for the cycloaddition reaction (2d, e).

Product diastereomeric ratios did not differ significantly from those obtained using iridium photocatalysts.^34^ Noteworthy, for all previously reported examples using Ir photocatalysts, the use of our gold sensitizer under our reaction conditions significantly decreased reaction time: for instance, from 72 h to 17 h for 2f and from 12 h to 1 h for 2e. However, the observed effects of substituents near non-sensitized double bond on the reaction time remain unclear. Such variations in reaction times have been noticed previously,^22,34^ and we gather that it is the result of thermodynamic or/and kinetic factors in post-EnT stages of cyclization .

The use of PhotAu 1 as photocatalyst in this suite of substrates has allowed us to perform the intramolecular cycloaddition of styrenes in a more sustainable manner using green solvent, much less expensive sensitizer and shorter reaction times compared to literature reports.

Keeping in mind these exciting results and the knowledge of high *E*_T_ values of our PhotAu catalysts, we searched for challenging reactions which proved inaccessible with known state-of-the-art iridium sensitizers. One such example is the intramolecular [2 + 2] cycloaddition of indoles.

**Scheme 2:**
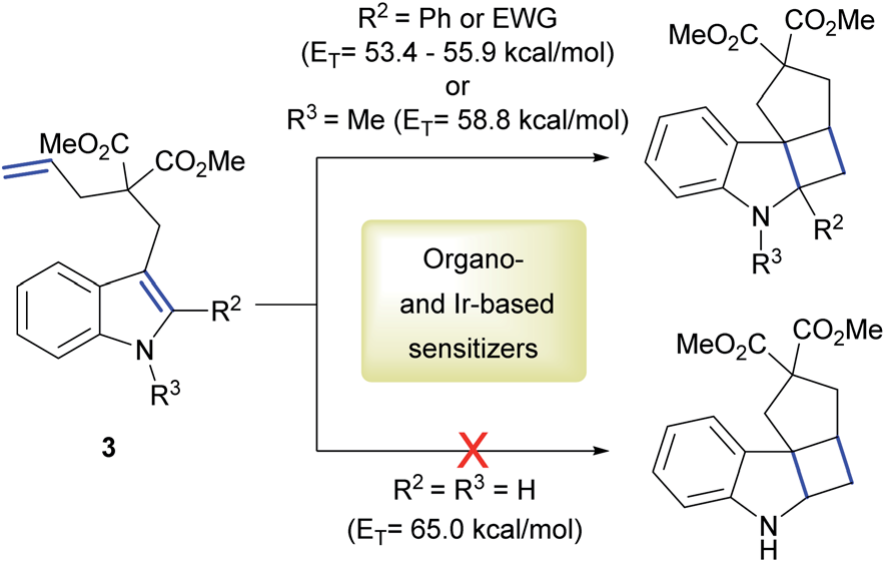
Previously reported intramolecular [2 + 2] cycloadditions of indoles 3.

Polycyclic spiroindolines are privileged scaffolds present in numerous naturally occurring alkaloids.^38^ Recently several methods of light-mediated dearomatization of indoles have been reported leading to these important skeletons.^18,39,40^ However, pre-functionalization has proven essential to induce the process. It was established that phenyl and EWG substituents at the C2 position of the indole, as well as its acylation significantly reduce the *E*_T_ of the molecule, therefore facilitating the reaction. Triplet energy of unsubstituted indole 3 was estimated by You and co-workers at 65 kcal mol^−1^.^39^ This value clarified the lack of reactivity using organosensitizer, Ru-based sensitizers and state-of-the-art Ir-based sensitizers. We reasoned this reaction could represent a benchmark for the direct comparison of our catalytic system with previously reported sensitizers (Scheme 2).

**Scheme 3:**
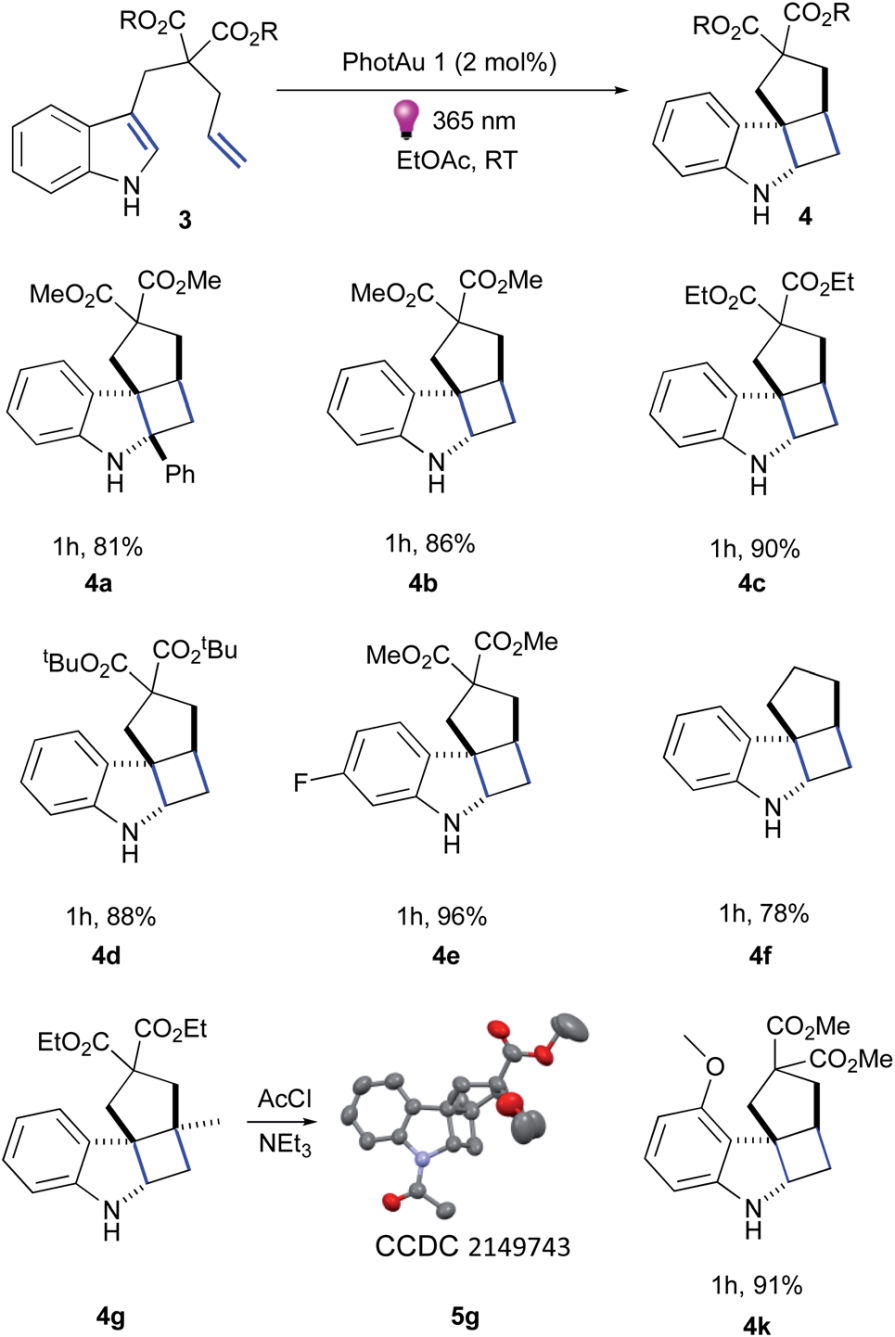
Substrate scope of indole derivatives. Isolated yields are average of 2 runs.

Firstly, we examined the conversion of 3a into 4a, which was successfully performed by You *et al.* using 4 mol% of [Ir(dF(CF_3_)ppy)_2_(dtbbpy)][PF_6_] in the mixture of DCM/CH_3_CN (3 : 1).^39^ The reaction proceeded rapidly with the use of 2 mol% PhotAu 1 or PhotAu 2 in EtOAc (Scheme 3). Full conversion was reached after 1 hour compared to the previously reported 48 hours with Ir. Inspired by this result, we probed the cycloaddition of challenging unprotected indole 3b under similar reaction conditions. To our delight, full conversion was achieved in 1 hour yielding 4b as sole diastereomer. Modifying the ester groups to ethyl or bulky *tert*-butyl was well tolerated (4c, 4d). We also confirmed that the diester-substituted linker is not essential for ring-closing to occur with the assistance of the Thorpe−Ingold effect^41^ (4f) which was also observed previously for *N*-acetyl congener of indole 3b.^18^ The 6-fluoro-substituted indole proceeded to the cyclized product smoothly (4e) as well as indole bearing electron-donating 4-methoxy group (4k). Interestingly, 5-methoxy and 5-bromo congeners did not react even after increasing the catalyst loading and reaction time. We reasoned that this behaviour can be attributed to the short lifetime of their excited-state species, as calculated *E*_T_ values do not exceed the one of PhotAu 1 and their electrochemical properties are similar to those of indoles 4b and 4e (see ESI† for detailed information). The reaction proved slower for substrates bearing a methyl group at the external double-bond, a behavior also previously noted for C2-substituted indoles.^18,39^ Nonetheless, the product was obtained in high yield after a slight increase of the reaction time (4g).

Fused tetracyclic indoline 4g was acylated and XRD suitable crystals were obtained for 5g by slow evaporation from CHCl_3_/hexane solution to unambiguously confirm its structure (Fig. S16†).

**Fig. 2:**
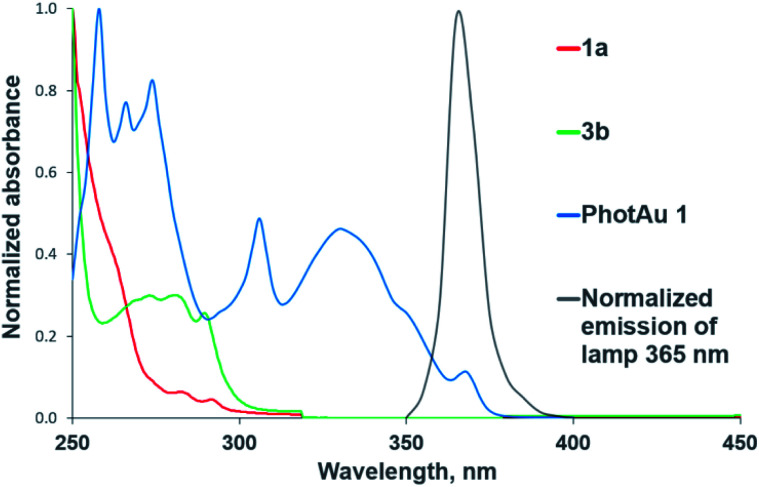
Normalized UV-Vis absorption spectra of substrates and sensitizer and normalized emission spectrum of the 365 nm LED lamp.

The need for both light and photocatalyst to perform the reaction was confirmed by control experiments in darkness and with irradiation but without photocatalyst for both cycloaddition reactions. The UV-Vis absorption spectra of 1a, 3b and PhotAu 1 confirm that only the gold complex absorbs light in the emission region of the spectrum of the lamp employed (Fig. 2). While the photocatalytic nature of the process is unquestionable, distinguishing between electron transfer and triplet–triplet energy transfer mechanisms was next addressed.

As mentioned previously, organic, ruthenium-based and state-of-the-art iridium-based photocatalysts have been examined in the intramolecular cyclization of 3b, however, none of these led to product formation.^39^ In addition, no correlation between redox potentials of the examined sensitizers and reactivity was found for C2-phenyl substituted 3a. We performed cyclic voltammetry for both PhotAu 1 and PhotAu 2 to gain insight into the possibility of the involvement of an electron transfer mechanism. Two irreversible oxidations were noted in the cyclic voltammograms of both photocatalysts making electron transfer processes highly unlikely (Fig. S5 and S6†).^5^

**Fig. 3:**
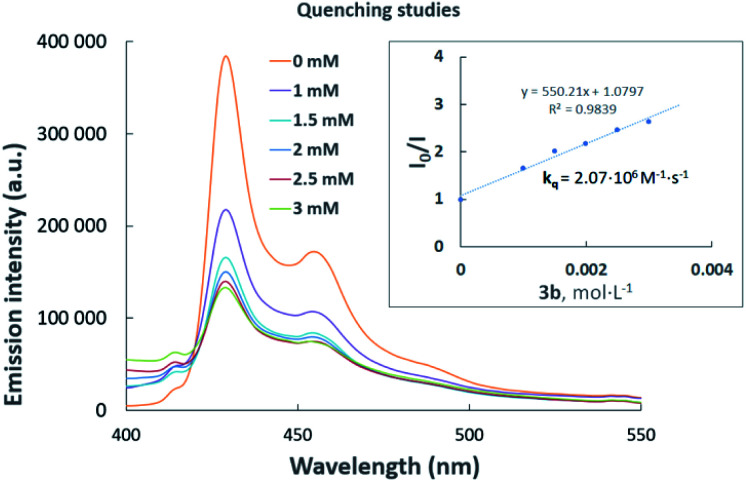
Luminescence quenching studies. Stern–Volmer plot (inset) of the intramolecular cyclization of 3b.

**Scheme 4:**
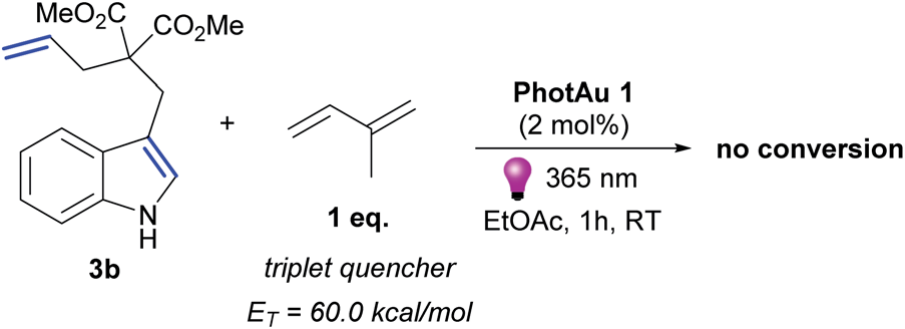
Control experiment with triplet quencher.

Luminescence quenching studies were conducted to probe the interaction between photocatalyst PhotAu 1 and indole 3b (Fig. 3). The observed quenching of the photocatalyst emission in the presence of different amounts of 3b was used to generate a Stern–Volmer plot and thereby determine its quenching constant. Since the intrinsic lifetime of the triplet state of PhotAu 1 is known, we determined the quenching rate constant *k*_q_ to be 2.07 × 10^6^ M^−1^ s^−1^. This value is significantly lower than typical rates of diffusion, which implies that the energy transfer event is the rate-liming step occurring in Dexter energy transfer. Noteworthy, the cyclization reaction of 3b was completely inhibited when performed in the presence of isoprene, a presumably more efficient triplet quencher due to its lower *E*_T_ value of 60 kcal mol^−1^ (Scheme 4).^42^

In order to probe possible radical chain mechanism an on/off experiment was performed, showing the absence of any conversion without irradiation (Fig. S12†). Taking into account that Cismesia and Yoon pointed out that on/off experiments can be misleading for evaluation of chain process,^43^ we also determined the reaction quantum yield (see ESI†). The quantum yield of the [2 + 2] cycloaddition of 3b was found to be 0.11. All observations support the cycloaddition reactions proceeding through a triplet–triplet energy transfer (TTEnT) mechanism.

## Conclusions

In conclusion, we have reported the use of [Au(SIPr)(Cbz)] (PhotAu 1) and [Au(IPr)(Cbz)] (PhotAu 2) as sensitizers for [2 + 2] cycloaddition of diallyl ethers and *N*-tosylamides. The reactions proceed in environmentally friendlier solvent and in much shorter times than previously reported with iridium photocatalysts. The gold sensitizers are compatible with other green solvents such as Me-THF, MeOH, acetone and iPrOAc as well as with 380 nm irradiation. High *E*_T_ value of PhotAu 1 (66.6 kcal mol^−1^) allowed for the previously unsuccessful photocatalytic intramolecular [2 + 2] cycloaddition of indoles 3 yielding products in impressively short reaction time. Quenching studies, on/off experiment, determination of the quantum yield of the reaction and the cyclic voltammograms of photocatalysts support the existence of a triplet–triplet energy transfer (TTEnT) mechanism.

We have shared here our initial findings and photocatalyst development on architectures known to be quite versatile in Au(i) catalysis.^44^ Ongoing efforts are aimed at synthesizing and developing additional members of this photocatalyst family, to understand their inner workings and to gauge to what degree gold can potentially act *in lieu* of (or in a complementary manner to) the workhorse ruthenium and iridium state-of-the-art photocatalysts.

## Data availability

The ESI† contains method description, product characterization data, NMR and absorption spectra, cyclic voltammograms and computational details.

## Author contributions

EAM, VAV, CSJC and SPN conceived and designed the project. EAM and VAV performed all photocatalytic reactions, mechanistic studies and synthesis of indole substrates. SGG and FB synthesized ether and tosylamide substrates. SGG performed DFT calculations, FB performed cyclic voltammetry. MB and KVH performed the XRD measurements and structure analysis. EAM, VAV and SPN wrote the manuscript with input from all authors.

## Conflicts of interest

There are no conflicts to declare.

## Supplementary Material

SC-013-D2SC00864E-s001

SC-013-D2SC00864E-s002
