# Supplementary material for: Energy transfer (EnT) photocatalysis enabled by gold-N-heterocyclic carbene (NHC) complexes
Source: Chem Sci. 2022 May 18;13(23):6852–7. doi: 10.1039/d2sc00864e (PMC9200118; doi:10.1039/d2sc00864e)

Electronic Supplementary Material (ESI) for Chemical Science.
This journal is © The Royal Society of Chemistry 2022

Supporting Information

Energy transfer (EnT) photocatalysis enabled by Gold-N-heterocyclic carbene (NHC) complexes

Ekaterina. A. Martynova†, Vladislav. A. Voloshkin†, Sébastien G. Guillet, Francis Bru, Marek Beliš, Kristof Van Hecke, Catherine S. J. Cazin, and Steven. P. Nolan*

Table of Contents

General information 3
Photophysical properties of gold complexes 4
Synthesis of substrates 5
General procedure for synthesis of diallyl ethers and N-tosylamides 1 5
General procedure for synthesis of indoles 3 7
General procedure for photocatalytic [2+2] cycloaddition of diallyl ethers and N-tosyl amides .. 12
General procedure for photocatalytic cycloaddition of indoles 14
Functionalization of indole product 17
Mechanistic studies 18
Absorption studies 18
Electrochemical studies 18
On/Off experiment 22
Quenching studies and Stern-Volmer plot 23
The quantum yield of the reaction with indole 3b 24
Reaction with triplet quencher 26
DFT calculations 27
X-Ray crystal data 35
References 36
NMR spectra 37

General information

Unless otherwise noted all reactions were performed in anhydrous/dried over molecular sieves and degassed solvents. All organic reagents were purchased and used as received without further purification unless otherwise stated.

^{1}H, ^{13}C and ^{19}F nuclear magnetic resonance (NMR) spectra were recorded on a Bruker Avance 300, 400 or 500 MHz spectrometers at 298 K. Chemical shifts (ppm) in ^{1}H and ^{13}C are referenced to the residual solvent peak ($CDCl_3$: δ_H = 7.26 ppm, δ_C = 77.16 ppm). Coupling constants (J) are given in hertz. Abbreviations used in the designation of the signals: s = singlet, br s = broad singlet, d = doublet, br d = broad doublet, dd = doublet of doublets, dt = doublet of triplets, ddt = doublet of doublet of triplets, m = multiplet, q = quadruplet, br q = broad quadruplet, dq = doublet of quadruplets.

Both [Au(SIPr)(Cbz)] and [Au(IPr)(Cbz)] were synthesized according to previously reported procedure.[1]

Non-commercially available allyl alcohols (for substrates **1g**, **1h** and **1j**) were synthesized according to reported procedures.[2–4]

Absorption spectra were recorded on Perkin Elmer LAMBDA^{TM} 950 spectrophotometer using quartz cuvettes.

Luminescence spectra for Stern-Volmer analysis were recorded in quartz cuvettes on FLS 920 Edinburgh spectrofluorometer, equipped with 450W Xenon lamp as an excitation source and PMT detector.

Photocatalytic experiments were performed in EvoluChem^{TM} PhotoRedOx Box by HepatoChem, equipped with an EvoluChemTM LED. 365PF (365 nm, 18 W, 9 mW/cm²), 380PF (380 nm, 18 W, 8 mW/cm²) and 405PF (405 nm, 18W, 28 mW/cm^2) lamps were used.

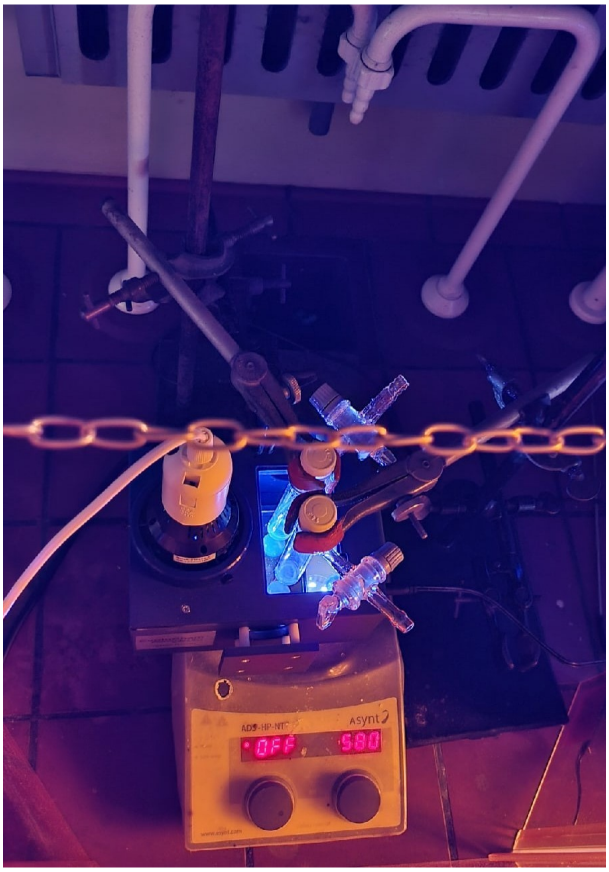

Figure S1. The photocatalytic setup in Nolan group

Electrochemical experiments were conducted in argon-filled glovebox using a Metrohm Autolab M204 potentiostat and screen-printed DRP-550 electrodes with a platinum working electrode, platinum auxiliary electrode and silver reference-electrode. Data was recorded using Autolab NOVA software.

Photophysical properties of gold complexes

The absorption, excitation and emission spectra were previously reported by our research group.[1]

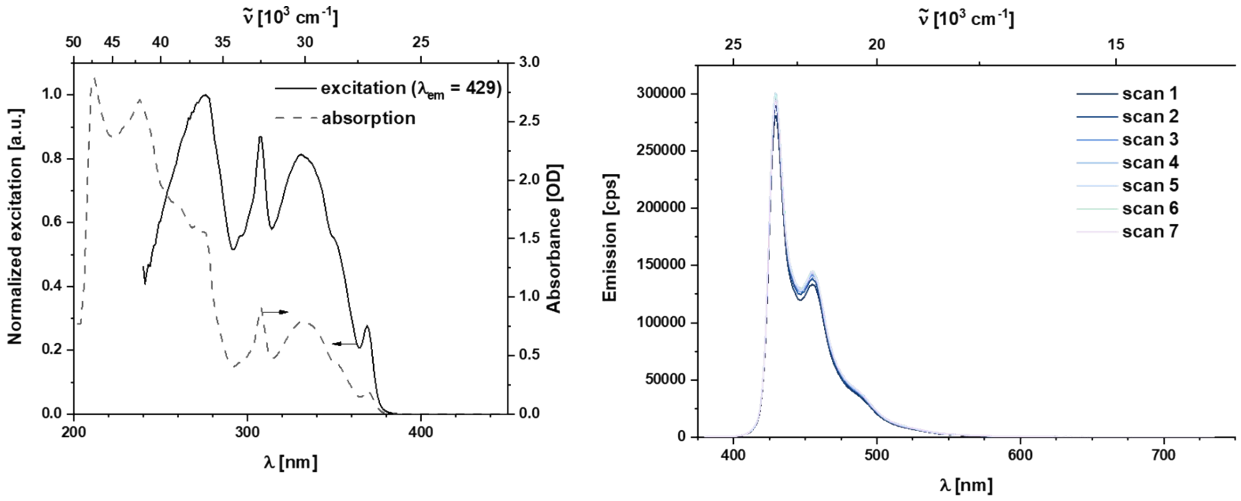

Figure S2. PL spectra of [Au(SIPr)Cbz] (**PhotAu 1**):

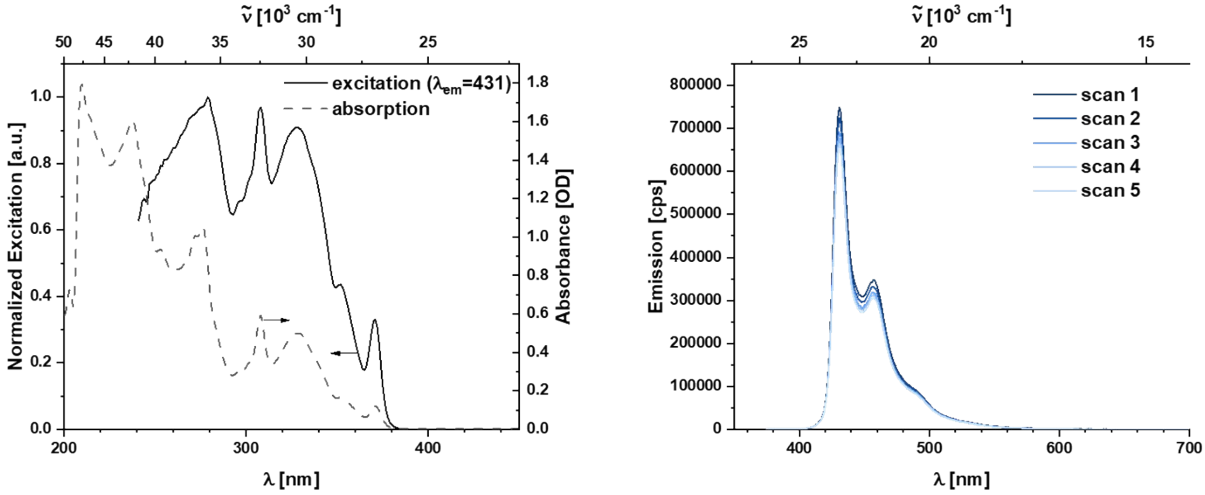

Figure S3. PL spectra of [Au(IPr)Cbz] (**PhotAu 2**):

Synthesis of substrates

General procedure for synthesis of diallyl ethers and N-tosylamides 1

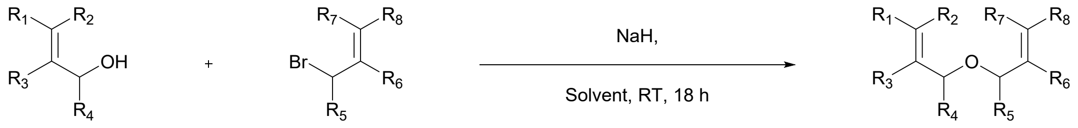

General procedure A for the synthesis of diallyl ethers
In an oven dried 100 mL flask was prepared a suspension of 60% in oil NaH (12 mmol, 1.2 eq) in 15 mL of THF. The suspension was cooled to 0 °C, and a solution of the corresponding alcohol or tosylamide (10 mmol, 1 eq) in 15 mL of THF was added dropwise. The mixture was stirred at room temperature for 30 min before dropwise addition of the allyl bromide (11 mmol, 1.1 eq). The reaction was stirred 18h at room temperature (or more, until TLC analysis showed complete disappearance of the limiting reagent). The reaction was stopped by addition of saturated NH_4Cl solution. The phases were separated, and the aqueous phase was extracted twice with EtOAc. The combined organic phases were washed with water and brine, dried over $MgSO_4$, and concentrated by rotary evaporation. The residue was purified by flash-column chromatography (petroleum ether/ethyl acetate).

General procedure B for the synthesis of diallyl ethers
In an oven dried 100 mL flask the allylic alcohol (10 mmol, 1 eq) and the allylic bromide (12 mmol, 1.2 eq) were dissolved in DMF. The suspension was cooled to 0 °C, and 60% in oil NaH (12 mmol, 1.2 eq) was added portion wise. The reaction was stirred 18h at room temperature (or more, until TLC analysis showed complete disappearance of the alcohol). The reaction was stopped by addition of saturated NH_4Cl solution. The phases were separated, and the aqueous phase was extracted twice with EtOAc. The combined organic phases were washed with water and brine, dried over $MgSO_4$, and concentrated by rotary evaporation. The residue was purified by flash-column chromatography (petroleum ether/ethyl acetate).

((1E,1'E)-oxybis(prop-1-ene-3,1-diyl))dibenzene (**1a**)[5]

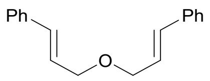

Synthesized from cinnamyl alcohol and cinnamyl bromide following general procedure A. Yield: 86 %.

^{1}H NMR (300 MHz, $CDCl_3$) δ (ppm) 7.33 (d, J = 7.0 Hz, 4 H), 7.24 (t, J = 7.0 Hz, 4 H), 7.20-7.15 (m, 2 H), 5.57 (d, J = 16.0 Hz, 2 H), 6.25 (dt, J_d = 16.0 Hz, J_t = 6.0 Hz, 2 H), 4.14 (dd, J = 6.0 Hz, J = 1.4 Hz, 4 H).

(E)-(3-(allyloxy)prop-1-en-1-yl)benzene (**1b**)[6]

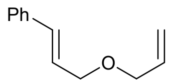

Synthesized from cinnamyl alcohol and allyl bromide following general procedure A. Yield: 74 %.

^{1}H NMR (300 MHz, $CDCl_3$) δ (ppm) 7.30 (d, J = 7.0 Hz, 2 H), 7.22 (t, J = 7.6 Hz, 2 H), 7.18-7.10 (m, 1 H), 6.53 (d, J = 16.0 Hz, 1 H), 6.21 (dt, J_d = 16.0 Hz, J_t = 6.0 Hz, 1 H), 5.87 (ddt, J_d = 10.4 Hz, J_d = 6.8 Hz, J_t = 5.6 Hz, 1 H), 5.23 (dq, J_d = 17.2 Hz, J_q = 1.6 Hz, 1 H), 5.12 (dq, J_d = 10.4 Hz, J_q = 1.4 Hz, 1 H), 4.07 (dd, J = 6.0 Hz, J = 1.5 Hz, 2 H), 3.95 (dt, J_d = 5.6 Hz, J_t = 1.4 Hz, 2 H).

(E)-(3-((3-methylbut-2-en-1-yl)oxy)prop-1-en-1-yl)benzene (**1c**)[5]

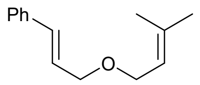

Synthesized from cinnamyl alcohol and 1-bromo-3-methyl-prop-2-ene following general procedure A. Yield: 91 %.

^{1}H NMR (300 MHz, $CDCl_3$) δ (ppm) 7.35 (d, J = 7.1 Hz, 2H), 7.27 (t, J = 7.2 Hz, 2H), 7.22-7.15 (m, 1H), 6.57 (d, J = 16.0 Hz, 1H), 6.27 (dt, J_d = 16.0, J_t = 6.1 Hz, 1H), 5.37 (t-hept, J_t = 7.0 Hz, J_{hept} = 1.4 Hz, 1H), 4.10 (dd, J = 6.0, J = 1.4 Hz, 2H), 3.98 (d, J = 7.0 Hz, 2H), 1.73 (s, 3H), 1.65 (s, 3H).

N-allyl-N-cinnamyl-4-methylbenzenesulfonamide (**1d**)[7]

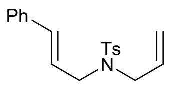

Synthesized from N-cinnamyl-tosylamide[2] and allyl bromide following general procedure A. Yield: 49 %.

^{1}H NMR (300 MHz, $CDCl_3$) δ (ppm) 7.73(d, J = 8.3 Hz, 2H), 7.34–7.20(m, 7H), 6.41(d, J = 15.9 Hz, 1H), 5.94(dt, J_d = 15.6 Hz, J_t = 6.9 Hz, 1H), 5.73–5.57(m, 1H), 5.22–5.11(m, 2H), 3.96(d, J = 6.9 Hz, 2H), 3.85(d, J = 6.2 Hz, 2H), 2.42 (s, 3H).

N-cinnamyl-4-methyl-N-(3-methylbut-2-en-1-yl)benzenesulfonamide (**1e**)[5]

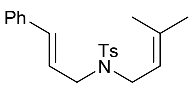

Synthesized from N-cinnamyl-tosylamide[3] and 1-bromo-3-methyl-prop-2-ene following general procedure A. Yield: 72 %.

^{1}H NMR (300 MHz, $CDCl_3$) δ (ppm) 7.72 (d, J = 8.3 Hz, 2H), 7.33-7.21 (m, 7H), 6.41 (d, J = 15.8 Hz, 1H), 5.97 (dt, J_d = 15.8 Hz, J_t = 6.7 Hz, 1H), 5.03 (t-hept, J_t = 7.1 Hz, J_{hept} = 1.4 Hz, 1H), 3.93 (d, J = 6.7 Hz, 2H), 3.82 (d, J = 7.1 Hz, 2H), 2.43 (s, 3H), 1.66 (s, 3H), 1.57 (s, 3H).

(E)-1-(3-((3-methylbut-2-en-1-yl)oxy)prop-1-en-1-yl)-4-nitrobenzene (**1f**)[5]

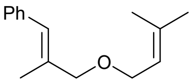

Synthesized from 2-methyl-cinnamyl alcohol and 1-bromo-3-methyl-prop-2-ene following general procedure B. Yield: 99 %.

^{1}H NMR (300 MHz, $CDCl_3$) δ (ppm) 7.33 (t, J = 7.6 Hz, 2H), 7.30-7.27 (m, 2H), 7.21 (t, J = 6.8 Hz, 1H), 6.51 (s, 1H), 5.41 (t-hept, J_t = 7.0 Hz, J_{hept} = 1.4 Hz, 1H), 4.02 (d, J = 1.0 Hz, 2H), 4.00 (d, J = 7.0 Hz, 2H), 1.90 (d, J = 1.4 Hz, 3H), 1.77 (s, 3H), 1.70 (s, 3H).

1-(1-(cinnamyloxy)allyl)-4-methoxybenzene (**1g**)[5]

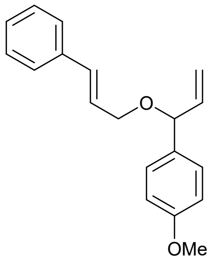

Synthesized from 1-(4-methoxyphenyl)prop-2-en-1-ol[3] and cinnamyl bromide following general procedure B. Yield: 75 %.

^{1}H NMR (300 MHz, $CDCl_3$) δ (ppm) 7.44-7.19 (m, 7H), 6.90 (dt, J_d = 8.8 Hz, J_t =2.5 Hz, 2H), 6.59 (d, J = 16.0 Hz, 1H), 6.31 (dt, J_d = 16.0 Hz, J_t = 6.0 Hz, 1H), 5.99 (ddd, J = 17.2 Hz, J = 10.3 Hz, J = 6.6 Hz, 1H), 5.28 (dt, J_d = 17.2 Hz, J_t = 1.4 Hz, 1H), 5.21 (dt, J_d = 10.3 Hz, J_t = 1.4 Hz, 1H), 4.83 (d, J = 6.5 Hz, 1H), 4.13 (dt, J_d = 6.0 Hz, J_t = 1.3 Hz, 2H), 3.81 (s, 3H).

(E)-2-(3-((3-methylbut-2-en-1-yl)oxy)prop-1-en-1-yl)furan (**1h**)[5]

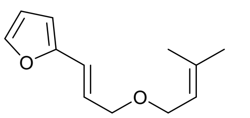

Synthesized from furyl-allyl alcohol[5] and 1-bromo-3-methyl-prop-2-ene following general procedure B. Yield: 80 %.

^{1}H NMR (300 MHz, $CDCl_3$) δ (ppm) 7.34 (d, J = 1.6 Hz, 1H), 6.44 (dt, J_d = 15.9 Hz, J_t = 1.3 Hz, 1H), 6.36 (dd, J = 3.3 Hz, J = 1.8 Hz, 1H), 6.28-6.17 (m, 2H), 5.38 (t-hept, J_t = 6.9 Hz, J_{hept} = 1.4 Hz, 1H), 4.10 (dd, J = 5.8 Hz, J = 1.4 Hz, 2H), 4.00 (d, J = 7.0 Hz, 2H), 1.76 (s, 3H), 1.68 (s, 3H).

(E)-1-(3-((3-methylbut-2-en-1-yl)oxy)prop-1-en-1-yl)-4-nitrobenzene (**1i**)[5]

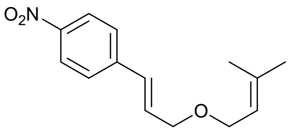

Synthesized from *p*-nitro-phenylallyl alcohol and 1-bromo-3-methyl-prop-2-ene following general procedure B. Yield: 63 %.

^{1}H NMR (300 MHz, $CDCl_3$) δ (ppm) 8.17 (d, J = 8.9 Hz, 2H), 7.50 (d, J = 8.8 Hz, 2H), 6.69 (d, J = 16.0 Hz, 1H), 6.48 (dt, J_d = 16.0, J_t = 5.4 Hz, 1H), 5.39 (t-hept, J_t = 7.0 Hz, J_{hept}= 1.4 Hz, 1H), 4.17 (dd, J = 5.4, J = 1.5 Hz, 2H), 4.04 (d, J = 7.0 Hz, 2H), 1.77 (s, 3H), 1.70 (s, 3H).

(E)-1-methoxy-4-(3-((3-methylbut-2-en-1-yl)oxy)prop-1-en-1-yl)benzene (**1j**)[5]

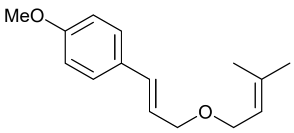

Synthesized from *p*-methyl-phenylallyl alcohol[4] and 1-bromo-3-methyl-prop-2-ene following general procedure A. Yield: 81 %.

^{1}H NMR (300 MHz, $CDCl_3$) δ (ppm) 7.32 (d, J = 8.7 Hz, 2H), 6.85 (d, J = 8.8 Hz, 2H), 6.55 (d, J = 15.9 Hz, 1H), 6.18 (dt, J_d = 15.9 Hz, J_t = 6.3 Hz, 1H), 5.39 (t-hept, J = 7.0 Hz, J_{hept} = 1.4 Hz, 1H), 4.11 (dd, J = 6.3 Hz, J = 1.4 Hz, 2H), 4.01 (d, J = 7.0 Hz, 2H), 3.80 (s, 3H), 1.76 (s, 3H), 1.69 (s, 3H).

General procedure for synthesis of indoles 3

Indole substrate **3f** was synthesized according previously reported procedure.[8]

Indole substrates **3a**-**3e** and **3g**-**3j** were synthesized according to the modified previously reported procedure.[9]

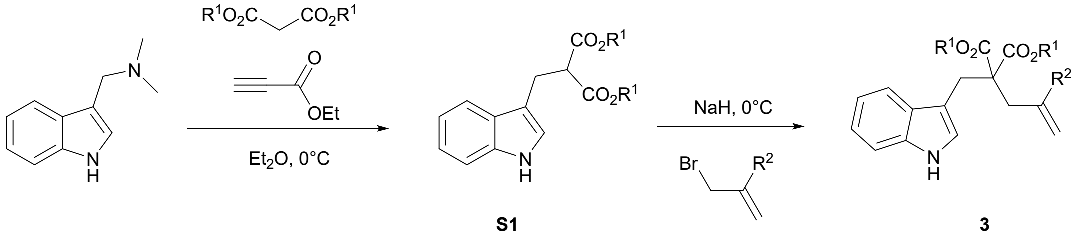

In a round bottom flask, gramine (20 mmol) was suspended into reagent grade Et_2O (10 mL). After the addition of dialkyl malonate (20 mmol), the mixture was cooled to 0°C in ice bath. Ethylpropriolate (1.87 mL, 20 mmol) was added at once, the reaction mixture was allowed to stir for 10 minutes before removing the ice bath. The reaction mixture was left stirring for 16-20 hours at room temperature. After the reaction was complete (monitored by TLC), it was quenched with water (20 mL). The aqueous layer was extracted with EtOAc (30 mL x 3). The combined organic layers were washed with brine, dried over Na_2SO_4, filtered and concentrated by rotary evaporation. Then the residue was purified by silica gel column chromatography (PE/EtOAc) to afford the desired product **S1**.

To a solution of **S1** (1.0 equiv) in THF (10 mL) NaH (60% in oil) (1.2 equiv.) was added, the reaction mixure was allowed to stir for 30 minutes at ambient temperature. After it was cooled down to 0°C in ice bath followed by the dropwise addition of alkenyl bromide (1.2 equiv.). Then the reaction was allowed to stir at ambient temperature for 16-20 hours. After the reaction was complete (monitored by TLC), it was quenched with water (20 mL). The aqueous layer was extracted with EtOAc (30 mL x 3). The combined organic layers were washed with brine, dried over Na_2SO_4, filtered and concentrated by rotary evaporation. Then the residue was purified by silica gel column chromatography (PE/EtOAc) to afford the desired products **3**.

Dimethyl 2-((2-phenyl-1H-indol-3-yl)methyl)malonate (**S1a**)[10]

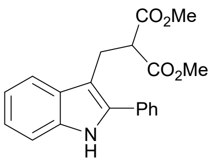

Was synthesized according to general procedure from 2-phenylgramine (2.0 g, 8 mmol), dimethylmalonate (1 mL, 8.8 mmol) and ethyl propiolate (891 μL, 8.8 mmol). Eluent – PE:EtOAc (5:1). Yellow oil, 1.942 g (74%).

^{1}H NMR (300 MHz, $CDCl_3$) δ 8.08 (br s, 1H), 7.65 – 7.55 (m, 3H), 7.52 – 7.42 (m, 2H), 7.42 – 7.33 (m, 2H), 7.24 – 7.09 (m, 2H), 3.76 (dd, *J* = 8.2, 7.1 Hz, 1H), 3.63 – 3.58 (m, 2H), 3.54 (s, 6H).

Dimethyl 2-((1H-indol-3-yl)methyl)malonate (**S1b**)[11]

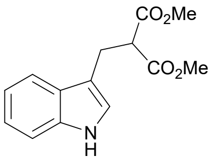

Was synthesized according to general procedure from gramine (3.47 g, 20 mmol), dimethylmalonate (2.12 mL, 22 mmol) and ethyl propiolate (1.868 mL, 22 mmol). Eluent – PE:EtOAc (4:1). Yellow oil, 3.5 g (67%).

^{1}H NMR (400 MHz, $CDCl_3$) δ 8.01 (br s, 1H), 7.65 – 7.53 (m, 1H), 7.35 (dt, *J* = 8.1, 0.9 Hz, 1H), 7.23 – 7.17 (m, 1H), 7.17 – 7.11 (m, 1H), 7.04 (d, *J* = 2.4 Hz, 1H), 3.81 (t, *J* = 7.6 Hz, 1H), 3.70 (s, 6H), 3.40 (dd, *J* = 7.6, 0.7 Hz, 2H).

Diethyl 2-((1H-indol-3-yl)methyl)malonate (**S1c**)[11]

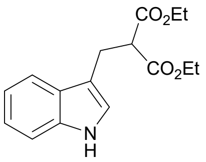

Was synthesized according to general procedure from gramine (3.47 g, 20 mmol), diethylmalonate (2.85 mL, 22 mmol) and ethyl propiolate (1.868 mL, 22 mmol). Eluent – PE:EtOAc (5:1). Pink-ish solid, 4.43 g (77%).

^{1}H NMR (400 MHz, $CDCl_3$) δ 8.06 (s, 1H), 7.66 – 7.57 (m, 1H), 7.39 – 7.31 (m, 1H), 7.23 – 7.16 (m, 1H), 7.16 – 7.10 (m, 1H), 7.04 (d, *J* = 2.4 Hz, 1H), 4.24 – 4.06 (m, 4H), 3.78 (t, *J* = 7.7 Hz, 1H), 3.40 (dd, *J* = 7.7, 0.7 Hz, 2H), 1.21 (t, *J* = 7.1 Hz, 6H).

Di-*tert*-butyl 2-((1H-indol-3-yl)methyl)malonate (**S1d**)[11]

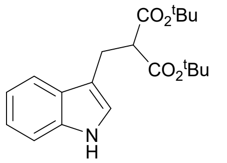

Was synthesized according to general procedure from gramine (3.47 g, 20 mmol), di-*tert*-butylmalonate (4.17 mL, 22 mmol) and ethyl propiolate (1.868 mL, 22 mmol). Eluent – PE:EtOAc (4:1). White solid, 4.43 g (63%).

^{1}H NMR (400 MHz, $CDCl_3$) δ 8.03 (br s, 1H), 7.63 (d, *J* = 7.8 Hz, 1H), 7.34 (dt, *J* = 8.1, 0.9 Hz, 1H), 7.23 – 7.16 (m, 1H), 7.16 – 7.09 (m, 1H), 7.03 (d, *J* = 2.1 Hz, 1H), 3.60 (t, *J* = 7.7 Hz, 1H), 3.30 (dd, *J* = 7.7, 0.7 Hz, 2H), 1.41 (s, 18H).

Dimethyl 2-((6-fluoro-1H-indol-3-yl)methyl)malonate (**S1f**)

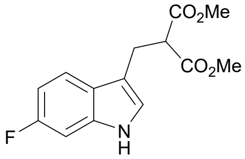

Was synthesized according to general procedure from 5-fluorogramine (1.0 g, 5.2 mmol), dimethylmalonate (654 µL, 5.72 mmol) and ethyl propiolate (580 µL, 5.72 mmol). Eluent – PE:EtOAc (3:1). Yellow oil, 1.2 g (83%).

^{1}H NMR (300 MHz, $CDCl_3$) δ 8.00 (s, 1H), 7.50 (dd, *J* = 8.7, 5.3 Hz, 1H), 7.08 – 6.98 (m, 2H), 6.95 – 6.85 (m, 1H), 3.80 – 3.72 (m, 1H), 3.70 (s, 6H), 3.37 (dd, *J* = 7.6, 0.6 Hz, 2H).

^{13}C NMR (75 MHz, $CDCl_3$) δ 169.7, 160.2 (d, *J* = 237.8 Hz), 136.2 (d, *J* = 12.5 Hz), 122.8 (d, *J* = 3.5 Hz), 119.45 (d, *J* = 10.2 Hz), 108.5 (d, *J* = 24.6 Hz), 97.6 (d, *J* = 26.1 Hz), 52.85, 52.7, 24.7.
^{19}F NMR (377 MHz, $CDCl_3$) δ -121.5.
HRMS (ESI-TOF): Calcd for $C_{14}H_{15}FNO_4$+ $[M+H]^+$ 280.0980; found 280.0948

Dimethyl 2-((5-methoxy-1H-indol-3-yl)methyl)malonate (**S1g**)[12]

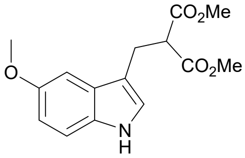

Was synthesized according to general procedure from 5-methoxygramine (2.04 g, 10 mmol), dimethylmalonate (1.26 mL, 11 mmol) and ethyl propiolate (1.15 mL, 11 mmol). Eluent – PE:EtOAc (4:1). Brown oil, 2.5 g (86%).

^{1}H NMR (400 MHz, $CDCl_3$) δ 7.91 (s, 1H), 7.25 – 7.20 (m, 1H), 7.02 (dd, *J* = 8.0, 2.3 Hz, 2H), 6.86 (dd, *J* = 8.8, 2.3 Hz, 1H), 3.87 (s, 3H), 3.79 (t, *J* = 7.7 Hz, 1H), 3.71 (s, 7H), 3.37 (dd, *J* = 7.7, 0.7 Hz, 2H).

Dimethyl 2-((5-bromo-1H-indol-3-yl)methyl)malonate (**S1h**)[12]

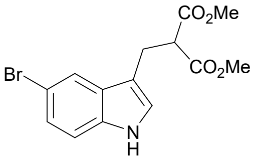

Was synthesized according to general procedure from 6-bromogramine (0.95 g, 3.75 mmol), dimethylmalonate (471 µL, 4.12 mmol) and ethyl propiolate (418 µL, 4.12 mmol). Eluent – PE:EtOAc (2:1). Yellow oil, 1.01 g (79%).

^{1}H NMR (300 MHz, $CDCl_3$) δ 8.08 (s, 1H), 7.74 – 7.67 (m, 1H), 7.31 – 7.16 (m, 2H), 7.05 (d, *J* = 2.4 Hz, 1H), 3.75 (t, *J* = 7.6 Hz, 1H), 3.71 (s, 6H), 3.34 (dd, *J* = 7.6, 0.6 Hz, 2H).

Dimethyl 2-((4-methoxy-1H-indol-3-yl)methyl)malonate (**S1k**)[13]

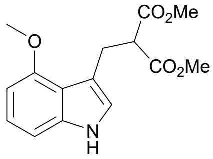

Was synthesized according to general procedure from 4-methoxygramine (2.7 g, 13.2 mmol), dimethylmalonate (1.66 mL, 14.5 mmol) and ethyl propiolate (1.47 mL, 14.5 mmol). Eluent – PE:EtOAc (2:1). Yellow oil, 3.6 g (94%).

^{1}H NMR (300 MHz, $CDCl_3$) δ 7.95 (br s, 1H), 7.08 (t, *J* = 8.0 Hz, 1H), 6.94 (dd, *J* = 8.1, 0.5 Hz, 1H), 6.89 (d, *J* = 2.2 Hz, 1H), 6.49 (d, *J* = 7.7 Hz, 1H), 4.03 (t, *J* = 7.5 Hz, 1H), 3.92 (s, 3H), 3.69 (s, 6H), 3.45 (d, *J* = 7.5 Hz, 2H).

Dimethyl 2-allyl-2-((2-phenyl-1H-indol-3-yl)methyl)malonate (**3a**)[10]

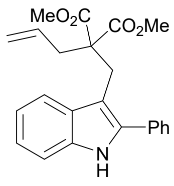

Was synthesized according to general procedure from **S1a** (1.0 g, 3 mmol), NaH (60% in oil) (142 mg, 3.6 mmol) and allyl bromide (431 µL, 3.6 mmol). Eluent – PE:EtOAc (6:1). Yellow solid, 0.494 g (44%).

^{1}H NMR (300 MHz, $CDCl_3$) δ 8.05 (br s, 1H), 7.65 – 7.51 (m, 3H), 7.51 – 7.42 (m, 2H), 7.42 – 7.30 (m, 2H), 7.21 – 7.06 (m, 2H), 5.45 – 5.25 (m, 1H), 4.88 – 4.71 (m, 2H), 3.71 (s, 2H), 3.41 (s, 6H), 2.35 (d, *J* = 7.2 Hz, 2H).

Dimethyl 2-((1H-indol-3-yl)methyl)-2-allylmalonate (**3b**)[11]

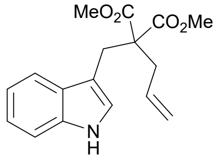

Was synthesized according to general procedure from **S1b** (2.1 g, 8 mmol), NaH (60% in oil) (390 mg, 10 mmol) and allyl bromide (835 µL, 10 mmol). Eluent – PE:EtOAc (5:1). Yellow oil, 0.97 g (40%).

^{1}H NMR (400 MHz, $CDCl_3$) δ 8.12 (br s, 1H), 7.60 – 7.50 (m, 1H), 7.37 – 7.29 (m, 1H), 7.21 – 7.13 (m, 1H), 7.13 – 7.06 (m, 1H), 6.98 (d, *J* = 2.4 Hz, 1H), 5.92 – 5.74 (m, 1H), 5.17 – 5.15 (m, 1H), 5.14 – 5.10 (m, 1H), 3.66 (s, 6H), 3.44 (s, 2H), 2.72 – 2.64 (m, 2H).

Diethyl 2-((1H-indol-3-yl)methyl)-2-allylmalonate (**3c**)[14]

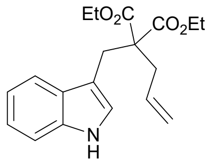

Was synthesized according to general procedure from **S1c** (1.15 g, 4 mmol), NaH (60% in oil) (191 mg, 4.8 mmol) and allyl bromide (413 µL, 4.8 mmol). Eluent – PE:EtOAc (5:1). Yellow oil, 832 mg (63%).

^{1}H NMR (300 MHz, $CDCl_3$) δ 8.08 (br s, 1H), 7.57 (d, *J* = 7.8 Hz, 1H), 7.34 – 7.30 (m, 1H), 7.20 – 7.13 (m, 1H), 7.12 – 7.06 (m, 1H), 7.00 (d, *J* = 2.1 Hz, 1H), 5.93 – 5.74 (m, 1H), 5.16 (s, 1H), 5.14 – 5.05 (m, 1H), 4.23 – 4.02 (m, 5H), 3.42 (s, 2H), 2.74 – 2.59 (m, 2H), 1.20 (t, *J* = 7.1 Hz, 6H).

Di-*tert*-butyl 2-((1H-indol-3-yl)methyl)-2-allylmalonate (**3d**)

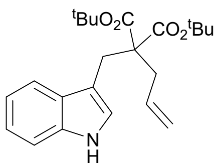

Was synthesized according to general procedure from **S1d** (1.375 g, 4 mmol), NaH (60% in oil) (191 mg, 4.8 mmol) and allyl bromide (413 µL, 4.8 mmol). Eluent – PE:EtOAc (8:1). Yellow oil, 939 mg (61%)

^{1}H NMR (300 MHz, $CDCl_3$) δ 8.04 (br s, 1H), 7.64 (d, *J* = 7.7 Hz, 1H), 7.36 – 7.29 (m, 1H), 7.21 – 7.13 (m, 1H), 7.13 – 7.06 (m, 1H), 7.04 (d, *J* = 2.0 Hz, 1H), 5.92 – 5.72 (m, 1H), 5.14 – 5.11 (m, 1H), 5.11 – 5.05 (m, 1H), 3.34 (d, *J* = 0.6 Hz, 2H), 2.68 – 2.56 (m, 2H), 1.40 (s, 18H).
^{13}C NMR (75 MHz, $CDCl_3$) δ 170.8, 135.8, 133.4, 128.7, 122.9, 122.0, 119.4, 119.3, 118.8, 111.0, 110.7, 81.5, 59.4, 37.4, 28.0, 27.3.
HRMS (ESI-TOF): Calcd for $C_{23}H_{32}NO_4^+$ $[M+H]^+$ 386.2326; found 386.2338.

Dimethyl 2-allyl-2-((6-fluoro-1H-indol-3-yl)methyl)malonate (**3e**)

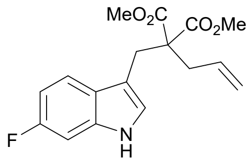

Was synthesized according to general procedure from **S1f** (1.25 g, 4.5 mmol), NaH (60% in oil) (215 mg, 5.4 mmol) and allyl bromide (465 µL, 5.4 mmol). Eluent – PE:EtOAc (3:1). White solid, 569 mg (40%).

^{1}H NMR (300 MHz, $CDCl_3$) δ 8.07 (br s, 1H), 7.44 (dd, *J* = 8.7, 5.3 Hz, 1H), 7.06 – 6.91 (m, 2H), 6.93 – 6.77 (m, 1H), 5.91 – 5.68 (m, 1H), 5.18 – 5.14 (m, 1H), 5.14 – 5.08 (m, 1H), 3.65 (s, 6H), 3.39 (s, 2H), 2.67 (d, *J* = 7.3 Hz, 2H).
^{13}C NMR (75 MHz, $CDCl_3$) δ 171.7, 160.1 (d, *J* = 237.7 Hz), 135.7, 132.9, 124.8, 123.6 (d, *J* = 3.6 Hz), 119.8 (d, *J* = 10.2 Hz), 119.3, 110.3, 108.4 (d, *J* = 24.6 Hz), 97.5 (d, *J* = 26.1 Hz), 59.1, 52.5, 37.6, 28.3.
^{19}F NMR (377 MHz, $CDCl_3$) δ -121.1.
HRMS (ESI-TOF): Calcd for $C_{17}H_{19}FNO_4$ $[M+H]^+$ 320.1293; found 320.1296.

Diethyl 2-((1H-indol-3-yl)methyl)-2-(2-methylallyl)malonate (**3g**)[15]

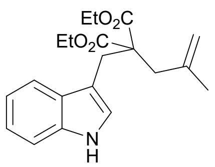

Was synthesized according to general procedure from **S1b** (1.15 g, 4 mmol), NaH (60% in oil) (191 mg, 4.8 mmol) and 3-bromo-2-methylprop-1-ene (481 µL, 4.8 mmol). Eluent – PE:EtOAc (8:1). Yellow oil, 603 mg (44%).

^{1}H NMR (300 MHz, $CDCl_3$) δ 8.05 (br s, 1H), 7.57 (d, *J* = 7.8 Hz, 1H), 7.36 – 7.28 (m, 1H), 7.20 – 7.01 (m, 3H), 4.95 – 4.84 (m, 1H), 4.83 – 4.74 (m, 1H), 4.23 – 3.93 (m, 4H), 3.48 (d, *J* = 0.5 Hz, 2H), 2.77 (s, 2H), 1.72 (s, 3H), 1.16 (t, *J* = 7.1 Hz, 6H).

Dimethyl 2-allyl-2-((5-methoxy-1H-indol-3-yl)methyl)malonate (**3i**)

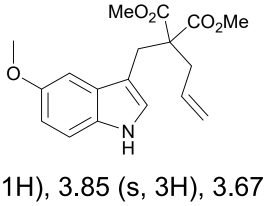

Was synthesized according to general procedure from **S1g** (1.14 g, 4 mmol), NaH (60% in oil) (188 mg, 5 mmol) and allyl bromide (407 µL, 5 mmol). Eluent – PE:EtOAc (5:1). Yellow oil, 0.5 g (38%).

^{1}H NMR (300 MHz, $CDCl_3$) δ 7.98 (s, 1H), 7.21 (d, *J* = 8.8 Hz, 1H), 6.98 (dd, *J* = 14.7, 2.4 Hz, 2H), 6.83 (dd, *J* = 8.8, 2.4 Hz, 1H), 5.95 – 5.72 (m, 1H), 5.16 (s, 1H), 5.14 – 5.02 (m, 1H), 3.85 (s, 3H), 3.67 (s, 6H), 3.39 (s, 2H), 2.69 (d, *J* = 7.3 Hz, 2H).

^{13}C NMR (75 MHz, $CDCl_3$) δ 171.9, 154.2, 133.1, 131.1, 128.7, 124.1, 119.2, 112.5, 111.9, 109.9, 100.9, 59.2, 56.0, 52.5, 37.4, 28.4.
HRMS (ESI-TOF): Calcd for $C_{18}H_{22}NO_5^+$ $[M+H]^+$ 332.1494; found 332.1480.

Dimethyl 2-allyl-2-((5-bromo-1H-indol-3-yl)methyl)malonate (**3j**)

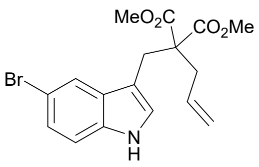

Was synthesized according to general procedure from **S1h** (1.0 g, 3 mmol), NaH (60% in oil) (141 mg, 3.6 mmol) and allyl bromide (305 µL, 3.6 mmol). Eluent – PE:EtOAc (3:1). White solid, 623 mg (55%).

^1H NMR (300 MHz, $CDCl_3$) δ 8.17 (s, 1H), 7.66 (d, *J* = 1.8 Hz, 1H), 7.23 (dd, *J* = 8.6, 1.8 Hz, 1H), 7.16 (d, *J* = 8.6 Hz, 1H), 6.99 (d, *J* = 2.4 Hz, 1H), 5.89 – 5.68 (m, 1H), 5.20 – 5.17 (m, 1H), 5.17 – 5.11 (m, 1H), 3.68 (s, 6H), 3.36 (s, 2H), 2.72 – 2.64 (m, 2H).
^{13}C NMR (75 MHz, $CDCl_3$) δ 171.6, 134.5, 132.8, 129.9, 125.0, 124.7, 121.8, 119.5, 113.0, 112.7, 109.9, 58.9, 52.6, 37.5, 28.2.
HRMS (ESI-TOF): Calcd for $C_{17}H_{19}BrNO_4^+$ $[M+H]^+$ 380.0494; found 380.0480.

Dimethyl 2-allyl-2-((4-methoxy-1H-indol-3-yl)methyl)malonate (**3k**)

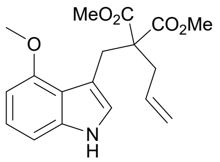

Was synthesized according to general procedure from **S1k** (2.08 g, 7.1 mmol), NaH (60% in oil) (343 mg, 8.5 mmol) and allyl bromide (741 µL, 8.5 mmol). Eluent – PE:EtOAc (3:1). Yellow oil, 600 mg (25%).

^1H NMR (300 MHz, $CDCl_3$) δ 8.07 (br s, 1H), 7.09 – 7.02 (m, 1H), 6.93 (dd, *J* = 8.1, 0.5 Hz, 1H), 6.82 (d, *J* = 2.3 Hz, 1H), 6.47 (d, *J* = 7.7 Hz, 1H), 6.02 – 5.82 (m, 1H), 5.13 – 4.96 (m, *J* = 15.1, 6.6, 5.6 Hz, 2H), 3.90 (s, 3H), 3.68 (s, 2H), 3.65 (s, 6H), 2.67 (d, *J* = 7.3 Hz, 2H).
^{13}C NMR (75 MHz, $CDCl_3$) δ 172.1, 154.7, 137.5, 134.0, 122.7, 122.0, 118.2, 118.1, 110.6, 104.6, 99.6, 60.3, 54.9, 52.2, 37.7, 29.6.

Optimization of photocatalytic conditions

General procedure

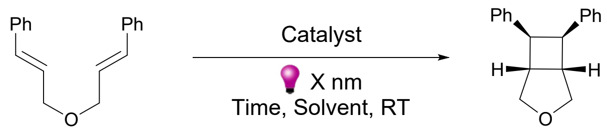

In glovebox diallyl ether **1a** (0.16 mmol, 40 mg) and catalyst were weighted and transferred in 4 mL vial equipped with stirring bar. Degassed solvent was added in reaction mixture and the vial was closed with screwcap and taken out from the glovebox. The vial was placed into photoreactor for indicated time. After this time the vial was opened and dodecane (50 µL) was added to reaction mixture. The conversion was determined using GC. Each reaction was performed twice, and the average conversion is provided.

Table S1. Optimization of reaction conditions.

Entry	Catalyst	Loading (mol%)	Time (h)	Solvent	Lamp	GC conv.%
1	PhotAu 1	5	4	THF	365 nm	99
2	PhotAu 1	5	2	THF	365 nm	99
3	PhotAu 1	5	1	THF	365 nm	99
4	PhotAu 1	5	0.5	THF	365 nm	99
5	PhotAu 1	2.5	0.5	THF	365 nm	99
6	PhotAu 2	2.5	0.5	THF	365 nm	99
7	PhotAu 1	1	1	THF	365 nm	98
8	PhotAu 2	1	1	THF	365 nm	98
9	PhotAu 1	1	0.5	THF	365 nm	94
10	PhotAu 1	0.5	1	THF	365 nm	95
11	PhotAu 2	0.5	1	THF	365 nm	94
12	PhotAu 1	1	0.5	THF	380 nm	69
13	PhotAu 1	1	1	THF	380 nm	80
14	PhotAu 1	1	2	THF	380 nm	97
15	PhotAu 1	1	0.5	THF	405 nm	15
16	PhotAu 1	1	1	THF	405 nm	20
17	PhotAu 1	1	1	Me-THF	365 nm	98
**18**	**PhotAu 1**	**1**	**1**	**EtOAc**	**365 nm**	**99**
19	PhotAu 1	1	1	iPrOAc	365 nm	96
20	PhotAu 1	1	1	Acetone	365 nm	96
21	PhotAu 1	1	1	MeCN	365 nm	89
22	PhotAu 1	1	1	MeOH	365 nm	99
23	PhotAu 1	1	0.5	EtOAc	380 nm	66
24	PhotAu 1	1	1	EtOAc	380 nm	72
25	None	-	1	THF	365 nm	< 10
26	PhotAu 1	1	1	THF	None	< 10

General procedure for photocatalytic [2+2] cycloaddition of diallyl ethers and N-tosyl amides

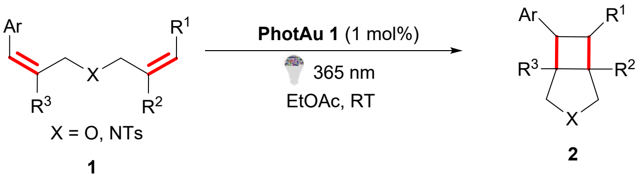

Diallyl ether or N-tosyl amide **1** (0.32 mmol) and [Au(SIPr)(cbz)] (1 mol%, 0.0032 mmol, 2.4 mg) were weighted and transferred in 25 mL Schlenk tube equipped with stirring bar and septum. The tube was filled with Ar or N_2 using 3 vacuum-inert gas cycles. Under the flow of inert gas EtOAc (6 mL) was added in reaction mixture and the tube was closed with septum. Three freeze-thaw cycles were performed to degas the reaction mixture. The tube was placed into photoreactor for indicated time. The aliquots of reaction mixture were collected in the dark under flow of inert gas and evaporated. The ^{1}H NMR of aliquots were recorded to follow the reaction. After full conversion was reached, the solvent was evaporated and crudes of two identical reactions were combined. Diastereomer ratio values were determined using ^{1}H NMR of unpurified reaction mixture. Product was purified using column chromatography with EtOAc-PE mixture as eluent.

6,7-Diphenyl-3-oxabicyclo[3.2.0]heptane (**2a**)[5]

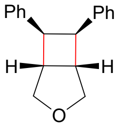

Was synthesized according to general procedure from **1a**. Reaction time – 1 hour. Eluent PE-EtOAc (15:1). Oil, 112.1 mg (d.r. 3:1) (Average yield of two runs – 70%).

(1R*,5S*,6R*,7S*) Diastereomer (major product): ^{1}H NMR (300 MHz, $CDCl_3$) δ 7.12 – 7.04 (m, 4H), 7.04 – 6.98 (m, 2H), 6.98 – 6.91 (m, 4H), 4.11 (d, *J* = 9.6 Hz, 2H), 3.79 – 3.69 (m, 4H), 3.36 – 3.28 (m, 2H).
(1R*,5S*,6S*,7S*) Diastereomer (minor product): ^{1}H NMR (300 MHz, $CDCl_3$) δ 7.36 – 7.15 (m, 10H), 4.03 (d, *J* = 9.2 Hz, 1H), 3.87 – 3.76 (m, 2H), 3.66 (dd, *J* = 9.7, 6.6 Hz, 1H), 3.59 – 3.42 (m, 2H), 3.24 (dd, *J* = 15.6, 7.7 Hz, 1H), 3.17 – 3.06 (m, 1H).

(1S*,5R*,6S*)-6-phenyl-3-oxabicyclo[3.2.0]heptane (**2b**)[5]

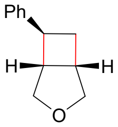

Was synthesized according to general procedure from **1b**. Reaction time – 16 hours. Eluent PE-EtOAc (40:1).Oil, 55.8 mg (d.r. 7:1) (Average yield of two runs – 50%).

(1S*,5R*,6S*)-Diastereomer (major product): ^{1}H NMR (300 MHz, Chloroform-*d*) δ 7.39 – 7.16 (m, 5H), 4.05 – 3.94 (m, 2H), 3.66 – 3.58 (m, 1H), 3.56 – 3.48 (m, 1H), 3.31 – 3.16 (m, 1H), 3.08 – 2.89 (m, 2H), 2.37 – 2.24 (m, 1H), 2.23 – 2.11 (m, 1H).

(1R*,5R*,7R*)-6,6-dimethyl-7-phenyl-3-oxabicyclo[3.2.0]heptane (**2c**)[5]

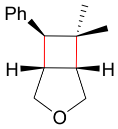

Was synthesized according to general procedure from **1c**. Reaction time – 6 hours. Eluent PE-EtOAc (40:1). Oil, 68.9 mg (d.r. > 10:1) (Average yield of two runs – 53%).

(1R*,5R*,7R*)-Diastereomer (major product) ^{1}H NMR (300 MHz, Chloroform-*d*) δ 7.35 – 7.27 (m, 2H), 7.24 – 7.11 (m, 3H), 4.17 (d, *J* = 10.0 Hz, 1H), 3.79 (d, *J* = 9.0 Hz, 1H), 3.56 – 3.41 (m, 2H), 3.33 – 3.23 (m, 1H), 2.99 (d, *J* = 7.3 Hz, 1H), 2.45 – 2.36 (m, 1H), 1.11 (s, 3H), 0.74 (s, 3H).

(1S*,5R*,6S*)-6-phenyl-3-tosyl-3-azabicyclo[3.2.0]heptane (**2d**)[16]

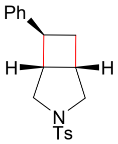

Was synthesized according to general procedure from **1d**. Reaction time – 1 hour. Eluent PE-EtOAc (8:1). White solid, 180 mg (d.r. 7:1) (Average yield of two runs – 87%).

(1S*,5R*,6S*) Diastereomer (major product): ^{1}H NMR (300 MHz, $CDCl_3$) δ 7.77 – 7.71 (m, 2H), 7.40 – 7.27 (m, 4H), 7.24 – 7.18 (m, 3H), 3.60 (d, *J* = 9.7 Hz, 1H), 3.55 (d, *J* = 9.6 Hz, 1H), 3.46 – 3.34 (m, 1H), 2.92 – 2.83 (m, 2H), 2.79 – 2.71 (m, 1H), 2.70 – 2.62 (m, 1H), 2.44 (s, 3H), 2.36 – 2.26 (m, 2H).

(1R*,5R*,7R*)-6,6-dimethyl-7-phenyl-3-tosyl-3-azabicyclo[3.2.0]heptane (**2e**)[5]

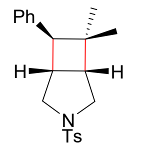

Was synthesized according to general procedure from **1e**. Reaction time – 1 hour. Eluent PE-EtOAc (10:1). White solid, 174 mg (d.r. > 10:1) (Average yield of two runs – 76%).

(1R*,5R*,7R*) Diasteromer (major product): ^{1}H NMR (300 MHz, $CDCl_3$) δ 7.73 (d, *J* = 8.2 Hz, 2H), 7.38 – 7.27 (m, 4H), 7.23 – 7.18 (m, 1H), 7.09 (d, *J* = 7.1 Hz, 2H), 3.72 (d, *J* = 10.6 Hz, 1H), 3.42 (d, *J* = 9.4 Hz, 1H), 3.24 – 3.10 (m, 2H), 2.70 – 2.56 (m, 2H), 2.45 (s, 3H), 2.28 (t, *J* = 7.5 Hz, 1H), 1.17 (s, 3H), 0.70 (s, 3H).

1,6,6-Trimethyl-7-phenyl-3-oxa-bicyclo[3.2.0]heptane (**2f**)[5]

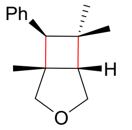

Was synthesized according to general procedure from **1f**. Reaction time – 17 hours. Eluent PE-EtOAc (40:1). Oil, 86.3 mg (d.r. 1.4:1) (Average yield of two runs – 62%)

(1R*,5S*,7R*) Diastereomer (major product)**:**^{1}H NMR (300 MHz, Chloroform-*d*) δ 7.31 – 7.27 (m, 4H), 7.25 – 7.17 (m, 1H), 4.11 (d, *J* = 9.8 Hz, 1H), 4.10 (d, *J* = 9.7 Hz, 1H), 3.66 (dd, *J* = 9.8, 5.6 Hz, 1H), 3.15 (d, *J* = 9.8 Hz, 1H), 3.10 (s, 1H), 2.18 (d, *J* = 5.6 Hz, 1H), 1.40 (s, 3H), 1.31 (s, 3H), 0.94 (s, 3H).
(1R*,5S*,7S*) Diastereomer (minor product): ^{1}H NMR (300 MHz, Chloroform-*d*) δ 7.32 – 7.24 (m, 4H), 7.24 – 7.17 (m, 1H), 4.14 (d, *J* = 10.1 Hz, 1H), 3.74 (d, *J* = 8.7 Hz, 1H), 3.61 (dd, *J* = 10.1, 6.5 Hz, 1H), 3.15 (s, 1H), 3.07 (d, *J* = 8.8 Hz, 1H), 2.01 (d, *J* = 6.5 Hz, 1H), 1.39 (s, 3H), 1.21 (s, 3H), 1.08 (s, 3H).

(1S*,2S*,5R*,6S*)-2-(4-Methoxy-phenyl)-6-phenyl-3-oxa-bicyclo[3.2.0]heptane (**2g**)[5]

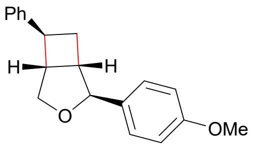

Was synthesized according to general procedure from **1g**. Reaction time – 3 hours. Eluent PE-EtOAc (40:1). Oil, 172 mg (d.r. 9:1) (Average yield of two runs – 96%). **(1S*,2S*,5R*,6S*) Diastereomer** ^{1}H NMR (300 MHz, $CDCl_3$) δ 7.39 – 7.17 (m, 7H), 6.91 – 6.84 (m, 2H), 5.09 (s, 1H), 4.02 (d, *J* = 8.6 Hz, 1H), 3.94 – 3.86 (m, 1H), 3.80 (s, 3H), 3.47 – 3.35 (m, 1H), 3.24 – 3.05 (m, 2H), 2.44 (dd, *J* = 8.5, 6.3 Hz, 2H).

(1S*,5R*,7R*)-7-(furan-2-yl)-6,6-dimethyl-3-oxabicyclo[3.2.0]heptane (**2h**)[5]

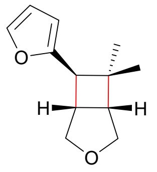

Was synthesized according to general procedure from **1h**. Reaction time – 1 hour. Eluent PE-EtOAc (40:1). Oil, 95 mg (d.r. 8:1) (Average yield of two runs – 77%)

(1S*,5R*,7R*) Diastereomer (major product): ^{1}H NMR (300 MHz, $CDCl_3$) δ 7.33 (dd, *J* = 1.8, 0.8 Hz, 1H), 6.31 (dd, *J* = 3.1, 1.9 Hz, 1H), 6.03 (dd, *J* = 3.2, 0.7 Hz, 1H), 4.11 (d, *J* = 10.1 Hz, 1H), 3.80 (d, *J* = 9.1 Hz, 1H), 3.51 – 3.35 (m, 2H), 3.24 – 3.10 (m, 1H), 2.86 (d, *J* = 7.0 Hz, 1H), 2.40 (t, *J* = 7.3 Hz, 1H), 1.06 (s, 3H), 0.88 (s, 3H).

(1R*,5R*,7R*)-6,6-dimethyl-7-(4-nitrophenyl)-3-oxabicyclo[3.2.0]heptane (**2i**)[5]

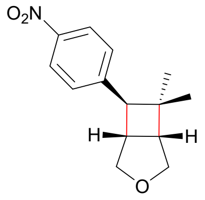

Was synthesized according to general procedure from **1i**. Reaction time – 1 hour. Eluent PE-EtOAc (7:1). White solid, 117.3 mg (d.r. > 10:1) (Average yield of two runs – 74%).

(1R*,5R*,7R*)-Diastereomer (major product): ^{1}H NMR (300 MHz, Chloroform-*d*) δ 8.21 – 8.12 (m, 2H), 7.35 – 7.26 (m, 2H), 4.19 (d, *J* = 10.2 Hz, 1H), 3.80 (d, *J* = 9.1 Hz, 1H), 3.57 – 3.41 (m, 2H), 3.30 (td, *J* = 7.7, 4.4 Hz, 1H), 3.09 (d, *J* = 7.3 Hz, 1H), 2.45 (t, *J* = 7.3 Hz, 1H), 1.15 (s, 3H), 0.74 (s, 3H).

(1R*,5R*,7S*)-7-(4-Chlorophenyl)-6,6-dimethyl-3-oxa-bicyclo[3.2.0]heptane (**2j**)[5]

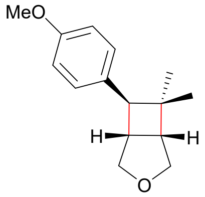

Was synthesized according to general procedure from **1j**. Reaction time – 3 hours. Eluent PE-EtOAc (40:1). Oil, 84.8 mg (d.r. > 10:1) (Average yield of two runs – 57%).

(1R*,5R*,7S*)-Diastereomer (major product): ^{1}H NMR (300 MHz, $CDCl_3$) δ 7.14 – 7.01 (m, 2H), 6.90 – 6.78 (m, 2H), 4.16 (d, *J* = 10.0 Hz, 1H), 3.80 (s, 3H), 3.78 (d, *J* = 10.1 Hz, 1H), 3.50 (dd, *J* = 10.0, 6.7 Hz, 1H), 3.43 (dd, *J* = 9.0, 4.5 Hz, 1H), 3.27 – 3.17 (m, 1H), 2.92 (d, *J* = 7.4 Hz, 1H), 2.39 (t, *J* = 7.4 Hz, 1H), 1.08 (s, 3H), 0.74 (s, 3H).

General procedure for photocatalytic cycloaddition of indoles

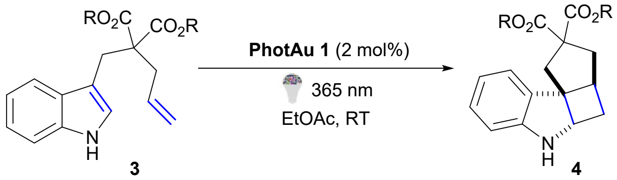

Indole **3** (0.2 mmol) and 2 mol% of [Au(SIPr)(cbz)] were weighted and transferred in 10 mL Shclenck tube equipped with stirring bar and septum. The tube was filled with Ar or N_2 using 3 vacuum-inert gas cycles. Under the flow of inert gas EtOAc (4 mL) was added in reaction mixture and the tube was closed with septum. Three freeze-thaw cycles were performed to degas the reaction mixture. The tube was placed into photoreactor for indicated time. The aliquots of reaction mixture were collected in the dark under flow of inert gas and evaporated. The ^{1}H NMR of aliquots were recorded to follow the reaction. After full conversion was reached, the solvent was evaporated and crudes of two identical reactions were combined. Product was purified using column chromatography with EtOAc-PE mixture as eluent.

Dimethyl (3aS*,4aR*,9bS*)-4a-phenyl-3a,4,4a,5-tetrahydro-1H-cyclopenta[2,3]cyclobuta[1,2-b]indole-2,2(3H)-dicarboxylate (**4a**)[11]

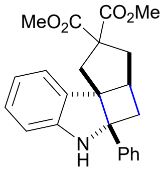

Was prepared according to general procedure from **3a**, using 2 mol% of [Au(SIPr)(Cbz)] (0.004 mmol, 3 mg). Reaction time – 1 hour. Eluent PE-EtOAc (7:1). Brown oil, average yield of two runs 112mg (81%).

^{1}H NMR (300 MHz, Chloroform-*d*) δ 7.39 – 7.14 (m, 5H), 7.12 – 7.00 (m, 2H), 6.75 (td, *J* = 7.4, 1.0 Hz, 1H), 6.63 – 6.55 (m, 1H), 4.21 (s, 1H), 3.74 (s, 3H), 3.36 (s, 3H), 3.05 – 2.92 (m, 1H), 2.88 – 2.76 (m, 2H), 2.65 – 2.53 (m, 2H), 2.51 – 2.34 (m, 2H).

Dimethyl (3aS*,4aS*,9bS*)-3a,4,4a,5-tetrahydro-1H-cyclopenta[2,3]cyclobuta[1,2-b]indole-2,2(3H)-dicarboxylate (**4b**)

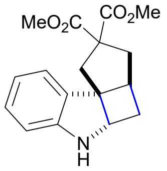

Was prepared according to general procedure from **3b**, using 2 mol% of [Au(SIPr)(Cbz)] (0.004 mmol, 3 mg). Reaction time – 1 hour. Eluent PE-EtOAc (5:1). Yellow oil, average yield of two runs 103.6 mg (86%).

^{1}H NMR (300 MHz, $CDCl_3$) δ 7.11 – 7.02 (m, 2H), 6.75 (td, *J* = 7.4, 1.0 Hz, 1H), 6.65 (d, *J* = 7.8 Hz, 1H), 4.04 (dd, *J* = 6.7, 4.7 Hz, 1H), 3.82 (s, 3H), 3.78 (s, 3H), 2.91 (ddd, *J* = 12.7, 8.4, 4.0 Hz, 1H), 2.81 (d, *J* = 14.3 Hz, 1H), 2.63 – 2.42 (m, 3H), 2.18 – 1.98 (m, 2H).
^{13}C NMR (75 MHz, $CDCl_3$) δ 172.8, 172.6, 152.4, 133.4, 128.3, 123.2, 119.3, 110.4, 63.4, 61.0, 60.9, 53.1, 52.9, 47.1, 43.8, 41.3, 35.3.
HRMS (ESI-TOF): Calcd for $C_{17}H_{20}NO_4^+$ $[M+H]^+$ 302.1387; found 302.1379.

Diethyl (3aS*,4aS*,9bS*)-3a,4,4a,5-tetrahydro-1H-cyclopenta[2,3]cyclobuta[1,2-b]indole-2,2(3H)-dicarboxylate (**4c**)

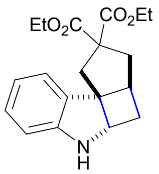

Was prepared according to general procedure from **3c**, using 2 mol% of [Au(SIPr)(Cbz)] (0.004 mmol, 3 mg). Reaction time – 1 hour. Eluent PE-EtOAc (4:1). Yellow oil, average yield of two runs 118.6 mg (90%).

^{1}H NMR (300 MHz, $CDCl_3$) δ 7.13 – 7.01 (m, 2H), 6.74 (td, *J* = 7.4, 0.9 Hz, 1H), 6.64 (d, *J* = 7.8 Hz, 1H), 4.34 – 4.18 (m, 4H), 4.07 (dd, *J* = 6.5, 4.8 Hz, 1H), 2.96 – 2.85 (m, 1H), 2.80 (d, *J* = 14.3 Hz, 1H), 2.63 – 2.40 (m, 3H), 2.18 – 2.00 (m, 2H), 1.35 – 1.23 (m, 6H).
^{13}C NMR (75 MHz, $CDCl_3$) δ 172.3, 172.2, 152.4, 133.6, 128.3, 123.2, 119.3, 110.4, 63.6, 61.9, 61.7, 61.0, 60.9, 47.2, 43.7, 41.2, 35.3, 14.2.
HRMS (ESI-TOF): Calcd for $C_{19}H_{24}NO_4^+$ $[M+H]^+$ 330.1700; found 330.1687.

Di-*tert*-butyl (3aS*,4aS*,9bS*)-3a,4,4a,5-tetrahydro-1H-cyclopenta[2,3]cyclobuta[1,2-b]indole-2,2(3H)-dicarboxylate (**4d**)

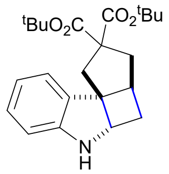

Was prepared according to general procedure from **3d**, using 2 mol% of [Au(SIPr)(Cbz)] (0.004 mmol, 3 mg). Reaction time – 1 hour. Eluent PE-EtOAc (5:1). Yellow oil, average yield of two runs 135.7 mg (88%).

^{1}H NMR (300 MHz, $CDCl_3$) δ 7.15 – 7.09 (m, 1H), 7.04 (td, *J* = 7.6, 1.3 Hz, 1H), 6.74 (td, *J* = 7.4, 1.0 Hz, 1H), 6.64 (d, *J* = 7.8 Hz, 1H), 4.15 – 4.06 (m, 1H), 2.94 – 2.81 (m, 1H), 2.70 (d, *J* = 14.3 Hz, 1H), 2.53 – 2.29 (m, 3H), 2.14 – 2.07 (m, 2H), 1.51 (s, 9H), 1.48 (s, 9H).
^{13}C NMR (75 MHz, $CDCl_3$) δ 171.6, 171.3, 152.4, 133.9, 128.2, 123.2, 119.2, 110.4, 81.7, 81.5, 65.0, 61.0, 60.9, 47.3, 43.5, 41.0, 35.4, 28.1.
HRMS (ESI-TOF): Calcd for $C_{23}H_{32}NO_4^+$ [M+H]$^+$ 386.2326; found 386.2316.

Dimethyl (3aS*,4aS*,9bS*)-7-fluoro-3a,4,4a,5-tetrahydro-1H-cyclopenta[2,3]cyclobuta[1,2-b]indole-2,2(3H)-dicarboxylate (**4e**)

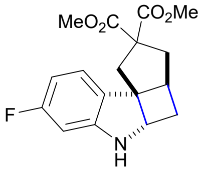

Was prepared according to general procedure from **3e**, using 2 mol% of [Au(SIPr)(Cbz)] (0.004 mmol, 3 mg). Reaction time – 1 hour. Eluent PE-EtOAc (6:1). Yellow oil, average yield of two runs 120 mg (94%).

^{1}H NMR (300 MHz, $CDCl_3$) δ 6.97 (dd, *J* = 8.2, 5.7 Hz, 1H), 6.37 (ddd, *J* = 9.4, 8.2, 2.3 Hz, 1H), 6.28 (dd, *J* = 10.0, 2.3 Hz, 1H), 4.12 – 4.05 (m, 1H), 3.81 (s, 3H), 3.77 (s, 3H), 2.92 – 2.82 (m, 1H), 2.78 (d, *J* = 14.3 Hz, 1H), 2.63 – 2.40 (m, 3H), 2.18 – 1.96 (m, 2H).
^{13}C NMR (75 MHz, $CDCl_3$) δ 172.7, 172.5, 163.8 (d, *J* = 242.0 Hz), 153.9 (d, *J* = 11.9 Hz), 128.6 (d, *J* = 2.1 Hz), 123.6 (d, *J* = 10.8 Hz), 105.0 (d, *J* = 22.9 Hz), 97.4 (d, *J* = 26.1 Hz), 63.3, 61.7, 60.0, 53.1, 52.9, 47.1, 43.8, 41.2, 35.3.
^{19}F NMR (377 MHz, $CDCl_3$) δ -115.2
HRMS (ESI-TOF): Calcd for $C_{17}H_{19}FNO_4^+$ [M+H]$^+$ 320.1293; found 320.1286.

(3aS*,4aS*,9bS*)-2,3,3a,4,4a,5-hexahydro-1H-cyclopenta[2,3]cyclobuta[1,2-b]indole (**4f**)

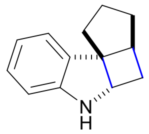

Was prepared according to general procedure from **3f**, using 2 mol% of [Au(SIPr)(Cbz)] (0.004 mmol, 3 mg). Reaction time – 1 hour. Eluent PE-EtOAc (6:1). White solid, average yield of two runs 57.7 mg (78%).

^{1}H NMR (300 MHz, $CDCl_3$) δ 7.06 – 6.95 (m, 2H), 6.71 (td, *J* = 7.4, 1.0 Hz, 1H), 6.62 (d, *J* = 7.8 Hz, 1H), 3.99 (br s, 1H), 3.91 (dd, *J* = 6.9, 3.9 Hz, 1H), 2.84 – 2.72 (m, 1H), 2.24 – 2.12 (m, 1H), 2.05 – 1.71 (m, 6H), 1.60 – 1.49 (m, 1H).
^{13}C NMR (75 MHz, $CDCl_3$) δ 153.2, 134.6, 127.7, 122.6, 118.7, 109.6, 60.6, 60.1, 46.7, 36.1, 36.0, 33.3, 26.2.
HRMS (ESI-TOF): Calcd for $C_{13}H_{16}N^+$ [M+H]$^+$ 186.1279; found 186.1270.

Diethyl (3aS*,4aS*,9bS*)-3a-methyl-3a,4,4a,5-tetrahydro-1H-cyclopenta[2,3]cyclobuta[1,2-b]indole-2,2(3H)-dicarboxylate (**4g**)

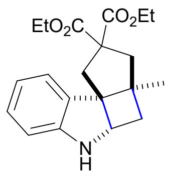

Was prepared according to general procedure from **3g**, using 2 mol% of [Au(SIPr)(Cbz)] (0.004 mmol, 3 mg). Reaction time – 5 hours. Eluent PE-EtOAc (5:1). Brown oil, average yield of two runs 118.2 mg (86%).

^{1}H NMR (300 MHz, $CDCl_3$) δ 7.08 – 6.97 (m, 2H), 6.74 (td, *J* = 7.4, 0.9 Hz, 1H), 6.63 (d, *J* = 7.8 Hz, 1H), 4.33 – 4.16 (m, 4H), 3.95 (dd, *J* = 6.9, 4.0 Hz, 1H), 2.84 (d, *J* = 14.2 Hz, 1H), 2.66 (d, *J* = 14.0 Hz, 1H), 2.55 (d, *J* = 14.2 Hz, 1H), 2.28 (dd, *J* = 13.1, 6.9 Hz, 1H), 2.18 (d, *J* = 14.0 Hz, 1H), 1.75 – 1.63 (m, 1H), 1.35 – 1.24 (m, 6H), 0.92 (s, 3H).
^{13}C NMR (75 MHz, $CDCl_3$) δ 172.3, 172.3, 153.3, 130.5, 128.3, 124.5, 118.8, 110.2, 63.3, 61.9, 61.7, 61.65, 58.2, 50.3, 48.0, 43.0, 25.3, 14.2 (d, *J* = 1.3 Hz).
HRMS (ESI-TOF): Calcd for $C_{20}H_{26}NO_4^+$ [M+H]$^+$ 344.1856; found 344.1853.

Dimethyl (3aS,4aS,9bS)-9-methoxy-3a,4,4a,5-tetrahydro-1H-cyclopenta[2,3]cyclobuta[1,2-b]indole-2,2(3H)-dicarboxylate (**4k**)

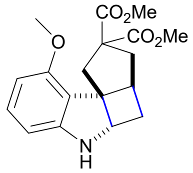

Was prepared according to general procedure from **3k**, using 2 mol% of [Au(SIPr)(Cbz)] (0.004 mmol, 3 mg). Reaction time – 1 hour. Eluent PE-EtOAc (4:1). Yellow oil, average yield of two runs 120 mg (91%).

^{1}H NMR (300 MHz, $CDCl_3$) δ 7.03 (t, *J* = 8.0 Hz, 1H), 6.37 – 6.28 (m, *J* = 11.6, 4.3 Hz, 2H), 3.99 (dd, *J* = 6.8, 5.1 Hz, 1H), 3.81 (s, 3H), 3.77 (s, 3H), 3.76 (s, 3H), 3.06 (d, *J* = 14.1 Hz, 1H), 3.07 – 2.96 (m, 1H), 2.73 – 2.58 (m, 2H), 2.40 (dd, *J* = 13.9, 3.0 Hz, 1H), 2.21 – 1.98 (m, 2H).

^{13}C NMR (75 MHz, $CDCl_3$) δ 173.25, 172.6, 157.5, 153.25, 129.8, 118.9, 104.8, 102.6, 63.6, 60.9, 60.6, 55.3, 52.9, 52.8, 46.0, 41.1, 40.75, 35.05.

Functionalization of indole product

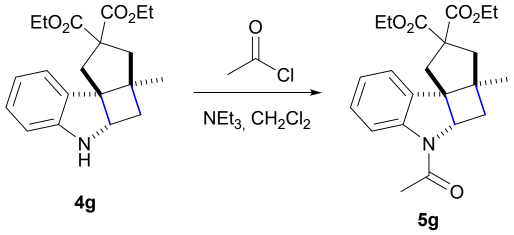

Indole **4g** (0.22 mmol, 75mg) and triethylamine (3 eq., 0.66 mmol, 90.7 µL) were dissolved in CH_2Cl_2 (2 mL). Solution of acetyl chloride (1.5 eq., 0.33 mmol, 23.2 µL) in CH_2Cl_2 (1 mL) was added dropwise to indole at 0°C. Stirring was continued at room temperature until full conversion, which was monitored with TLC. Water (10 mL) was added to reaction mixture, then the crude was extracted with CH_2Cl_2 (3x5mL). The reaction crude was then purified using column chromatography and EtOAc:Petroleum ether mixture (1:2) as eluent. Product **5g** was obtained as white solid (70 mg, 83%).

^{1}H NMR (300 MHz, $CDCl_3$) δ 8.26 (d, *J* = 8.1 Hz, 1H), 7.29 – 7.19 (m, 1H), 7.14 – 7.02 (m, 2H), 4.37 – 4.27 (m, 3H), 4.23 (q, *J* = 7.1 Hz, 2H), 2.85 (d, *J* = 14.4 Hz, 1H), 2.69 (d, *J* = 14.2 Hz, 1H), 2.59 (d, *J* = 14.4 Hz, 1H), 2.49 (dd, *J* = 13.4, 7.0 Hz, 1H), 2.22 (d, *J* = 14.2 Hz, 1H), 2.05 (d, *J* = 6.0 Hz, 3H), 1.82 (dd, *J* = 13.4, 3.8 Hz, 1H), 1.34 (t, *J* = 7.1 Hz, 3H), 1.28 (t, *J* = 7.1 Hz, 3H), 0.87 (s, 3H).
^{13}C NMR (75 MHz, $CDCl_3$) δ 172.1, 171.7, 168.6, 145.0, 132.1, 128.7, 124.0, 123.8, 117.4, 62.1, 62.0, 61.5, 60.3, 60.0, 49.1, 47.6, 42.8, 40.4, 25.2, 24.0, 14.3, 14.2.

Mechanistic studies

Absorption studies

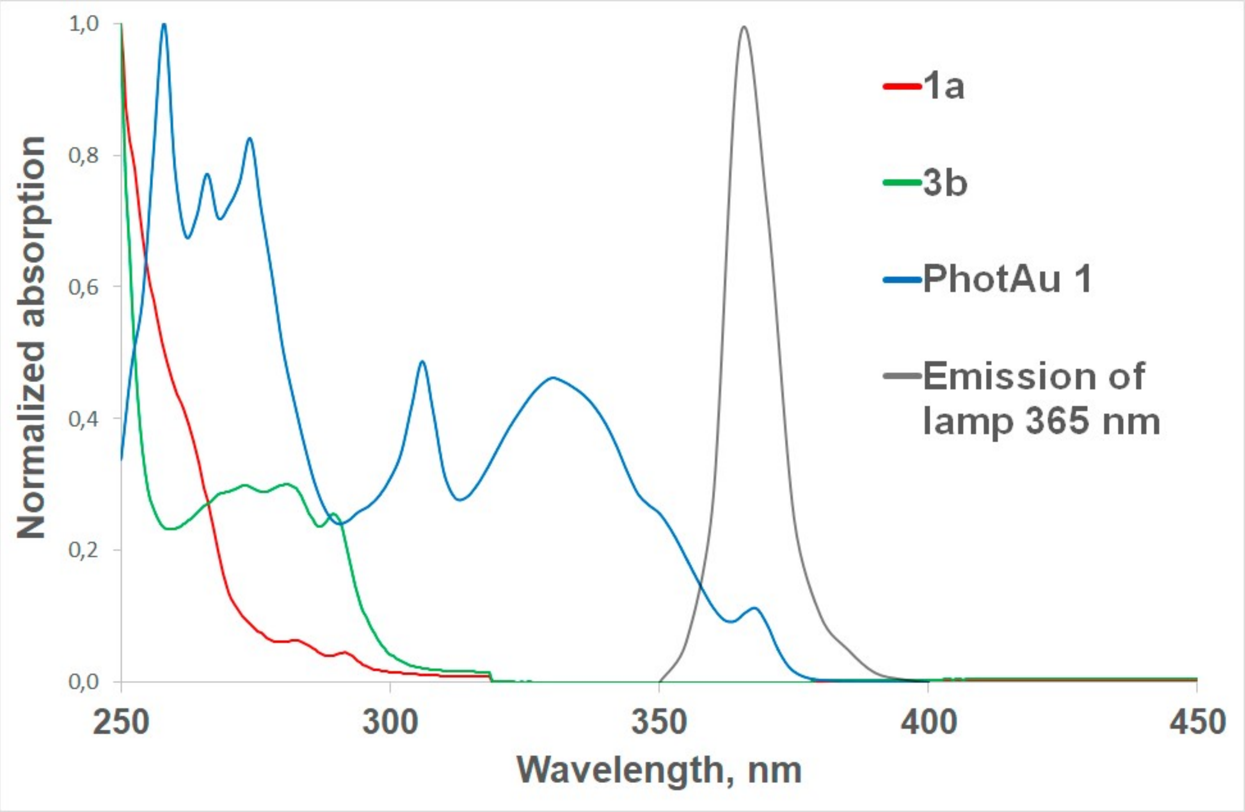

Figure S4. Absorption spectra of **1a**, **3b** and [Au(SIPr)(Cbz)].

Conclusion: The only component of the reaction mixture that absorbs light from the lamp is photocatalyst

Electrochemical studies

Procedure for electrochemical studies

Experiments were conducted inside a glovebox under an argon atmosphere. A stock solution of 0.1 M supporting electrolyte NBu_4PF_6 in dichloromethane (solvent purified using a J.C. Meyer Phoenix Solvent Purification System in which solvents are dried over aluminium oxide using argon as carrier gas) was made. The complex was weighed in a 30 mL vial and 10 mL of the stock solution of electrolyte was added to result in a solution containing 1 mM of analyte. After addition of the solvent, the vial was stirred momentarily to assure proper mixing. The screen-printed electrode is then submerged in the solution and the voltammogram is recorded, starting at 0 V, going to positive potentials first.

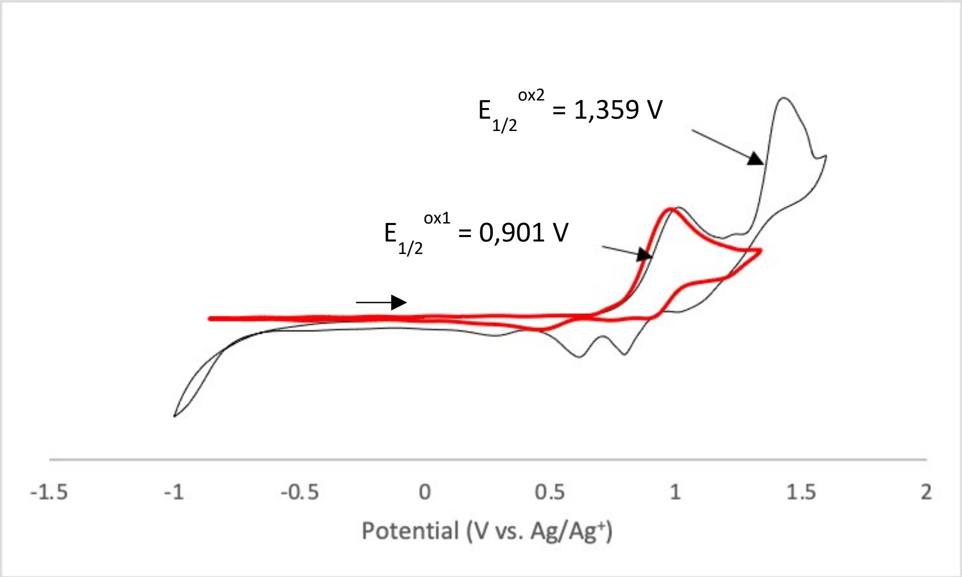

Figure S5. Cyclic voltammogram of [Au(SIPr)(Cbz)] (black). Partial cyclic voltammogram of [Au(SIPr)(Cbz)] stopped after first oxidation (red). Conditions: 25°C, 5 mM of analyte, 0.1 M NBu_4PF_6 in dichloromethane, Pt work and auxiliary electrode, scan rate 100 mVs^{-1}.

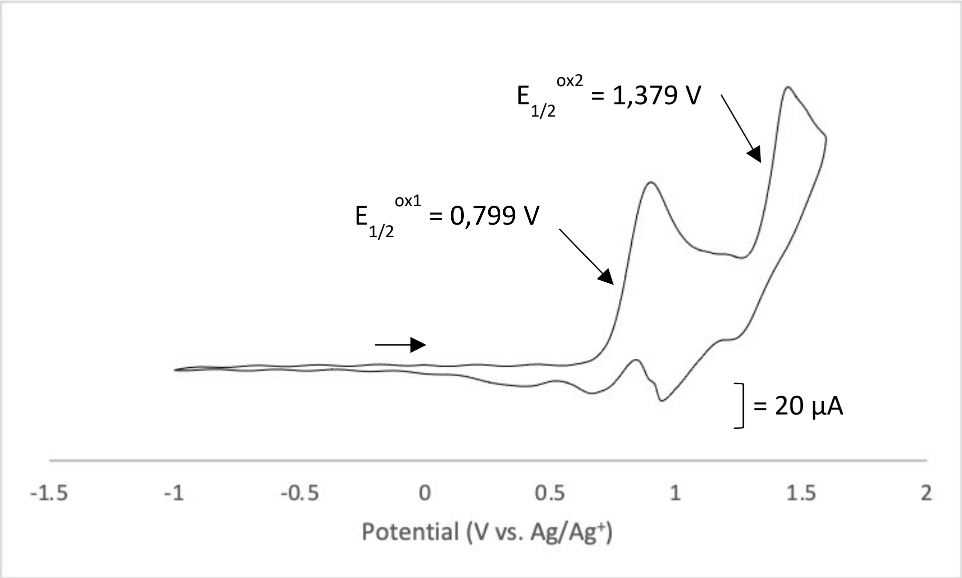

Figure S6. Cyclic voltammogram of [Au(IPr)(Cbz)]. Conditions: 25°C, 5 mM of analyte, 0.1 M NBu_4PF_6 in dichloromethane, Pt work and auxiliary electrode, scan rate 100 mVs^{-1}.

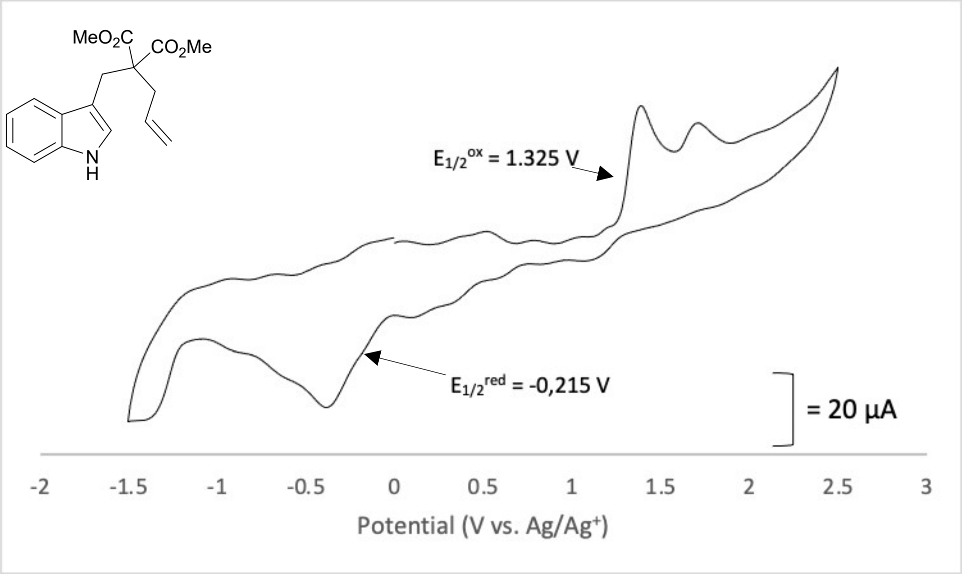

Figure S7. Cyclic voltammogram of **3b**

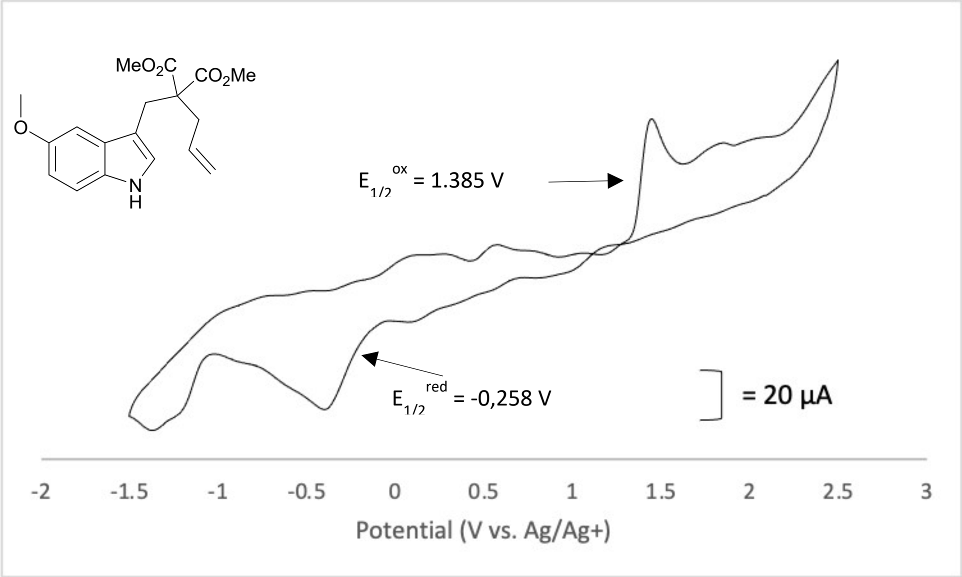

Figure S8. Cyclic voltammogram of **3i**

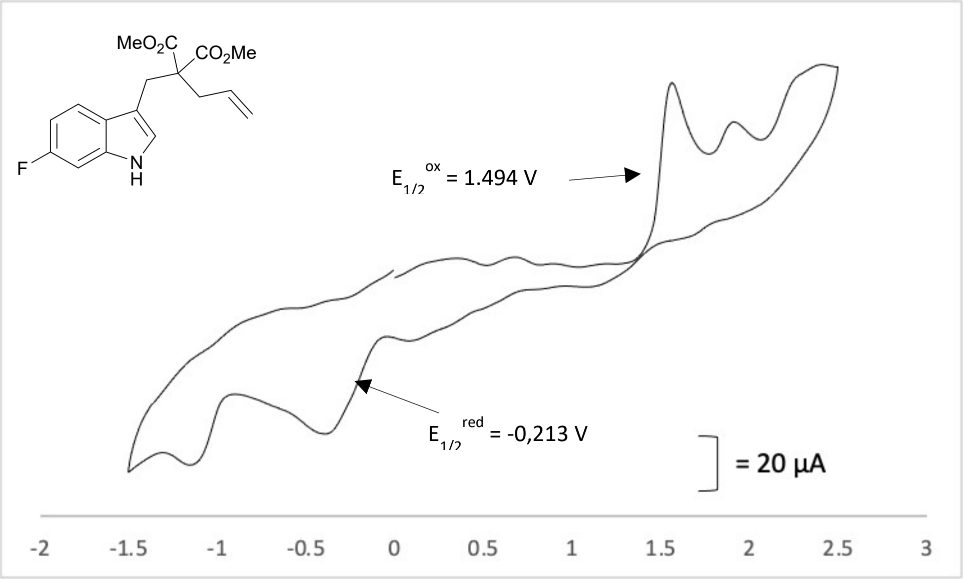

Figure S9. Cyclic voltammogram of **3e**

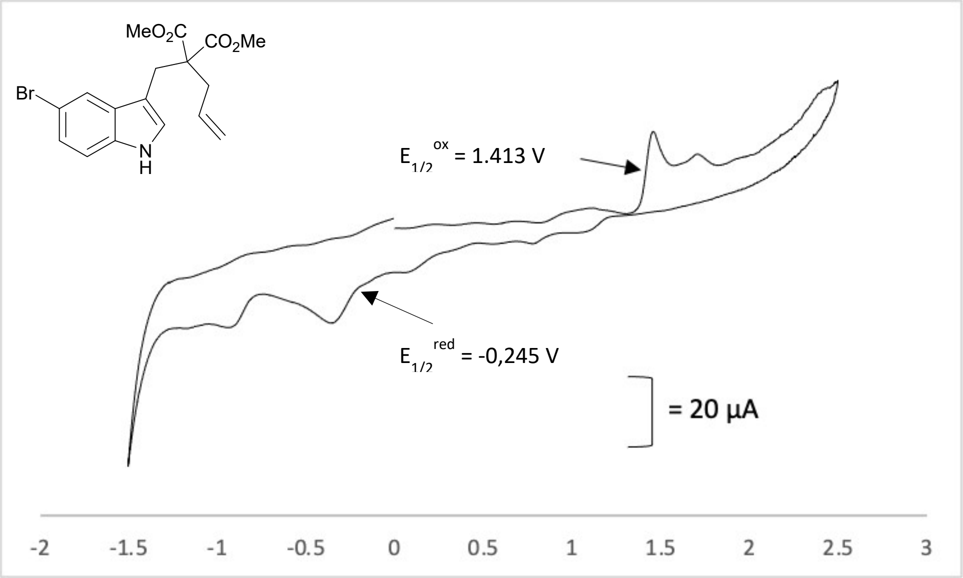

Figure S10. Cyclic voltammogram of **3j**

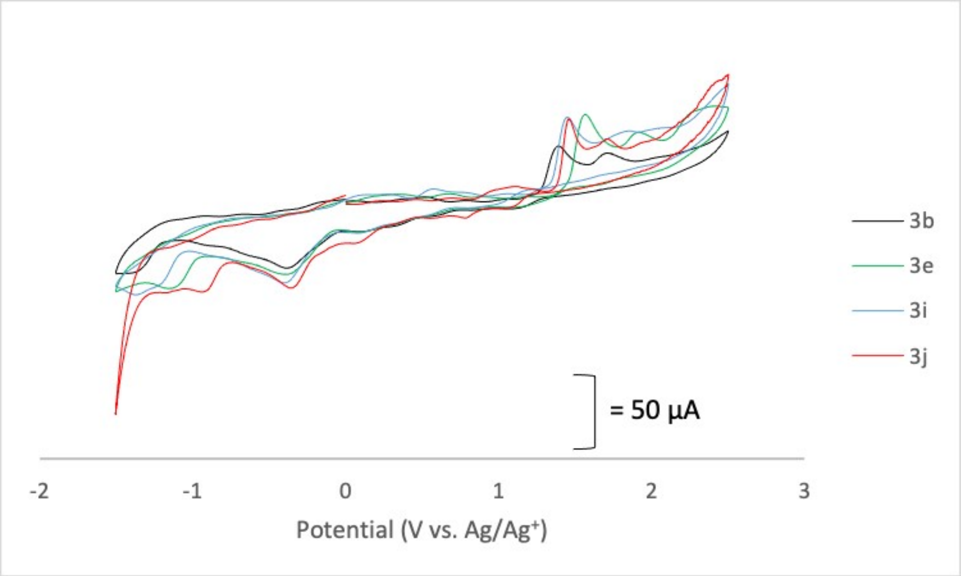

Figure S11. Cyclic voltammograms of **3b**, **3e**, **3i** and **3j**

Conclusion: Redox potentials of **3b** and **3e,** which reacted in intramolecular [2+2] cycloaddition under optimized conditions, and **3i**, **3j** which did not react under optimized conditions, did not differ significantly. Therefore, the reason of the lack of reactivity of **3i** and **3j** is not different Redox potentials and thus the mechanism unlikely involves electron transfer.

On/Off experiment

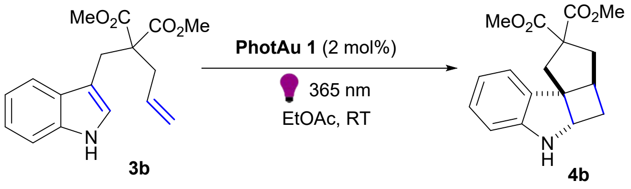

Indole **3b** (0.1 mmol, 30.1 mg) and [Au(SIPr)(Cbz)] (2 mol%, 0.002 mmol, 1.5 mg) were weighted and transferred in 10 mL Schlenk tube equipped with stirring bar and septum. The tube was filled with Ar or N_2 using 3 vacuum-inert gas cycles. Under the flow of inert gas EtOAc (2 mL) was added in reaction mixture and the tube was closed with septum. Three freeze-thaw cycles were performed to degas the reaction mixture. During “On” period the Schlenk tube was placed in photoreactor, during “Off” period the lamp was turned off and the tube was covered with aluminum foil without the interruption of stirring. After each period, the aliquot of reaction mixture was taken under the flow of Ar and analyzed with ^{1}H NMR.

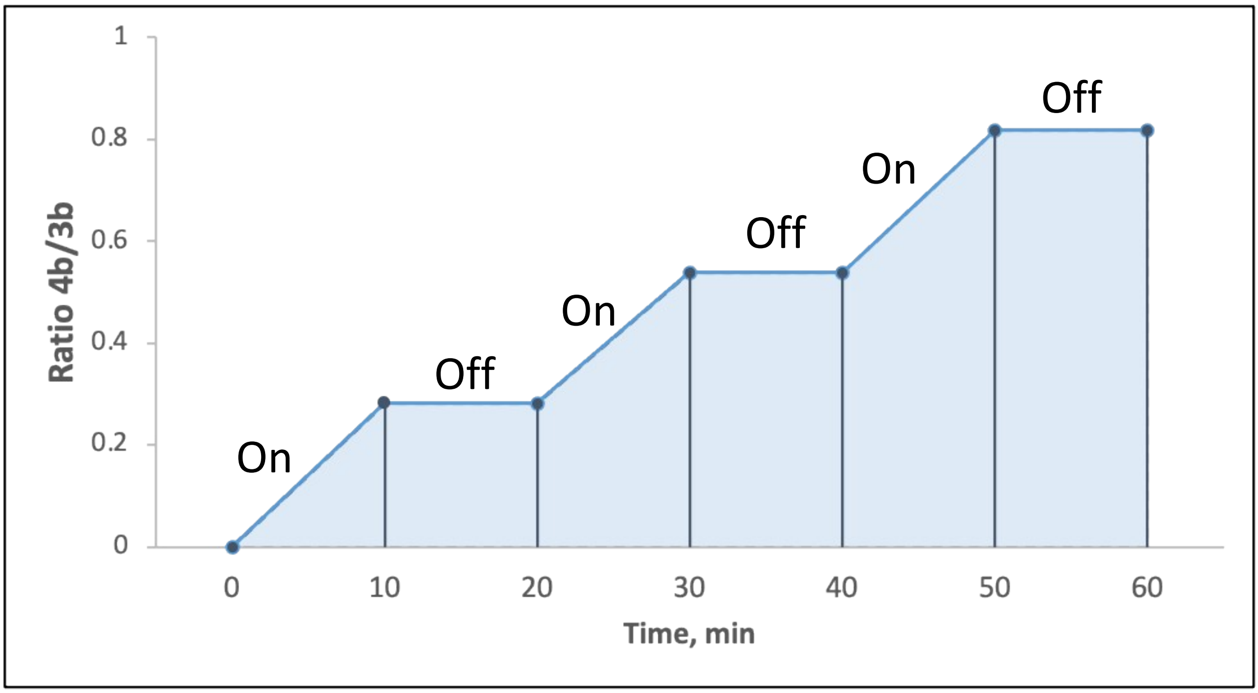

Figure S12. On/Off experiment

Quenching studies and Stern-Volmer plot

Quenching studies were performed using 4·10^{-5} M solution of **PhotAu 1** in THF and varying concentration of indole **3b.** The samples were prepared at once in argon-filled glovebox using degassed inhibitor-free THF in screw-cap quartz cuvettes. All samples were excited at λ_{ex} = 369 nm and emission was detected at λ_{em} = 429 nm.

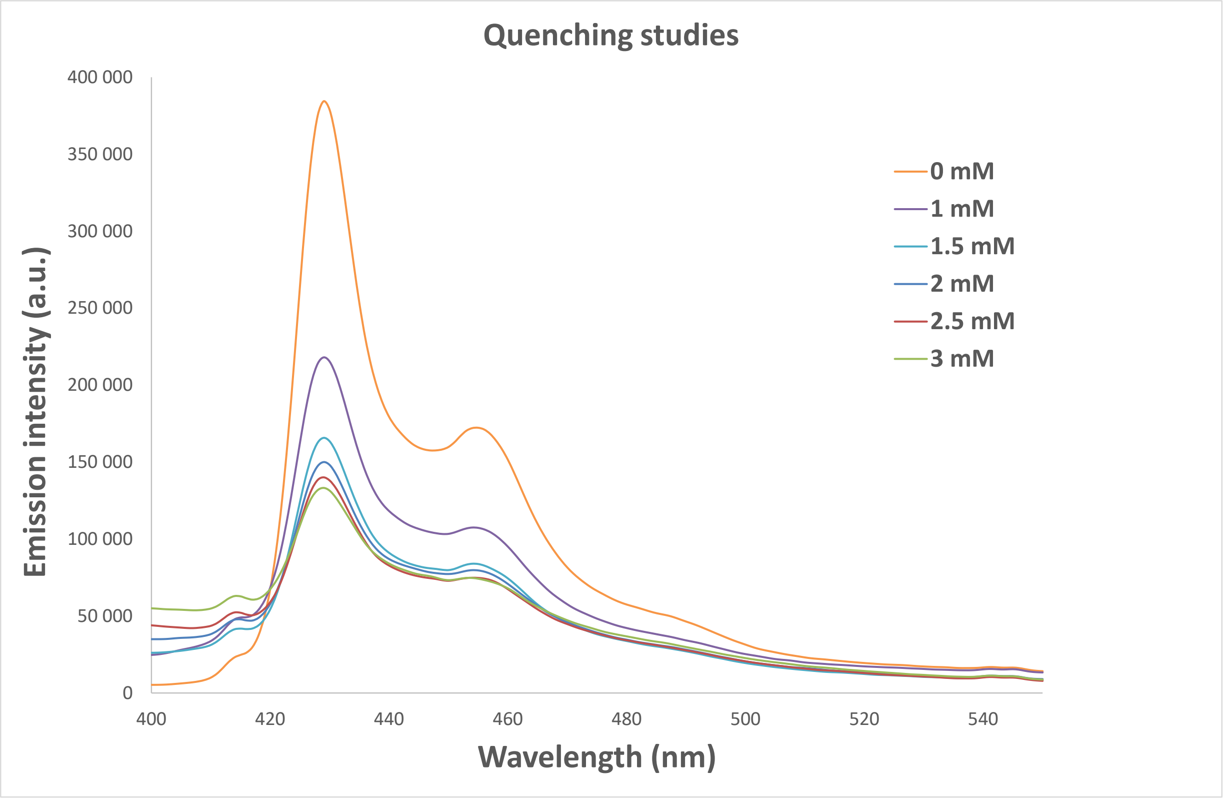

Figure S13. Luminescence spectra of 4·10^{-5} M solution of **PhotAu 1** in THF with varying concentrations of indole **3b**

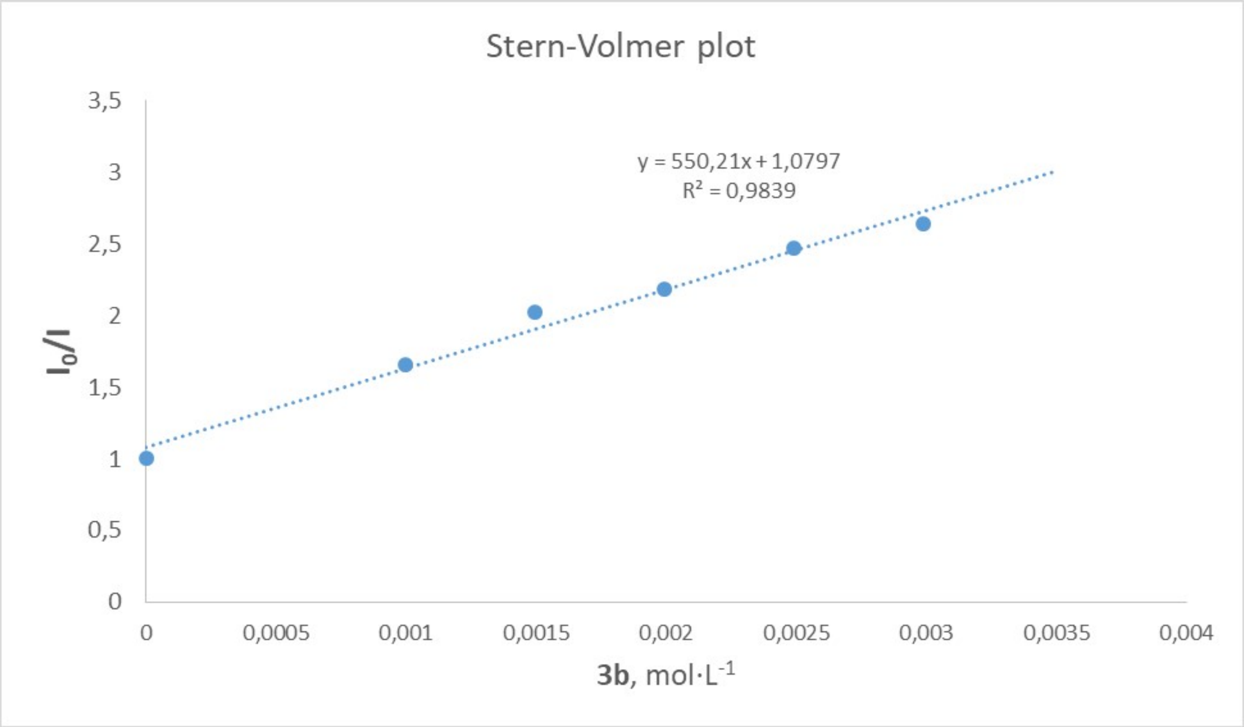

Figure S14. Stern-Volmer plot for quenching of excited state **PhotAu 1** in the presence of **3b**

The Stern-Volmer quenching constant K_V was determined to be $K_V = 550.21$ L·mol^{-1}. Assuming that there is no static quenching, the quenching rate constant $\mathbf{k_q}$ can be determined using the known intrinsic lifetime of **PhotAu 1** (τ = 266 μs) from equation:

$$k_q = \frac{K_V}{\tau}$$

Therefore the quenching rate constant $\mathbf{k_q}$ was determined to be $\mathbf{k_q} = 2.07 \cdot 10^6$ M^{-1}·s^{-1}

The quantum yield of the reaction with indole 3b

To determine the reaction quantum yield of the intramolecular [2+2]-cycloaddition of **3b** the photon flux in our photocatalytic setup was determined using ferrioxalate actinometry.[17]

Determination of photon flux

A ferrioxalate solution (0.150 M) was prepared by dissolving potassium ferrioxalate hydrate (737 mg) in aq. H_2SO_4 (0.05 M, 10.0 mL). The solution of 1,10-phenanthroline (40.0 mg), NaOAc (4.50 g) in aq. H_2SO_4 (0.5 M, 20.0 mL) was prepared for complexation. Both solutions were prepared in the dark, covered with foil and stored in the dark.

2.0 mL of ferrioxalate solution was transfered to Schlenk tube covered with foil and equipped with stirring bar. The Schlenck tube as placed in the photoreactor and solution was irradiated for 60 seconds. Afterwards, the aliquot (0.100 mL) was transferred to a vial covered with foil and equipped with stirring bar. 2.0 mL of phenanthroline solution was added to the vial and the mixure was stirred for 1 hour in the dark at RT for complexation of formed Fe^{2+}. Then the solution was diluted with 8.0 mL of water and 3.0 mL of the resulting solution was transferred to quartz cuvette and the absorbtion of the solution was

measured at 510 nm. This experiment was repeated twice. A control experiment was prepared accordingly, but without the irradiation of the solution in Schlenck tube.

The amount of $[Fe(phen)_3]^{2+}$ can then be calculated from the absorption difference between the irradiated and non-irradiated sample ΔA as:

$$n(Fe^{2+}) = \frac{\Delta A\,(510\,\mathrm{nm}) \cdot V_1 \cdot V_3}{d \cdot \varepsilon_{510}([Fe(phen)_3]^{2+}) \cdot V_2}$$

Where d is the path length (1 cm), ε ($[Fe(phen)_3]^{2+}$) is the molar absortivity of the phenanthroline complex at 510 nm (11100 $L \cdot mol^{-1} \cdot cm^{-1}$), V_1 is the the volume of the irradiated solution (0.002 L), V_2 is the volume of the aliquot taken for complexation (0.0001 L) and V_3 is the volume of the solution after complexation (0.0101 L).

Solution	#1	#2	Control
$A_{(510\,nm)}$	1.5464	1.5451	0.1232

The $n(Fe^{2+})$ was determined to be $2.59 \cdot 10^{-5}$ mol. From this value the photon flux Φ can be determined as

$$\Phi = \frac{n(Fe^{2+})}{\phi_{Act} \cdot t \cdot f}$$

Where ϕ_{Act} is the reported quantum yield for ferrioxalate actinometer at 365 nm (1.26), t is the time of irradiation (60 s) and f is the fraction of light absorbed at the wavelength of irradiation, which can be determined as

$$f = 1 - 10^{-A(365nm)}$$

The absorbance of ferrioxalate solution $A_{(365\,nm)}$ was > 3, so that f can be assumed as 1.

Therefore from these experimental data the photon flux in our system was calculated to be $3.43 \cdot 10^{-7}$ $mol \cdot s^{-1}$

Determination of the quantum yield of the reaction

Indole **3b** (0.1 mmol, 30.1 mg) and [Au(SIPr)(Cbz)] (2 mol%, 0.002 mmol, 1.5 mg) were weighted and transferred in 10 mL Schlenk tube equipped with stirring bar and septum. The tube was filled with Ar using 3 vacuum-inert gas cycles. Under the flow of inert gas EtOAc (2.0 mL) was added in reaction mixture and the tube was closed with septum. Three freeze-thaw cycles were performed to degas the reaction mixture. The Schlenck tube was placed in the photoreactor and the reaction mixture was irradiated for 600 seconds. Then the aliquot of the reaction mixture was analyzed using ^1H NMR and the yield was determined using 1,3,5-trimethoxybenzene as an internal standard. A total amount of *n*(**4b)** = 0.022 mmol (22%) was observed.

Therefore, the reaction quantum yield ϕ_R can be determined following the equation:

$$\phi_R = \frac{n(prod)}{\Phi \cdot t \cdot f_R}$$

Where f_R is the fraction of light absorbed by the reaction mixture at 365 nm. It was calculated using the absorbance of reaction mixure at 365 nm $A_{R(365\ nm)}$ = 3.05, so f_R can be assumed as 1.

Using the detemined photon flux the reaction quantum yield was determined to be ϕ_R = 0.11. This value correlates with the proposed triplet-triplet energy transfer mechanism.

Reaction with triplet quencher

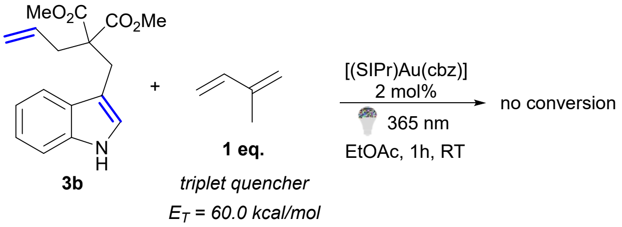

Indole **3b** (0.2 mmol, 60.2 mg) and [Au(SIPr)(cbz)] (2 mol%, 0.004 mmol, 3 mg) were weighted and transferred in 10 mL Schlenk tube equipped with stirring bar and septum. The tube was filled with Ar or N_2 using 3 vacuum-inert gas cycles. Under the flow of inert gas EtOAc (4 mL) and isoprene (1 eq., 0.2 mmol, 20 µL) were added in reaction mixture and the tube was closed with septum. Three freeze-thaw cycles were performed to degas the reaction mixture. The tube was placed in photoreactor for 1 hour. After that time, solvent was evaporated and ^1H NMR of reaction mixture was recorded. No traces of product were observed.

Conclusion: The reaction involves excited-state intermediates.

DFT calculations

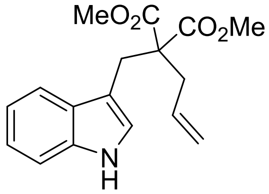

3b: 65.2 kcal/mol

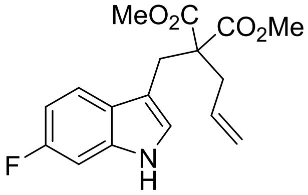

3e: 63.6 kcal/mol

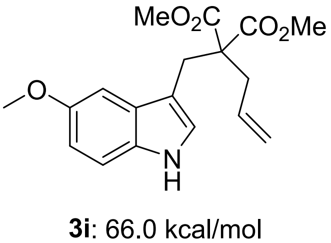

3i: 66.0 kcal/mol

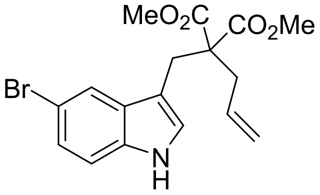

3j: 64.6 kcal/mol

Figure S15. Calculated E_T values for substrates **3**

Conclusion: The E_T value of **3j** is lower than E_T value of **3b**, which reacted in intramolecular 2+2 cycloaddition. Therefore, the lack of reactivity is not attributed to too high triplet value of substrate. And although the E_T value of **3i** is somewhat higher than one of **3b**, it does not exceed the E_T value of **[Au(SIPr)(Cbz)]**, therefore the energy transfer remain exergonic. Herein, taking into account the RedOx potentials and E_T values of reacting and non-reacting substrates, we believe that neither of these two factors is responsible for the lack of reactivity. We assume that possible too short lifetime of excited triplet states of **3i** and **3j** might be a cause.

Cartesian coordinates and energies and of all optimized structures

3b

```
Opt @ uB3LYP/6-311+G(2d,p) in Ethyl Acetate (SMD model)
SCF Done: E(UB3LYP) = -1015.22262888 a.u.
Zero-point correction = 0.331227 Hartree/Particle
Sum of electronic and thermal Free Energies = -1014.943841 a.u.
---------------------------------------------------------------
 N     0.000000     0.000000     0.000000
 C     0.000000     0.000000     1.374344
 C     1.357785     0.000000     1.791794
 C     2.172120    -0.001202     0.601462
 C     1.298131     0.004377    -0.456600
 C    -1.060730     0.004690     2.281151
 C    -0.750022     0.012811     3.632202
 C     0.587711     0.017536     4.070813
 C     1.638297     0.010731     3.167256
 C     3.673469     0.053314     0.558830
 C     4.451882    -1.284627     0.278637
 C     4.480435    -1.580364    -1.226586
 O     5.407790    -2.499807    -1.521927
 C     5.511175    -2.890690    -2.907442
 C     5.884269    -1.033027     0.769402
 O     6.527313    -0.179318    -0.039991
 C     7.873089     0.180173     0.341217
 C     3.807283    -2.465652     1.055304
 C     4.434064    -3.823461     0.915619
 C     3.755465    -4.923908     0.607973
 O     6.360746    -1.481221     1.784349
```

O	3.756830	-1.074426	-2.051092
H	1.507441	0.002913	-1.513252
H	-0.819433	0.008295	-0.587531
H	-2.090208	0.003484	1.941167
H	-1.550197	0.016822	4.363239
H	0.795584	0.025587	5.134644
H	2.662549	0.014456	3.522888
H	4.001757	0.780976	-0.183152
H	4.019420	0.422184	1.527882
H	8.215345	0.871773	-0.424687
H	7.875553	0.663488	1.317709
H	8.508491	-0.704864	0.365008
H	6.305465	-3.632462	-2.940428
H	4.572974	-3.325517	-3.250952
H	5.768497	-2.030666	-3.525322
H	3.831307	-2.179695	2.111281
H	2.757517	-2.521551	0.765201
H	5.496008	-3.896411	1.127985
H	4.240574	-5.893055	0.563835
H	2.691026	-4.898037	0.392909

3b(T)
Opt @ uB3LYP/6-311+G(2d,p) in Ethyl Acetate (SMD model)
SCF Done: E(UB3LYP) = -1015.11112616 a.u.
Zero-point correction = 0.325429 Hartree/Particle
Sum of electronic and thermal Free Energies = -1014.839881 a.u.

C	0.000000	0.000000	0.000000
C	0.000000	0.000000	1.443243
C	1.255432	0.000000	2.130202
C	2.470415	-0.002044	1.486305
C	2.435862	-0.005059	0.020180
C	1.250031	-0.002957	-0.669277
C	-1.015409	0.008489	2.394232
C	-0.381455	0.015907	3.708323
N	0.964309	0.006571	3.488397
C	-2.483166	0.076139	2.126153
C	-3.346267	-1.198373	2.459445
C	-2.565742	-2.490931	2.089989
C	-3.246206	-3.808501	2.328139
C	-2.700121	-4.808957	3.011301
C	-3.742502	-1.215101	3.941442
O	-3.200356	-0.579435	4.814022
C	-4.613830	-1.064021	1.602321
O	-4.862165	-1.721147	0.620926
O	-4.763756	-2.050692	4.158460
C	-5.207166	-2.186559	5.526102
O	-5.385529	-0.062099	2.044912
C	-6.590168	0.203979	1.293032
H	-0.816497	0.018857	4.689890
H	1.662164	0.012277	4.218207
H	3.411536	-0.000226	2.020085
H	3.375551	-0.008886	-0.519136
H	1.264887	-0.004962	-1.753556
H	-0.927355	-0.000356	-0.557299
H	-2.914000	0.916751	2.674754
H	-2.617870	0.285902	1.063379
H	-7.066902	1.040496	1.797940
H	-6.345179	0.469945	0.265191

```
 H    -7.242542    -0.668721     1.304249
 H    -6.031357    -2.894747     5.493665
 H    -4.400680    -2.571630     6.149262
 H    -5.546184    -1.225054     5.910658
 H    -2.326378    -2.406568     1.025305
 H    -1.618042    -2.474778     2.630561
 H    -4.220716    -3.945524     1.870633
 H    -3.208190    -5.760818     3.121847
 H    -1.724918    -4.716015     3.480571
```

3e

```
Opt @ uB3LYP/6-311+G(2d,p) in Ethyl Acetate (SMD model)
SCF Done: E(UB3LYP) = -1114.49354070 a.u.
Zero-point correction = 0.322991 Hartree/Particle
Sum of electronic and thermal Free Energies = -1114.223946 a.u.
----------------------------------------------------------
 C     0.000000     0.000000     0.000000
 C     0.000000     0.000000     1.402160
 C     1.246045     0.000000     2.085226
 C     2.471536    -0.005301     1.414630
 C     2.400306    -0.007673     0.040288
 C     1.205757    -0.003768    -0.683459
 C    -1.037036     0.011285     2.405871
 C    -0.393914     0.022982     3.616153
 N     0.972220     0.011276     3.427938
 C    -2.515616     0.069168     2.142899
 C    -3.340709    -1.262191     2.283495
 C    -2.558374    -2.457807     1.673374
 C    -3.208064    -3.811774     1.703426
 C    -2.610549    -4.909420     2.155344
 F     3.571572    -0.012674    -0.666203
 C    -3.674970    -1.529347     3.757114
 O    -3.135971    -1.002762     4.701446
 C    -4.643212    -1.017263     1.508665
 O    -4.905158    -1.480876     0.424992
 O    -4.640972    -2.448343     3.875106
 C    -5.025220    -2.812955     5.217868
 O    -5.433820    -0.151001     2.157845
 C    -6.670421     0.207340     1.503048
 H    -0.808954     0.030436     4.609910
 H     1.658293     0.018711     4.166931
 H     3.425023    -0.005487     1.927666
 H     1.242850    -0.004271    -1.765470
 H    -0.930509     0.003313    -0.555403
 H    -2.982535     0.810938     2.790489
 H    -2.655982     0.421393     1.117794
 H    -7.161547     0.906252     2.175626
 H    -6.468175     0.682095     0.543340
 H    -7.290101    -0.676702     1.354985
 H    -5.803014    -3.563886     5.102290
 H    -4.174575    -3.228645     5.757118
 H    -5.411859    -1.944007     5.749930
 H    -2.365294    -2.189610     0.630122
 H    -1.589920    -2.511614     2.172089
 H    -4.206360    -3.884963     1.283729
 H    -3.100013    -5.876501     2.114652
 H    -1.610562    -4.883272     2.578773
```

3e (T)

Opt @ uB3LYP/6-311+G(2d,p) in Ethyl Acetate (SMD model)
SCF Done: E(UB3LYP) = -1114.38449786 a.u.
Zero-point correction = 0.317475 Hartree/Particle
Sum of electronic and thermal Free Energies = -1114.122557 a.u.

N	0.000000	0.000000	0.000000
C	0.000000	0.000000	1.384010
C	1.369129	0.000000	1.798861
C	2.161840	0.000739	0.656387
C	1.267887	0.003025	-0.490871
C	-1.062960	0.004114	2.263518
C	-0.682549	0.007950	3.667621
C	0.593619	0.009903	4.135309
C	1.677230	0.006502	3.212893
F	-1.722105	0.008352	4.546866
C	3.654205	0.065209	0.609647
C	4.426003	-1.226551	0.146151
C	5.848233	-1.075461	0.707718
O	6.505447	-0.088844	0.083503
C	7.843443	0.190886	0.551836
C	3.741425	-2.499552	0.717593
C	4.352974	-3.830580	0.385657
C	3.668119	-4.852178	-0.117516
C	4.495930	-1.295139	-1.384915
O	5.448525	-2.142160	-1.788200
C	5.590038	-2.324060	-3.214035
O	3.777344	-0.685631	-2.141173
O	6.303006	-1.708918	1.628833
H	1.487769	-0.000293	-1.542168
H	-0.834677	0.001355	-0.568931
H	-2.102740	0.008982	1.970377
H	0.769936	0.011767	5.203715
H	2.698943	0.006600	3.565143
H	3.962301	0.884786	-0.042816
H	4.006356	0.308110	1.613869
H	8.198491	1.013929	-0.063337
H	7.824963	0.481322	1.601809
H	8.479839	-0.683857	0.420765
H	6.403721	-3.034775	-3.335086
H	4.669925	-2.724973	-3.637831
H	5.837245	-1.377040	-3.692908
H	3.735352	-2.377256	1.805352
H	2.699879	-2.493656	0.392704
H	5.404797	-3.958286	0.619995
H	4.138694	-5.812202	-0.299957
H	2.612764	-4.769168	-0.360884

3i
Opt @ uB3LYP/6-311+G(2d,p) in Ethyl Acetate (SMD model)
SCF Done: E(UB3LYP) = -1129.78136985 a.u.
Zero-point correction = 0.363385 Hartree/Particle
Sum of electronic and thermal Free Energies = -1129.473672 a.u.

N	0.000000	0.000000	0.000000
C	0.000000	0.000000	1.377867
C	1.350835	0.000000	1.799019
C	2.167979	-0.001969	0.612553
C	1.296268	0.003245	-0.451274
C	-1.055214	0.006149	2.291492

C	-0.747054	0.015510	3.638110
C	0.595048	0.019797	4.080591
C	1.646526	0.011446	3.176752
O	0.746655	0.031646	5.443049
C	2.069353	0.026772	5.968178
C	3.669084	0.053424	0.572119
C	4.451255	-1.288142	0.316304
C	4.487048	-1.607259	-1.184061
O	5.412275	-2.535280	-1.459866
C	5.519572	-2.949585	-2.838160
C	5.880418	-1.026663	0.810215
O	6.530218	-0.192688	-0.014148
C	7.872722	0.176206	0.369388
C	3.804244	-2.457580	1.108175
C	4.431661	-3.817293	0.992446
C	3.754113	-4.921628	0.696589
O	6.349379	-1.450935	1.839096
O	3.770439	-1.111345	-2.020539
H	1.510931	0.001156	-1.506896
H	-0.818423	0.004271	-0.588603
H	-2.087653	0.005800	1.960948
H	-1.533294	0.020667	4.383172
H	2.674209	0.014244	3.512862
H	3.998929	0.768919	-0.180962
H	4.012243	0.438948	1.535884
H	8.226318	0.838570	-0.416995
H	7.865565	0.694914	1.327689
H	8.504774	-0.709135	0.433130
H	6.315288	-3.690312	-2.856620
H	4.582852	-3.392081	-3.176015
H	5.776658	-2.099952	-3.470370
H	3.823699	-2.155949	2.159784
H	2.755842	-2.518278	0.814331
H	5.492324	-3.887645	1.212366
H	4.238573	-5.891732	0.669285
H	2.691021	-4.897641	0.474679
H	1.961942	0.036820	7.051733
H	2.615490	-0.873106	5.668604
H	2.628528	0.913499	5.653535

3i (T)
Opt @ uB3LYP/6-311+G(2d,p) in Ethyl Acetate (SMD model)
SCF Done: E(UB3LYP) = -1129.66822175 a.u.
Zero-point correction = 0.357262 Hartree/Particle
Sum of electronic and thermal Free Energies = -1129.368405 a.u.

C	0.000000	0.000000	0.000000
C	0.000000	0.000000	1.445712
C	1.248180	0.000000	2.146674
C	2.456988	-0.001245	1.506400
C	2.434895	-0.004828	0.039718
C	1.240606	-0.002846	-0.661930
C	-1.025596	0.009224	2.381343
C	-0.410141	0.015883	3.706513
N	0.940706	0.005622	3.508504
C	-2.490684	0.076112	2.099162
C	-3.353376	-1.200911	2.423576
C	-2.564460	-2.490071	2.061624
C	-3.249599	-3.811031	2.265388

C	-2.712866	-4.825578	2.934994
O	1.356060	-0.004108	-2.030752
C	0.166818	-0.024835	-2.812462
C	-3.768797	-1.213786	3.900696
O	-3.265278	-0.545146	4.771696
C	-4.610398	-1.067799	1.550815
O	-4.831347	-1.704234	0.549246
O	-4.761432	-2.083911	4.114268
C	-5.225680	-2.213132	5.475528
O	-5.406108	-0.090376	2.005108
C	-6.602930	0.174575	1.240225
H	-0.862830	0.019769	4.680175
H	1.628593	0.012950	4.246850
H	3.399969	0.001484	2.036717
H	3.366424	-0.009405	-0.511401
H	-0.932706	-0.000391	-0.543000
H	-2.927073	0.914294	2.646996
H	-2.617699	0.288076	1.036093
H	-7.105955	0.984542	1.762476
H	-6.345502	0.478289	0.225936
H	-7.236723	-0.711186	1.212054
H	-6.018281	-2.956473	5.440860
H	-4.416476	-2.551632	6.121735
H	-5.612935	-1.260016	5.834730
H	-2.296882	-2.393785	1.004626
H	-1.630704	-2.480128	2.626461
H	-4.217812	-3.938792	1.791998
H	-3.222635	-5.779264	3.018990
H	-1.744385	-4.742732	3.419769
H	0.493721	-0.029916	-3.851363
H	-0.426692	-0.923805	-2.619795
H	-0.447866	0.863164	-2.635792

3j

Opt @ uB3LYP/6-311+G(2d,p) in Ethyl Acetate (SMD model)
SCF Done: E(UB3LYP) = -3588.76441932 a.u.
Zero-point correction = 0.320942 Hartree/Particle
Sum of electronic and thermal Free Energies = -3588.499780 a.u.

N	0.000000	0.000000	0.000000
C	0.000000	0.000000	1.372785
C	1.357599	0.000000	1.789607
C	2.173030	-0.001895	0.601994
C	1.297169	0.003703	-0.455480
C	-1.056669	0.004712	2.281625
C	-0.752075	0.013067	3.633833
C	0.588139	0.018384	4.047441
C	1.647365	0.011345	3.163235
Br	0.956137	0.035697	5.944287
C	3.674300	0.052366	0.558915
C	4.452398	-1.284024	0.272854
C	5.887646	-1.034519	0.757855
O	6.520275	-0.163577	-0.041105
C	7.866282	0.198119	0.338075
C	3.811159	-2.467582	1.048852
C	4.431435	-3.826527	0.892533
C	3.747561	-4.918505	0.567101
C	4.474464	-1.578138	-1.232776
O	5.413019	-2.482766	-1.535973

C	5.509668	-2.873025	-2.922257
O	3.735871	-1.083220	-2.050818
O	6.374035	-1.500125	1.759970
H	1.507217	0.002057	-1.511889
H	-0.819622	0.004964	-0.587690
H	-2.088360	0.002930	1.950095
H	-1.547769	0.016545	4.366329
H	2.669706	0.015426	3.517860
H	4.000698	0.784236	-0.179664
H	4.020995	0.417845	1.528935
H	8.203675	0.895743	-0.424528
H	7.869717	0.675792	1.317477
H	8.504876	-0.684595	0.354941
H	6.317673	-3.599411	-2.962596
H	4.576170	-3.325680	-3.255248
H	5.744056	-2.008810	-3.543363
H	3.846700	-2.189095	2.106580
H	2.758717	-2.518551	0.767613
H	5.492474	-3.907685	1.106125
H	4.227277	-5.889624	0.509536
H	2.683710	-4.883431	0.350390

3j (T)
Opt @ uB3LYP/6-311+G(2d,p) in Ethyl Acetate (SMD model)
SCF Done: E(UB3LYP) = -3588.65392440 a.u.
Zero-point correction = 0.315241 Hartree/Particle
Sum of electronic and thermal Free Energies = -3588.396859 a.u.

C	0.000000	0.000000	0.000000
C	0.000000	0.000000	1.443336
C	1.244675	0.000000	2.138762
C	2.467525	-0.000241	1.504354
C	2.449661	-0.002862	0.037975
C	1.266067	-0.001414	-0.636949
C	-1.027123	0.007210	2.386195
C	-0.403590	0.012259	3.699679
N	0.940460	0.005159	3.492712
C	-2.492747	0.075194	2.105834
C	-3.350641	-1.212444	2.395205
C	-3.753929	-1.274731	3.874319
O	-3.205091	-0.677658	4.769735
Br	1.282227	-0.003927	-2.569528
C	-4.617387	-1.061593	1.537857
O	-4.875563	-1.714657	0.556657
C	-2.561689	-2.490574	1.994154
C	-3.232291	-3.817818	2.205398
C	-2.678956	-4.825153	2.872395
O	-5.377152	-0.053327	1.985766
C	-6.582597	0.225599	1.239315
O	-4.787944	-2.101751	4.057864
C	-5.239430	-2.281049	5.418215
H	-0.847062	0.013401	4.677622
H	1.631264	0.011569	4.229986
H	3.407795	0.001887	2.038022
H	3.390781	-0.005656	-0.494546
H	-0.917906	0.000993	-0.568306
H	-2.930654	0.897921	2.674910
H	-2.619181	0.316613	1.048868
H	-7.049091	1.065565	1.747910

H	-6.338880	0.491237	0.211126
H	-7.243058	-0.640957	1.251523
H	-6.069718	-2.980091	5.356846
H	-4.439519	-2.694843	6.031239
H	-5.572274	-1.330411	5.834018
H	-2.324528	-2.380676	0.931219
H	-1.613404	-2.480948	2.533969
H	-4.204195	-3.954367	1.742607
H	-3.178661	-5.783327	2.964886
H	-1.705501	-4.731695	3.345210

X-Ray crystal data

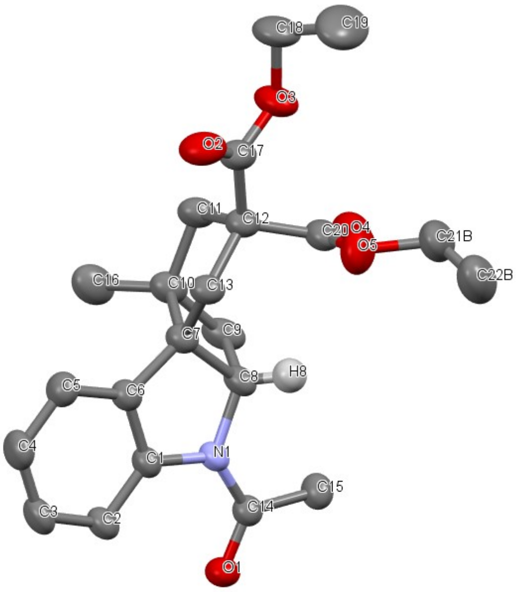

Figure S16. X-Ray structure of **5g**

Crystals of **5g** suitable for XRD analysis were obtained by slow evaporation of $CHCl_3$/hexane solution.

Table S2. Crystal data and structure refinement for **5g**

Empirical formula	$C_{22}H_{27}NO_5$
Formula weight	385.45
Temperature/K	293(2)
Crystal system	triclinic
Space group	P-1
a/Å	7.4056(2)
b/Å	9.4530(3)
c/Å	15.2930(5)
α/°	84.544(2)
β/°	81.654(3)
γ/°	83.321(3)
Volume/Å^3	1048.73(6)
Z	2
ρ_{calc}g/cm^3	1.221
μ/mm^{-1}	0.703
F(000)	412.0
Crystal size/mm^3	0.356 × 0.281 × 0.207
Radiation	CuK$_\alpha$ (λ = 1.54184)
2Θ range for data collection/°	5.86 to 147.852
Index ranges	-9 ≤ h ≤ 9, -11 ≤ k ≤ 11, -18 ≤ l ≤ 18
Reflections collected	19741
Independent reflections	4169 [R_{int} = 0.0199, R_{sigma} = 0.0126]
Data/restraints/parameters	4169/9/265
Goodness-of-fit on F^2	1.062
Final R indexes [I>=2σ (I)]	R_1 = 0.0596, wR_2 =0.1717
Final R indexes [all data]	R_1 = 0.0678, wR_2 = 0.1810
Largest diff. peak/hole / e Å^{-3}	0.30/-0.29

References

1 N. V. Tzouras, E. A. Martynova, X. Ma, T. Scattolin, B. Hupp, H. Busen, M. Saab, Z. Zhang, L. Falivene, G. Pisanò, K. Van Hecke, L. Cavallo, C. S. J. Cazin, A. Steffen and S. P. Nolan, *Chem. – Eur. J.*, 2021, **27**, 11904–11911.
2 P. Trillo, A. Baeza and C. Nájera, *Eur. J. Org. Chem.*, 2012, **2012**, 2929–2934.
3 H. Oh, B. Ryou, J. Park, M. Kim, J.-H. Choi and C.-M. Park, *ACS Catal.*, 2021, **11**, 13670–13679.
4 B. Papa Spadafora, F. W. Moreira Ribeiro, J. E. Matsushima, E. M. Ariga, I. Omari, P. M. A. Soares, D. de Oliveira-Silva, E. Vinhato, J. S. McIndoe, T. Carita Correra and A. Rodrigues, *Org. Biomol. Chem.*, 2021, **19**, 5595–5606.
5 Z. Lu and T. P. Yoon, *Angew. Chem. Int. Ed.*, 2012, **51**, 10329–10332.
6 C. Malik, M. Vaultier and S. Ghosh, *Synthesis*, 2007, **2007**, 1247–1250.
7 K. Li, M.-L. Li, Q. Zhang, S.-F. Zhu and Q.-L. Zhou, *J. Am. Chem. Soc.*, 2018, **140**, 7458–7461.
8 Y. He, Y. Cai and S. Zhu, *J. Am. Chem. Soc.*, 2017, **139**, 1061–1064.
9 M. Zhu, K. Zhou, X. Zhang and S.-L. You, *Org. Lett.*, 2018, **20**, 4379–4383.
10 A. B. Rolka and B. Koenig, *Org. Lett.*, 2020, **22**, 5035–5040.
11 M. Zhu, C. Zheng, X. Zhang and S.-L. You, *J. Am. Chem. Soc.*, 2019, **141**, 2636–2644.
12 Q.-F. Wu, C. Zheng and S.-L. You, *Angew. Chem. Int. Ed.*, 2012, **51**, 1680–1683.
13 C. P. Grugel and B. Breit, *Org. Lett.*, 2019, **21**, 5798–5802.
14 X. Li, C. Golz and M. Alcarazo, *Angew. Chem. Int. Ed.*, 2019, **58**, 9496–9500.
15 Z. Yin, Y. Yu, H. Mei and J. Han, *Green Chem.*, 2021, **23**, 3256–3260.
16 F. W. Goetzke, A. M. L. Hell, L. van Dijk and S. P. Fletcher, *Nat. Chem.*, 2021, **13**, 880–886.
17 J. Rabani, H. Mamane, D. Pousty and J. R. Bolton, *Photochem. Photobiol.*, 2021, **97**, 873–902.

NMR spectra

^{1}H NMR (300 MHz, $CDCl_3$) of **1a**

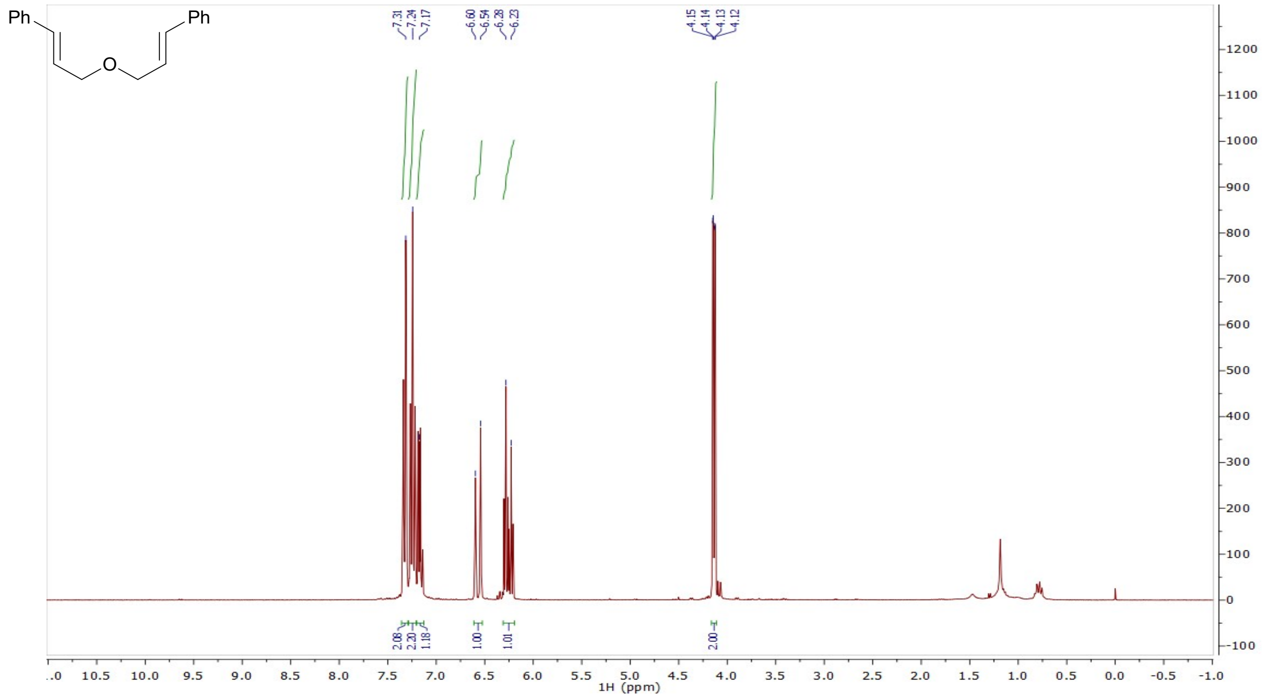

^{1}H NMR (300 MHz, $CDCl_3$) of **1b**

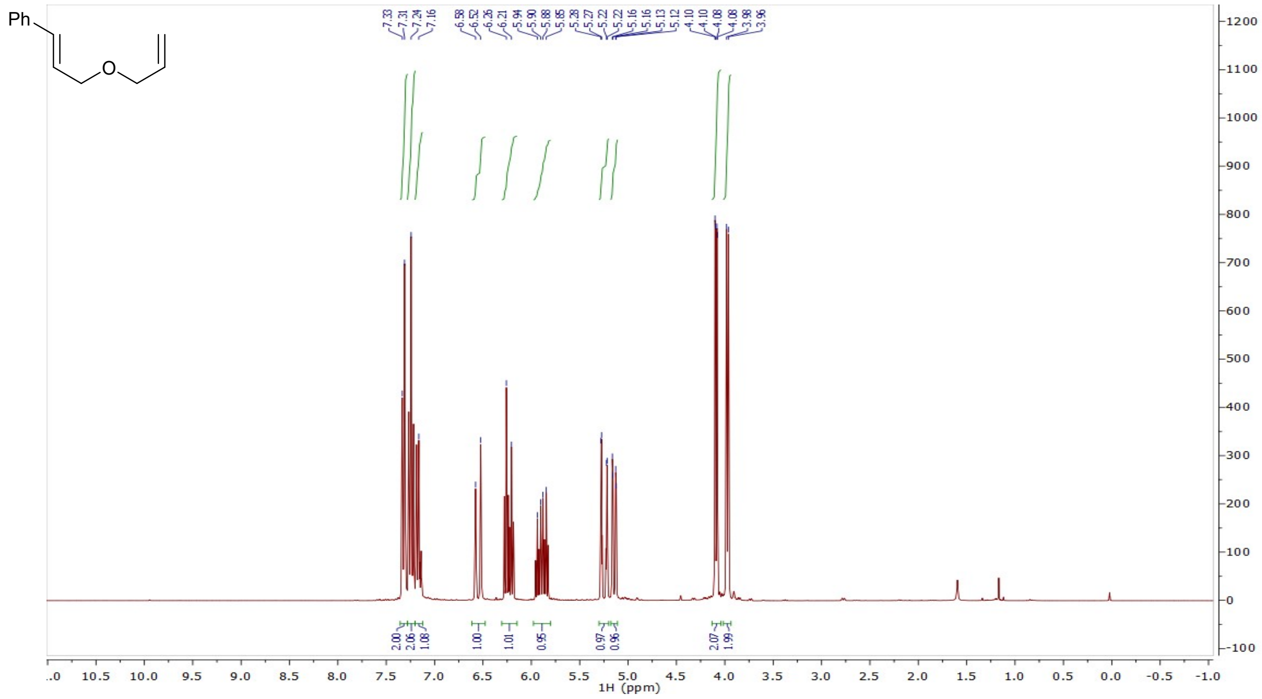

^1H NMR (300 MHz, $CDCl_3$) of **1c**

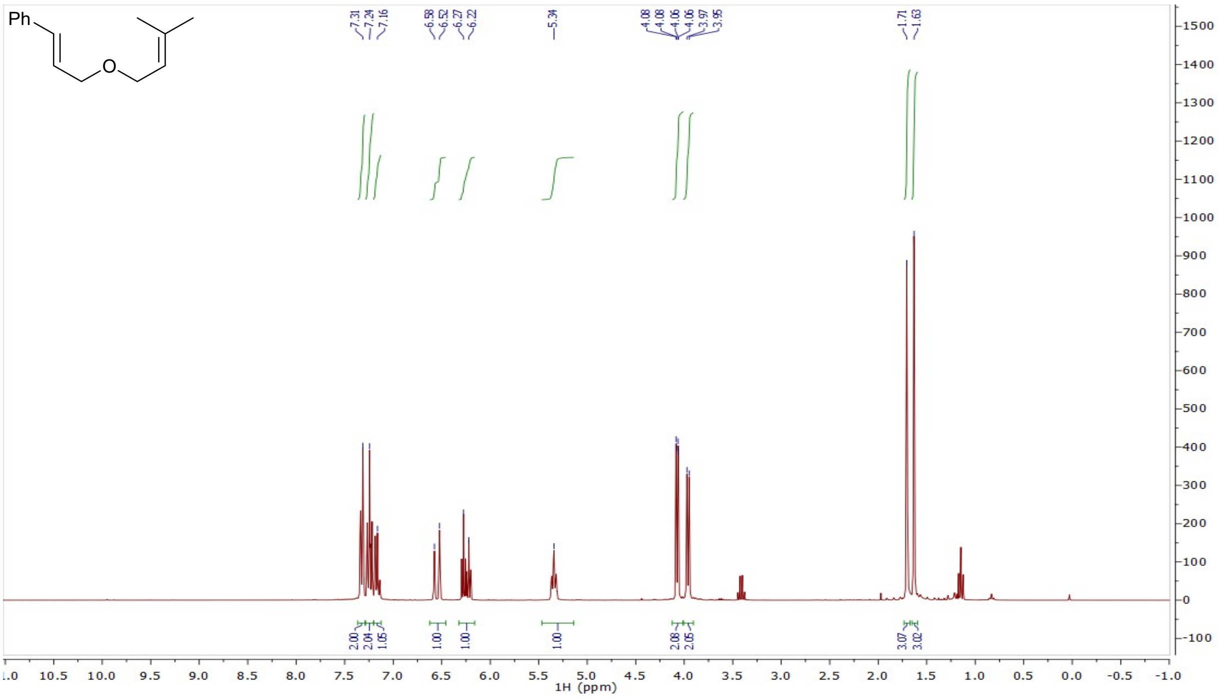

^1H NMR (300 MHz, $CDCl_3$) of **1d**

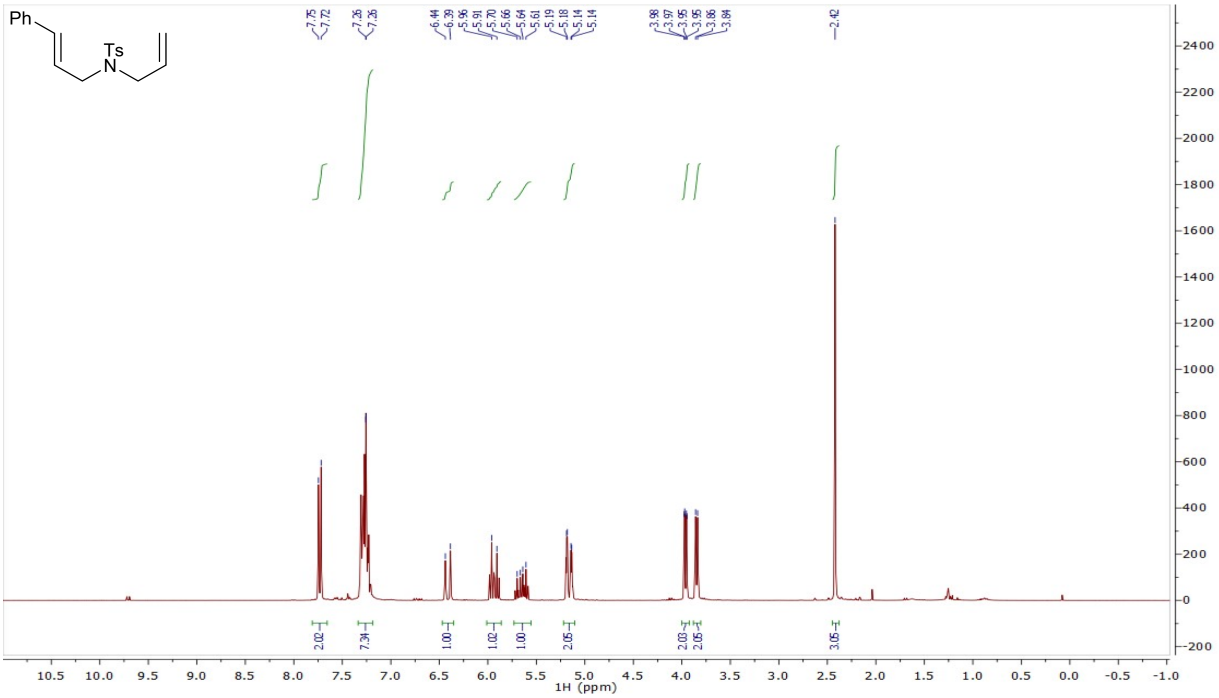

^{1}H NMR (300 MHz, $CDCl_3$) of **1e**

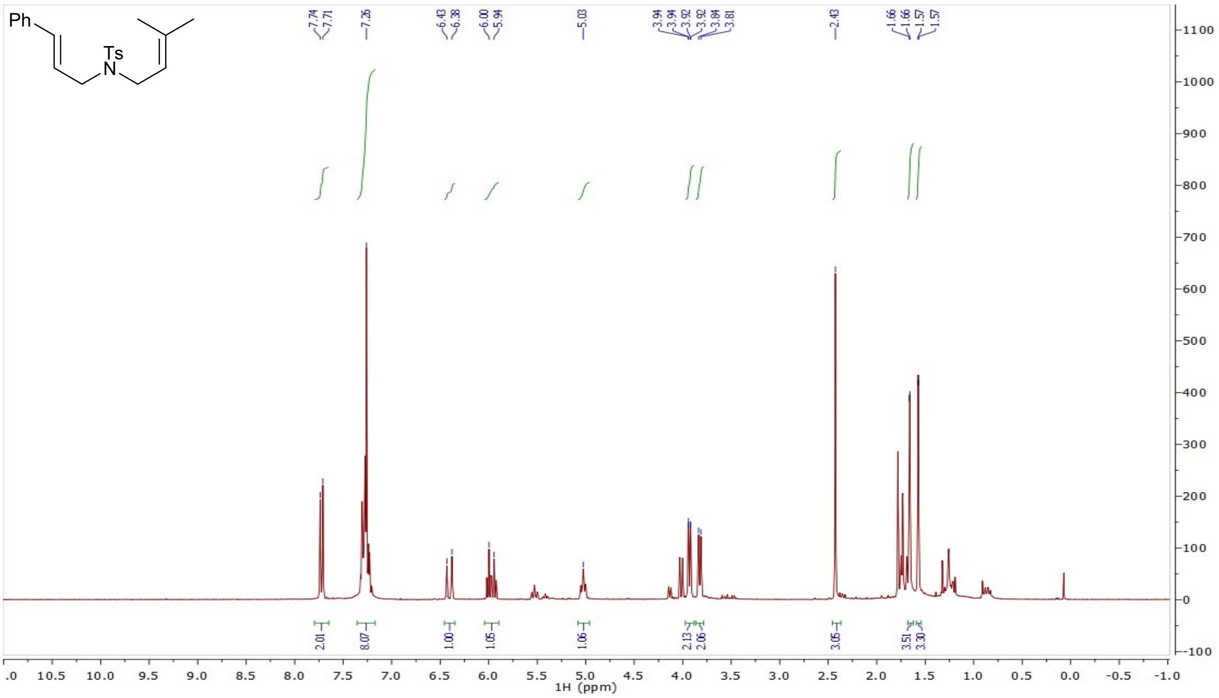

^{1}H NMR (300 MHz, $CDCl_3$) of **1f**

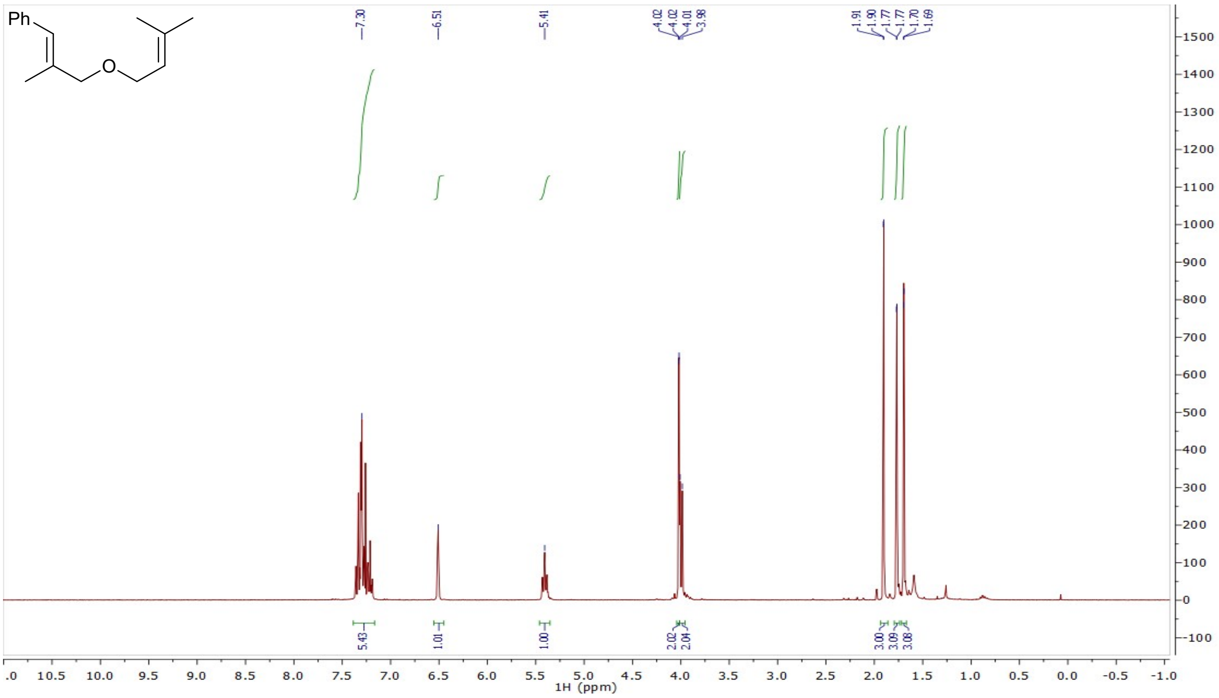

^{1}H NMR (300 MHz, $CDCl_3$) of **1g**

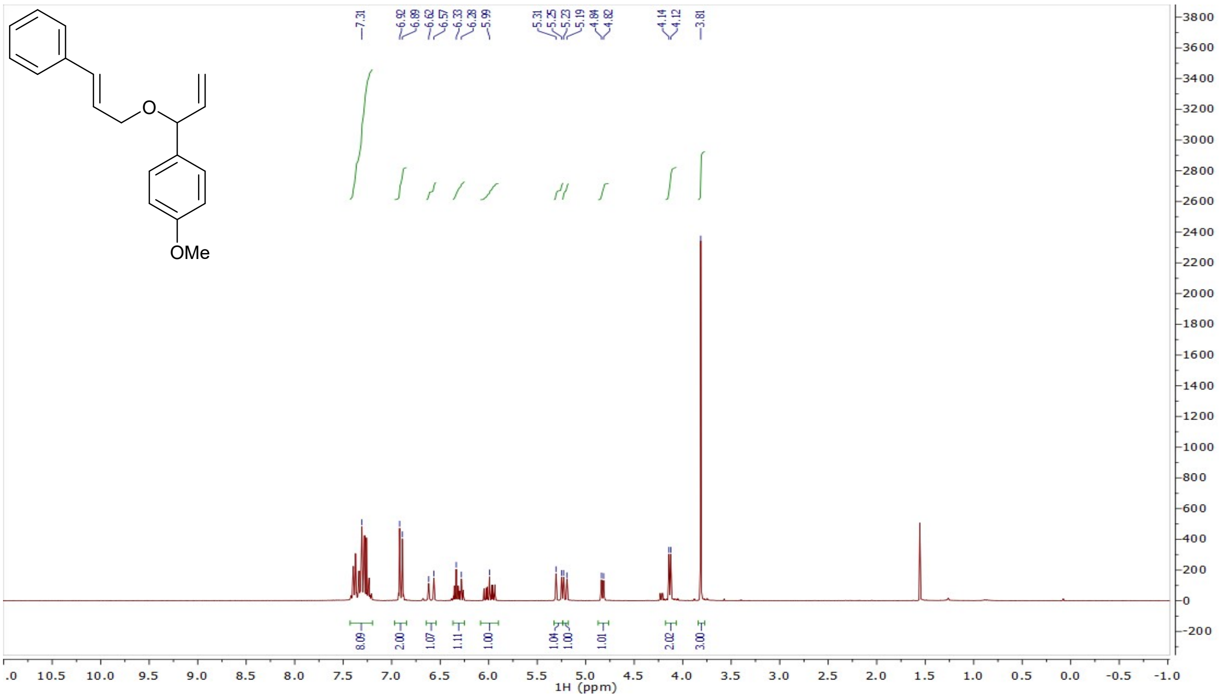

^{1}H NMR (300 MHz, $CDCl_3$) of **1h**

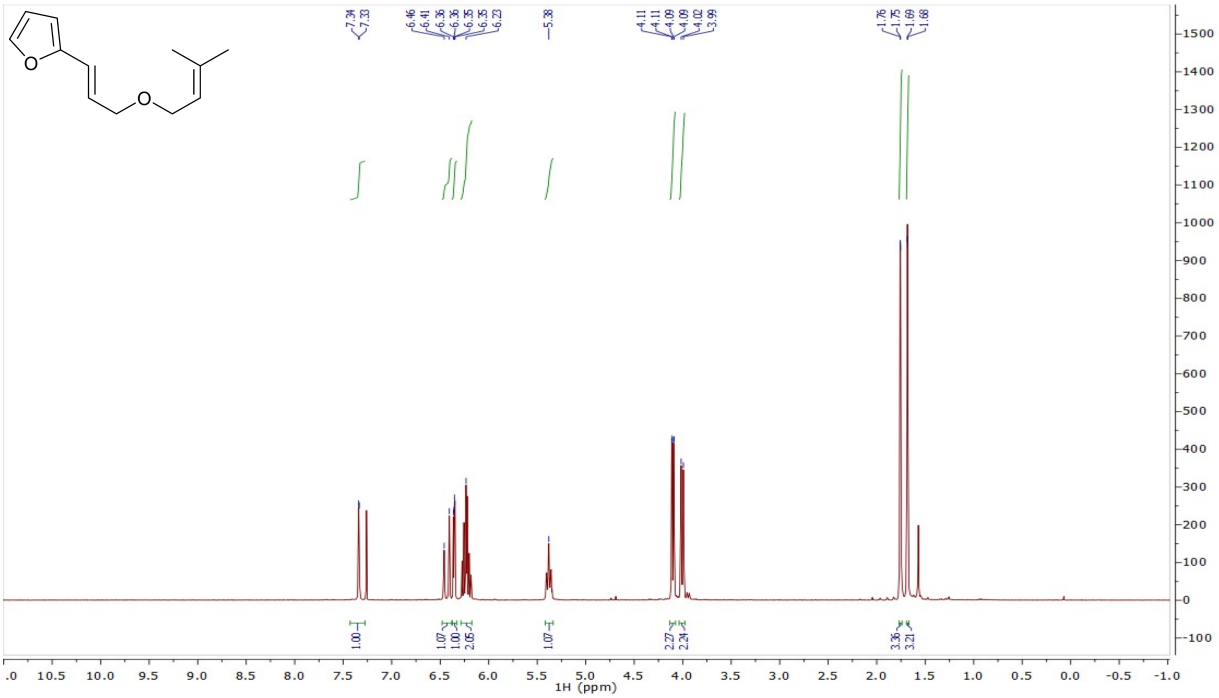

^{1}H NMR (300 MHz, $CDCl_3$) of **1i**

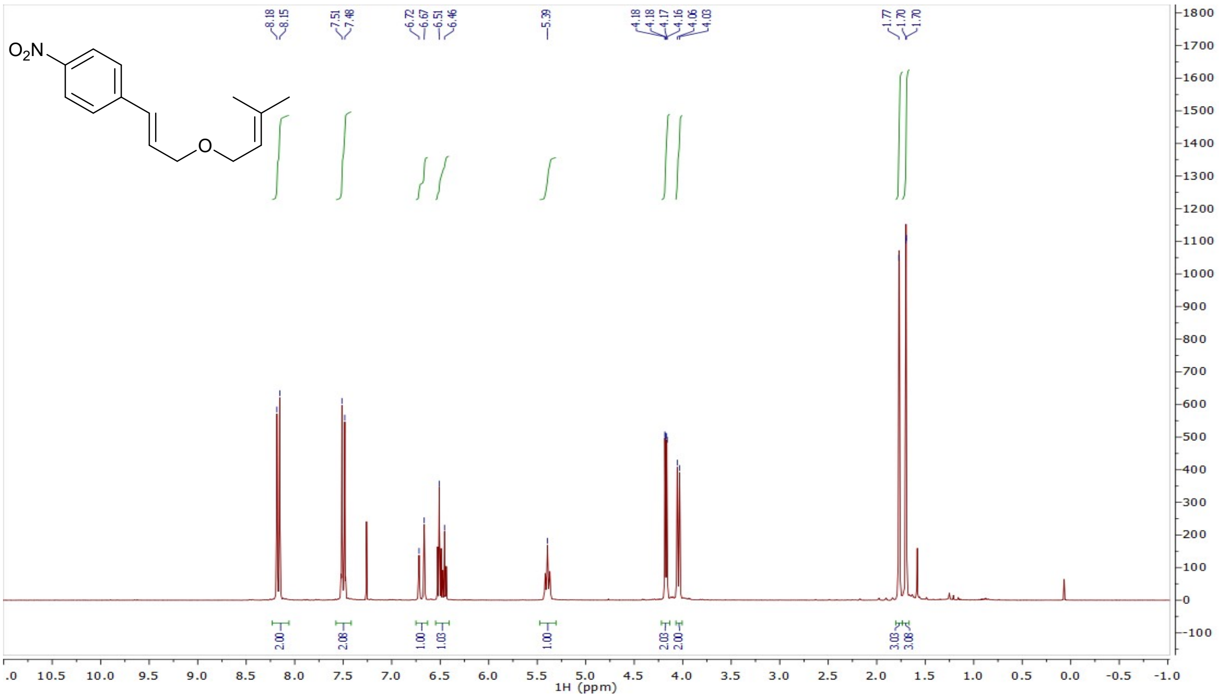

^{1}H NMR (300 MHz, $CDCl_3$) of **1j**

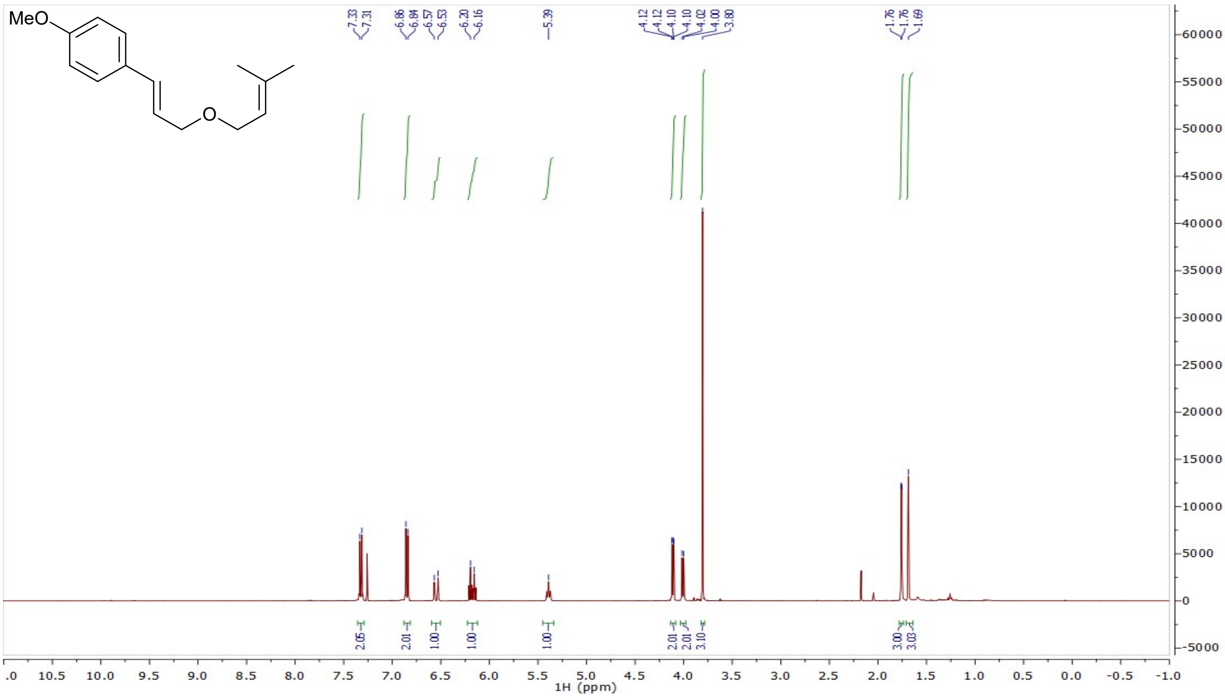

^{1}H NMR (300 MHz, $CDCl_3$) of **S1a**

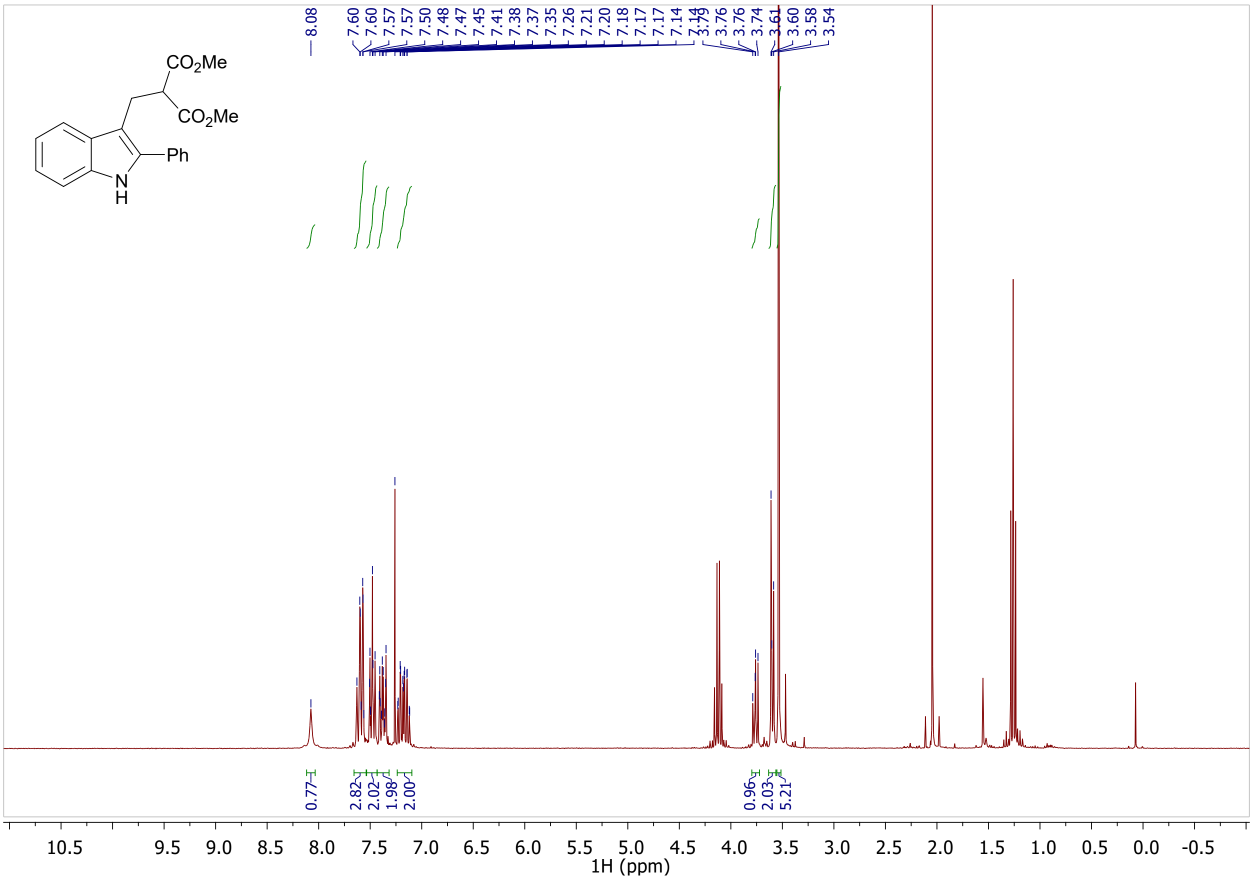

^{1}H NMR (400 MHz, $CDCl_3$) of **S1b**

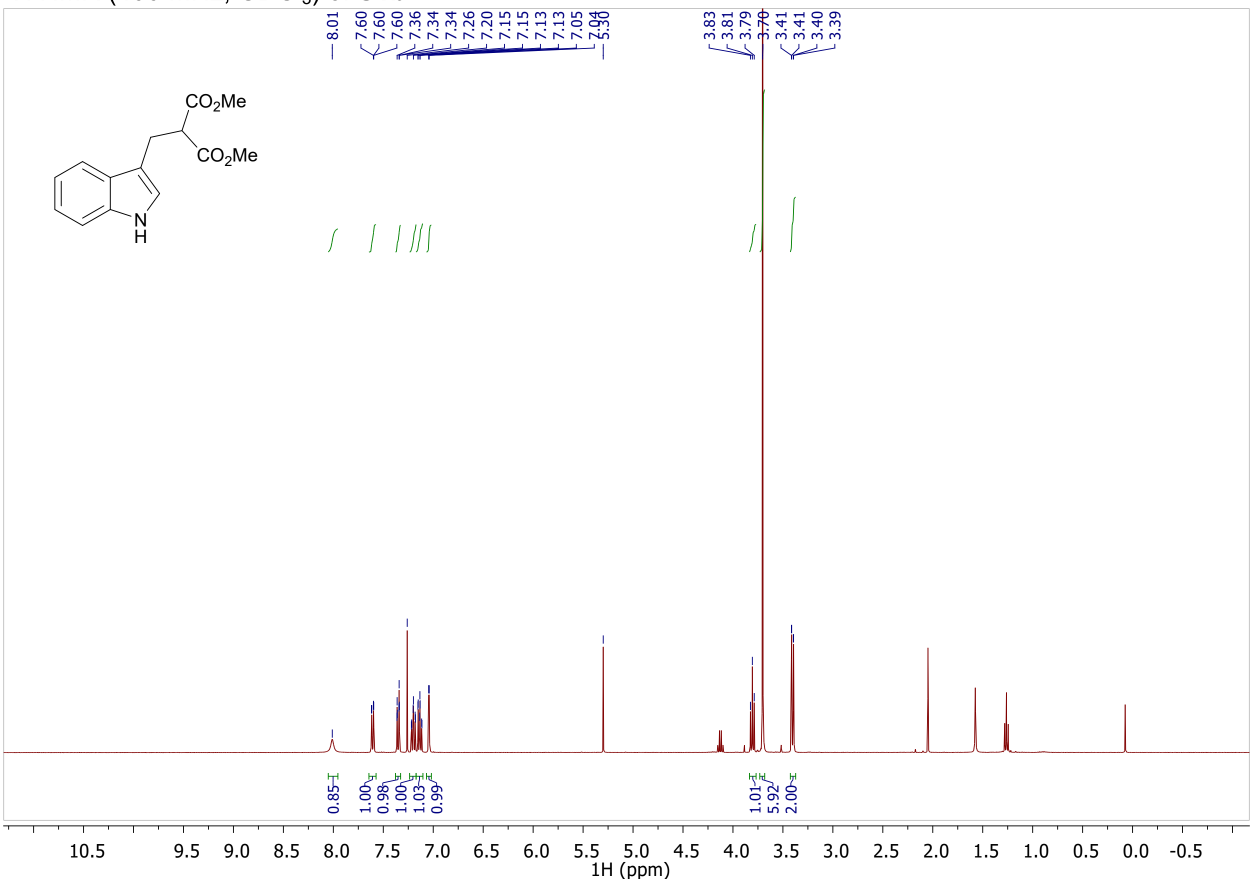

^1H NMR (400 MHz, $CDCl_3$) of **S1c**

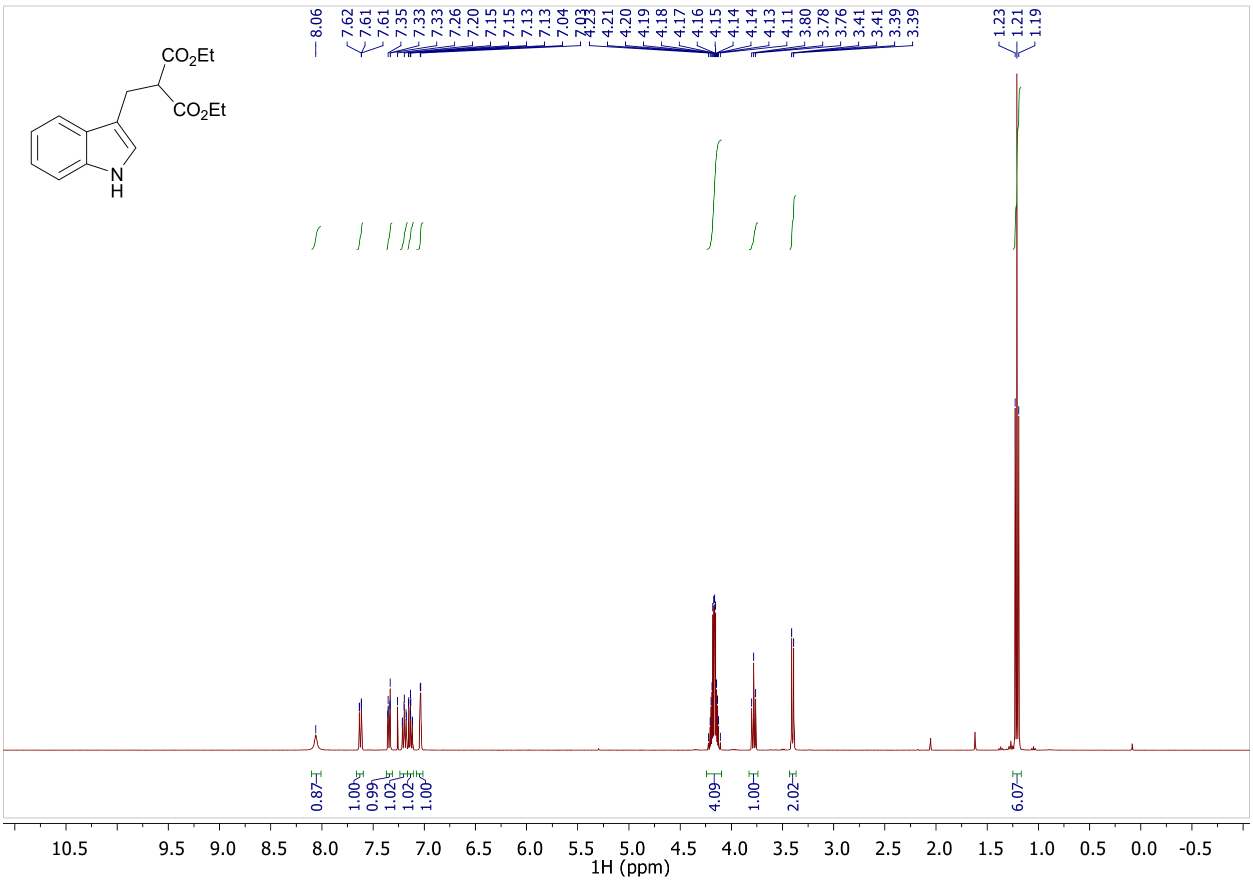

^1H NMR (400 MHz, $CDCl_3$) of **S1d**

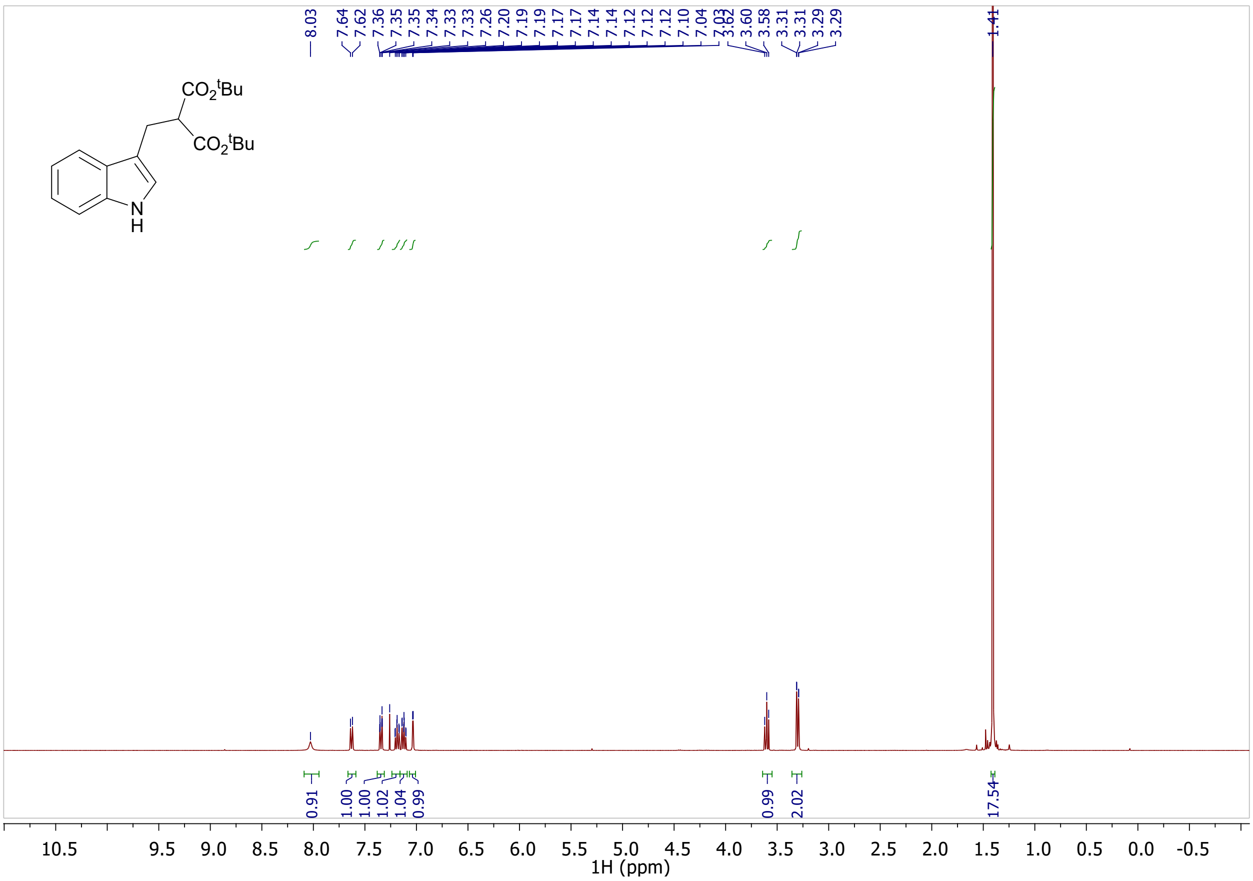

^{1}H NMR (300 MHz, $CDCl_3$) of **S1f**

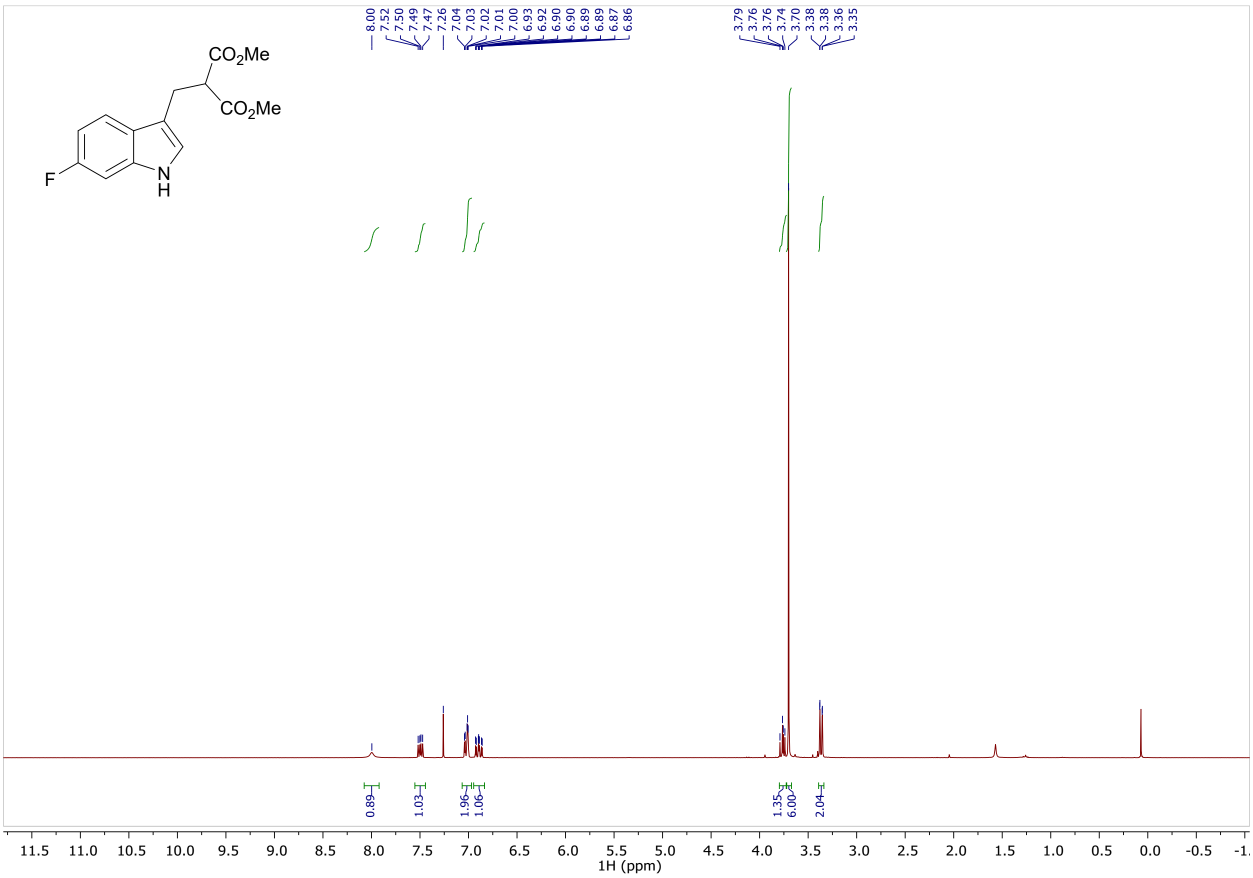

^{13}C NMR (75 MHz, $CDCl_3$) of **S1f**

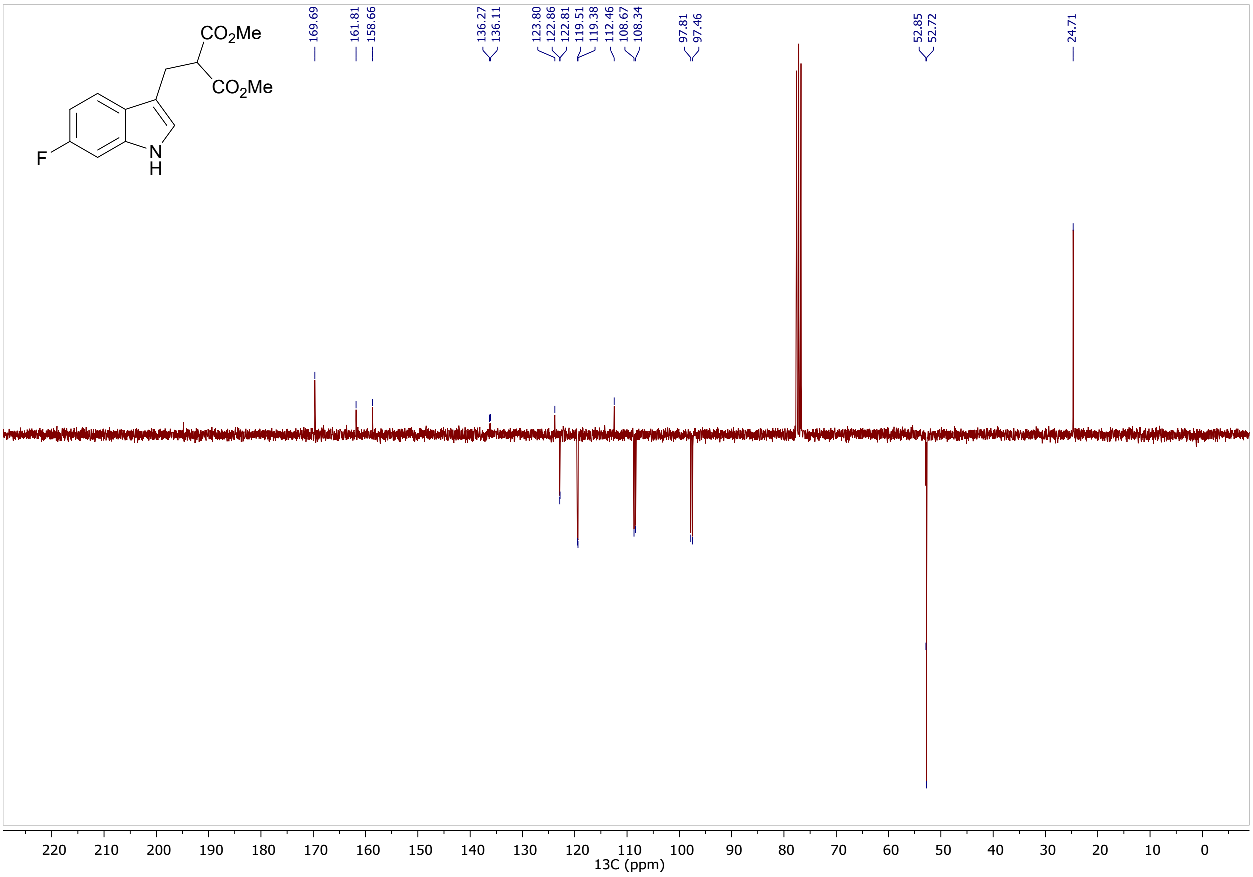

^{19}F NMR (377 MHz, $CDCl_3$) of **S1f**

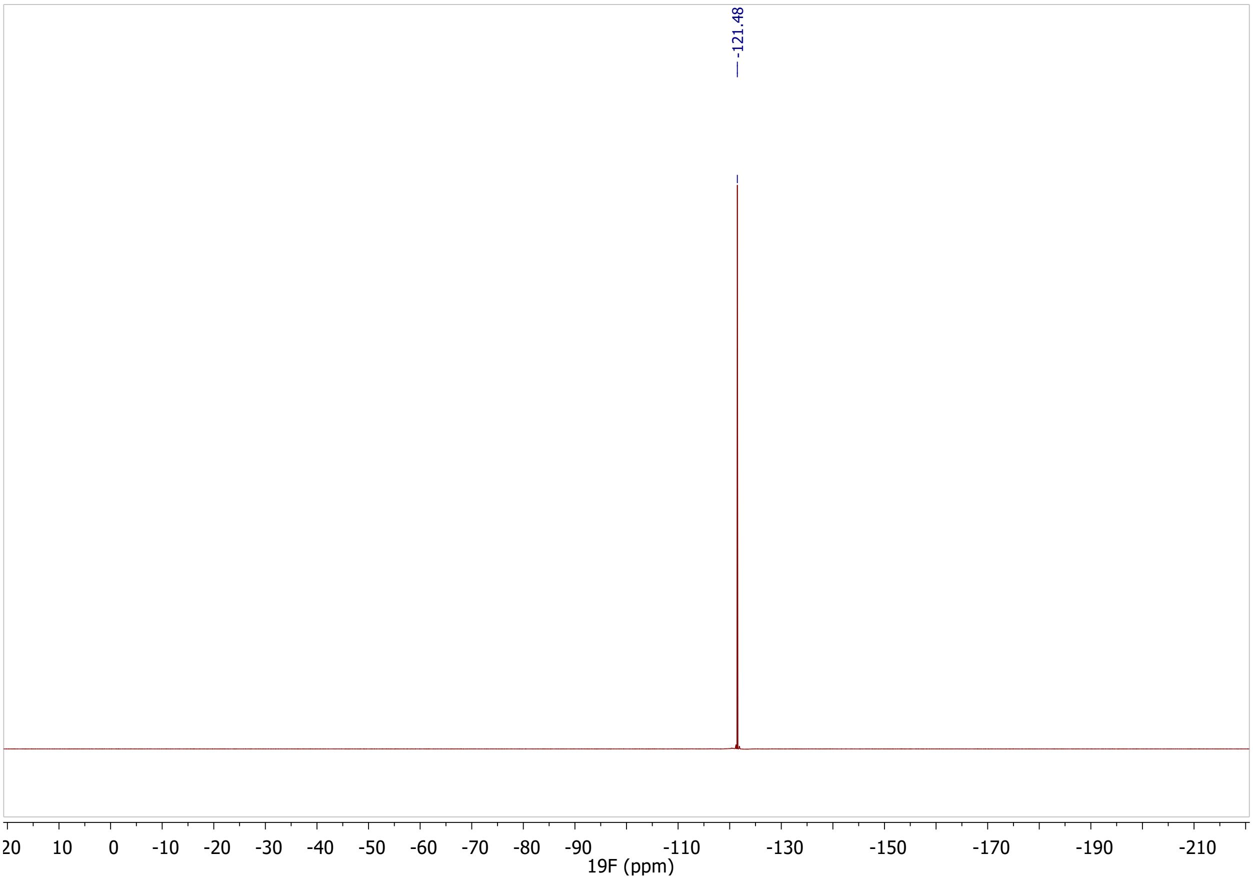

^{1}H NMR (400 MHz, $CDCl_3$) of **S1g**

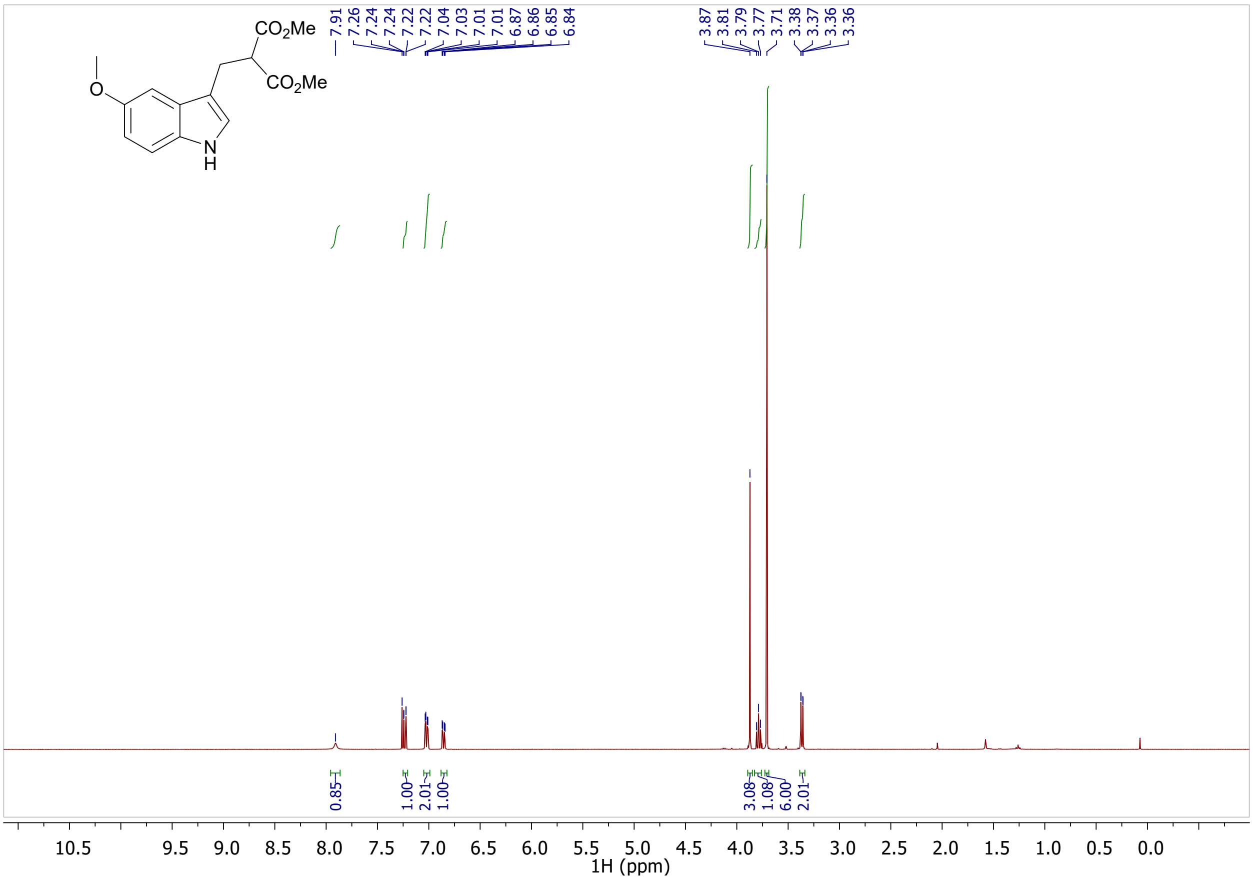

^{1}H NMR (300 MHz, $CDCl_3$) of **S1h**

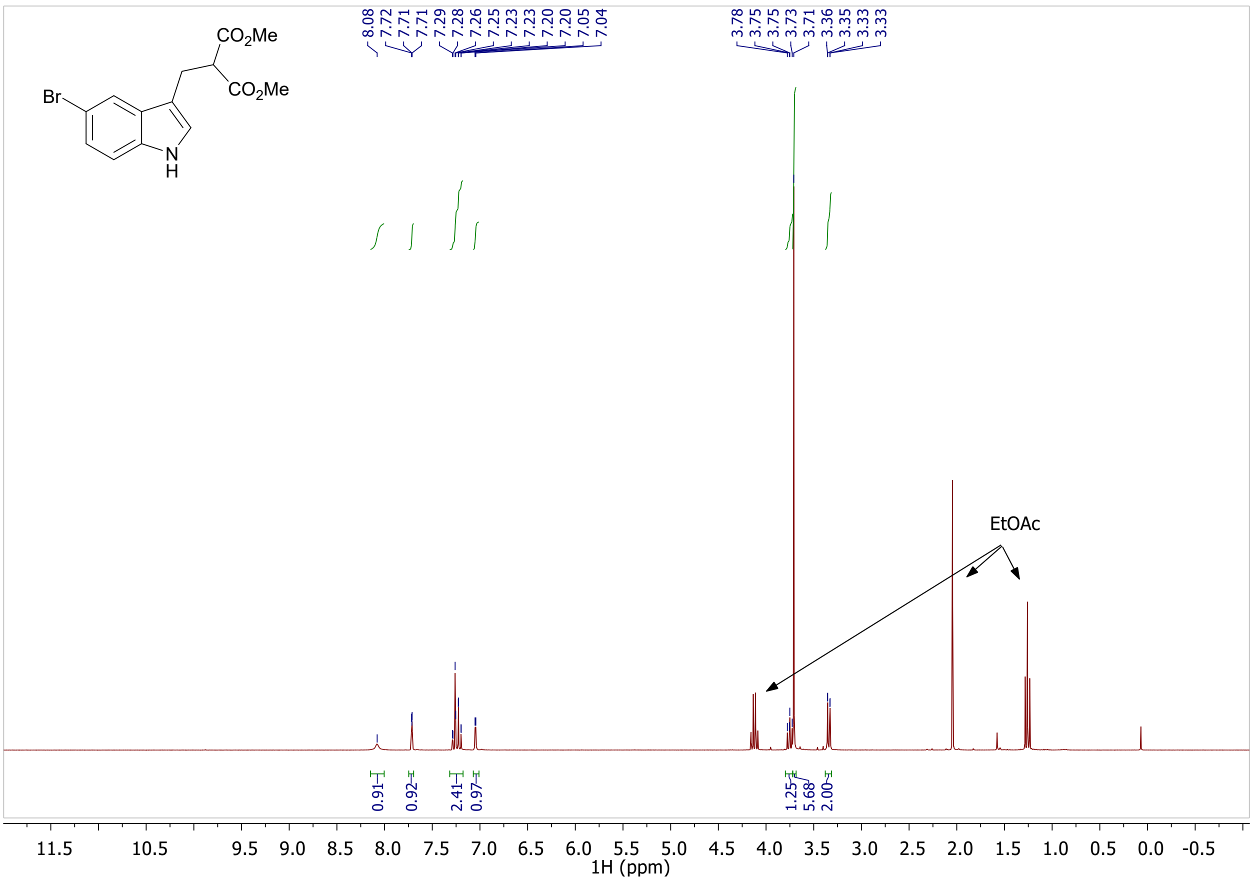

^{1}H NMR (300 MHz, $CDCl_3$) of **S1k**

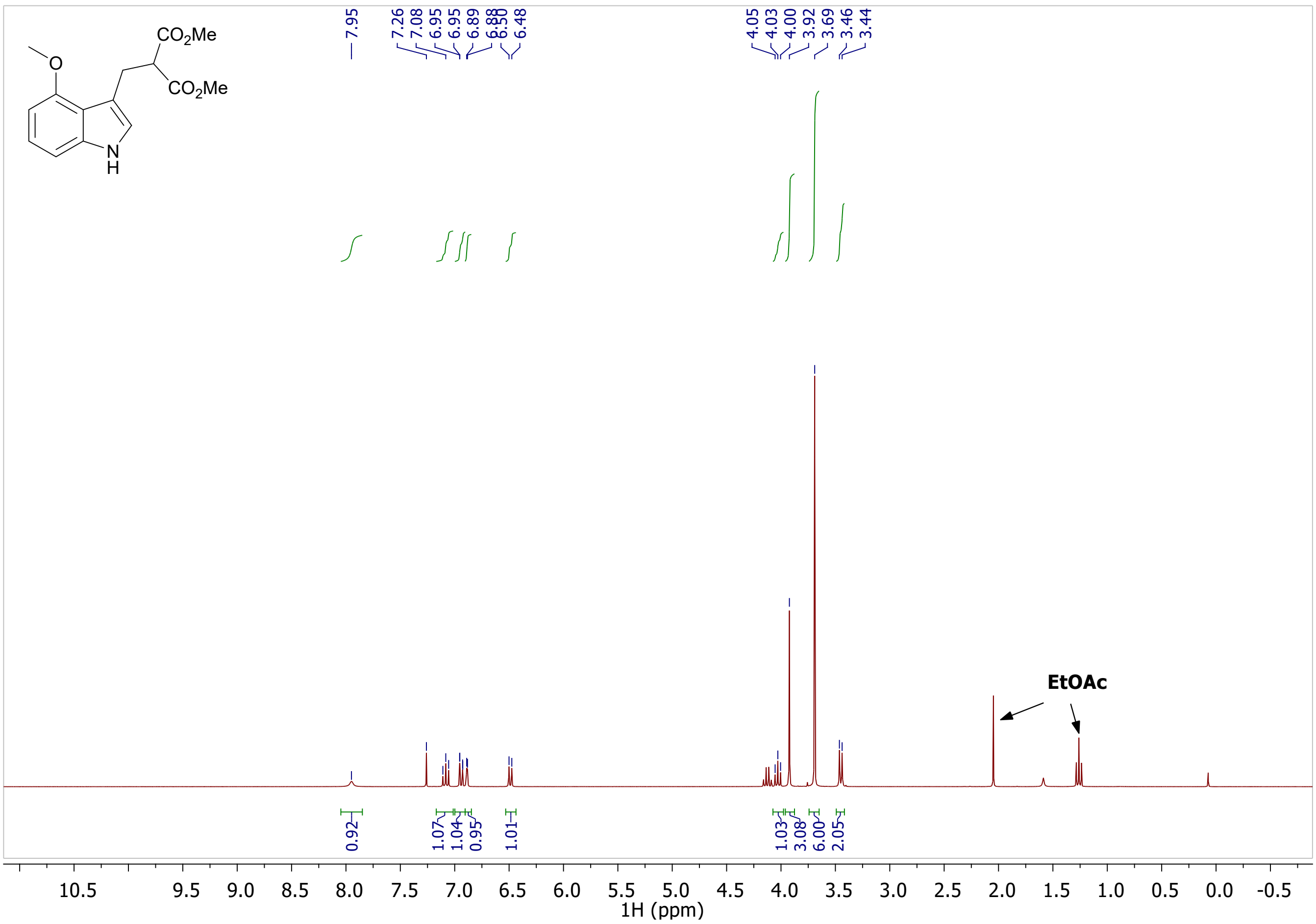

^1H NMR (300 MHz, $CDCl_3$) of **3a**

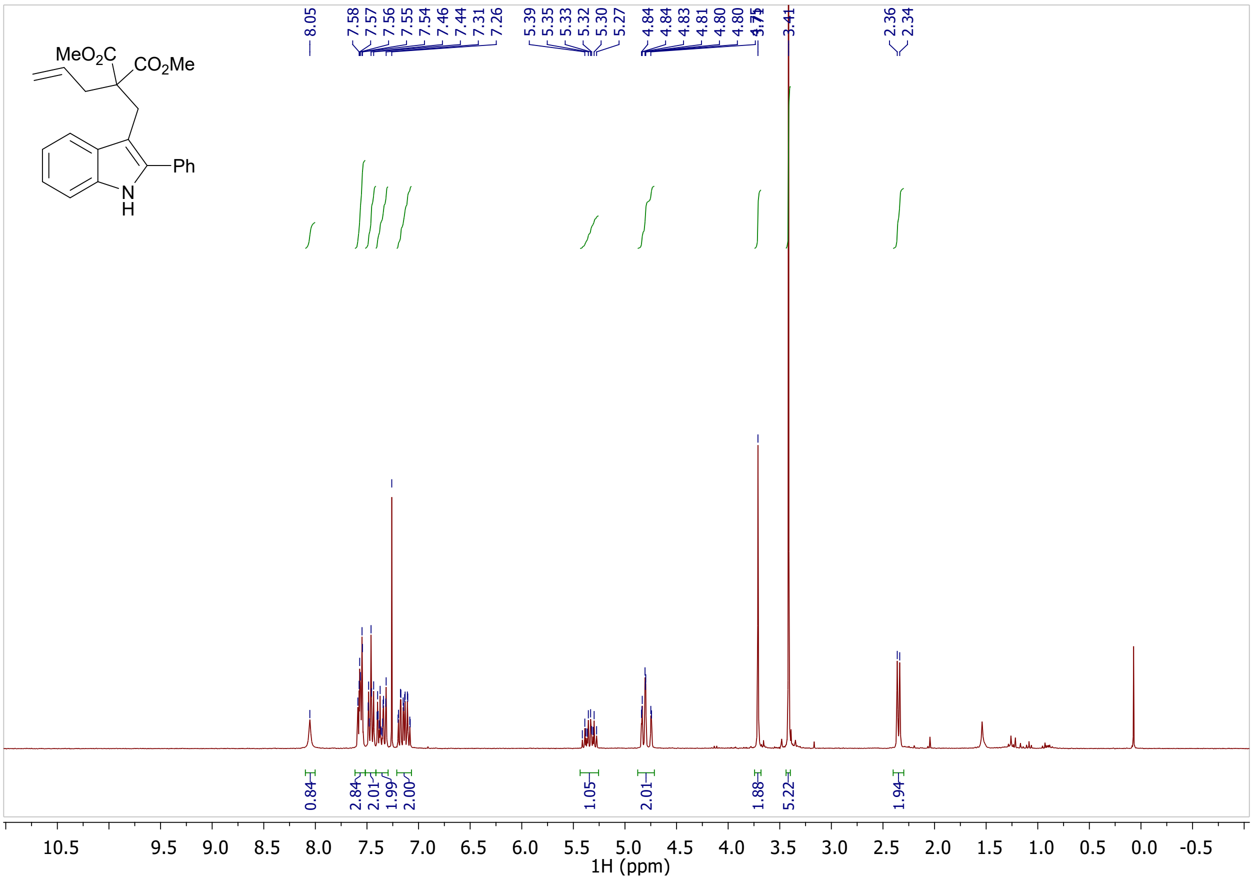

^1H NMR (400 MHz, $CDCl_3$) of **3b**

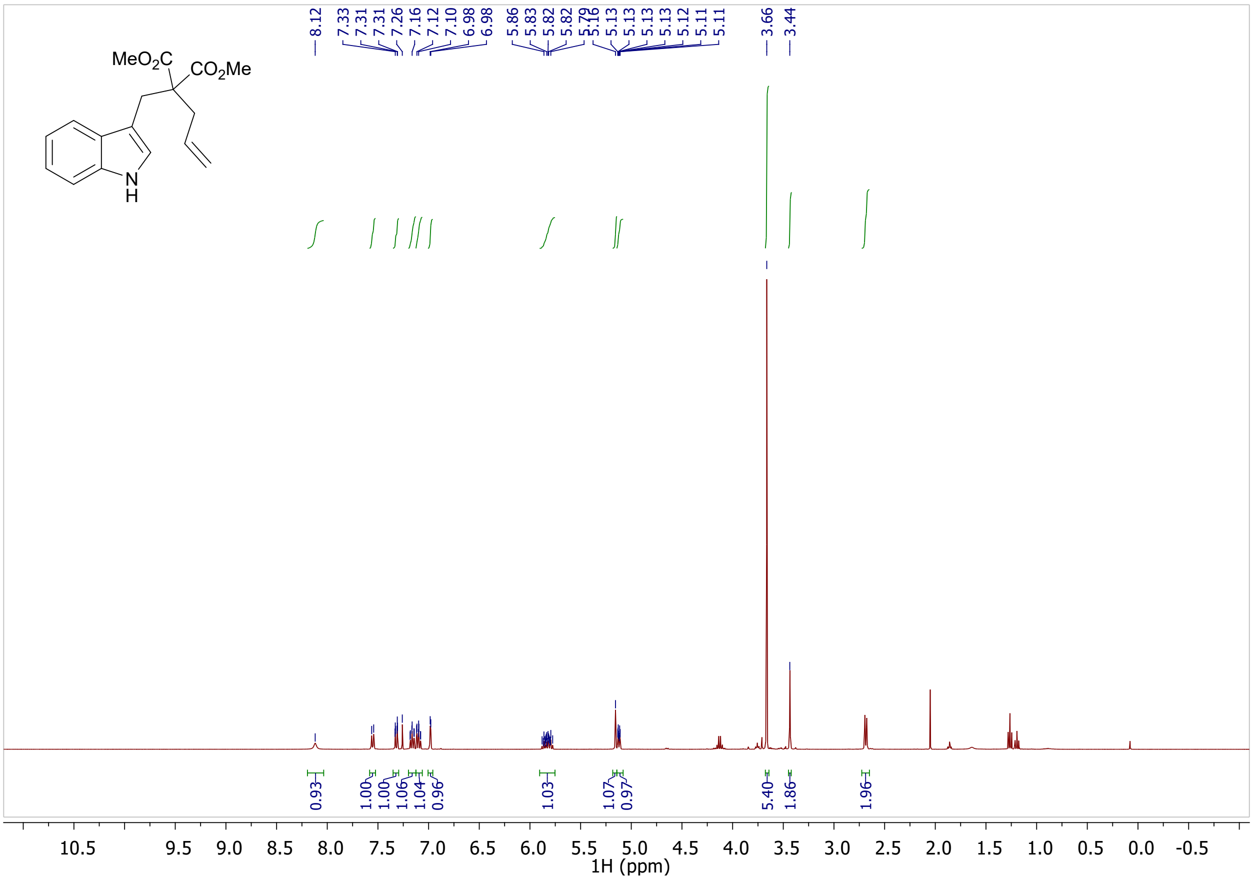

^{1}H NMR (300 MHz, $CDCl_3$) of **3c**

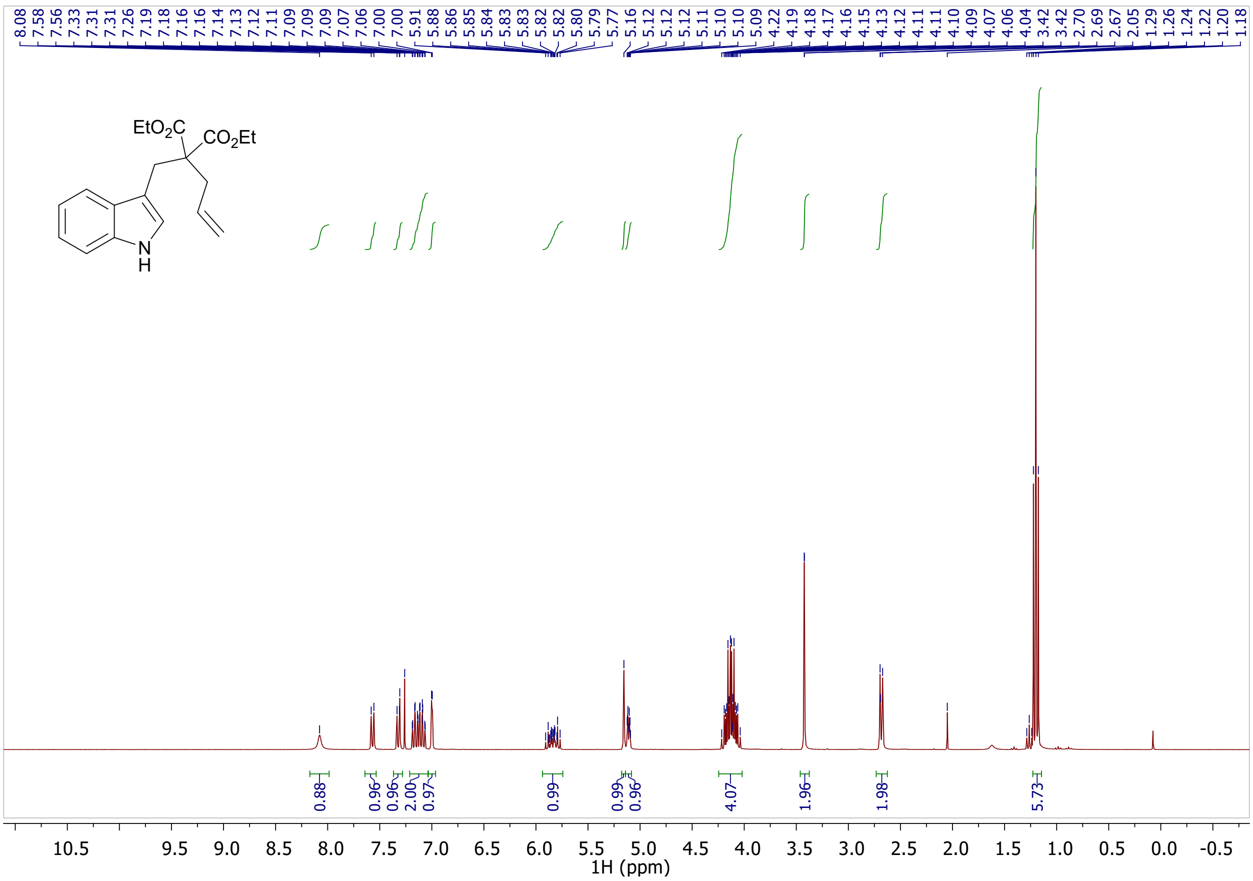

^{1}H NMR (300 MHz, $CDCl_3$) of **3d**

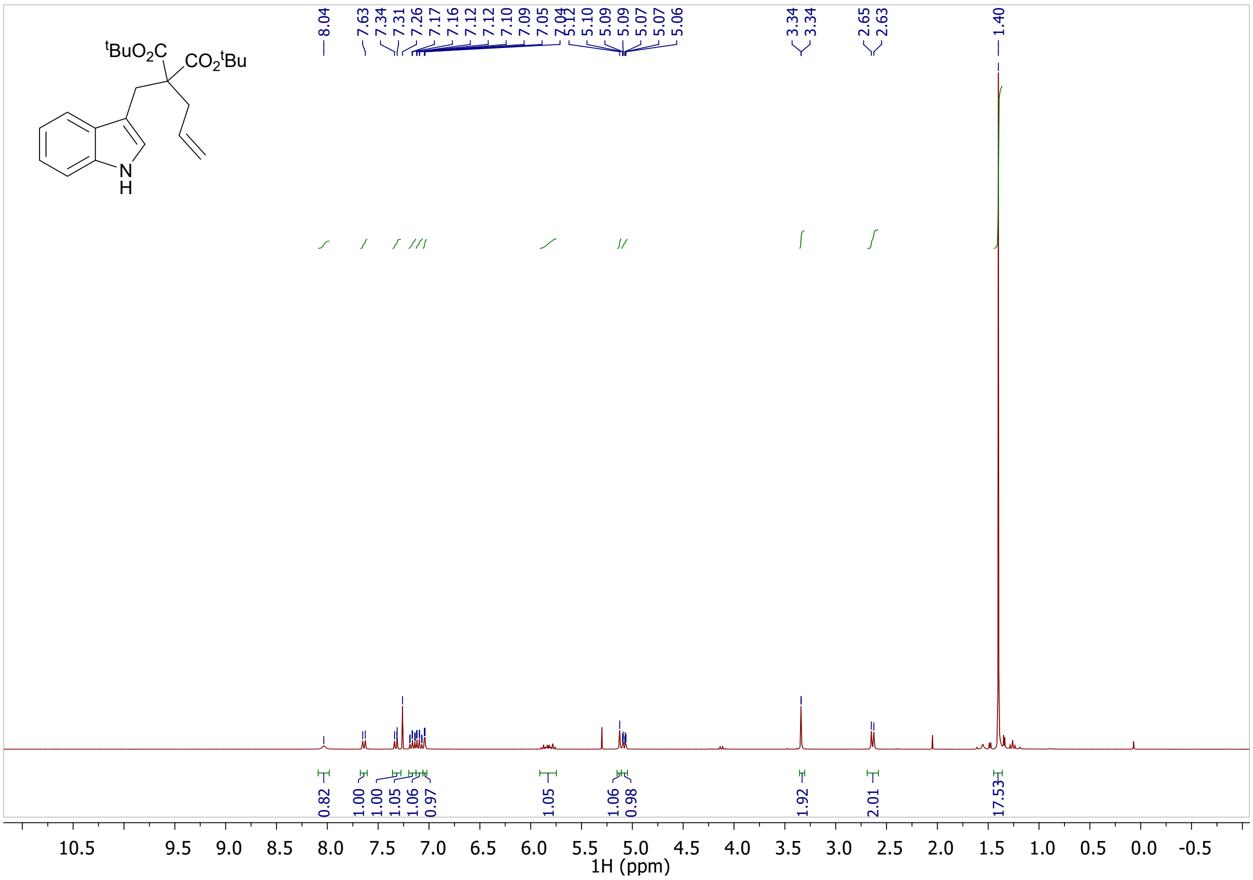

^{13}C NMR (75 MHz, $CDCl_3$) of **3d**

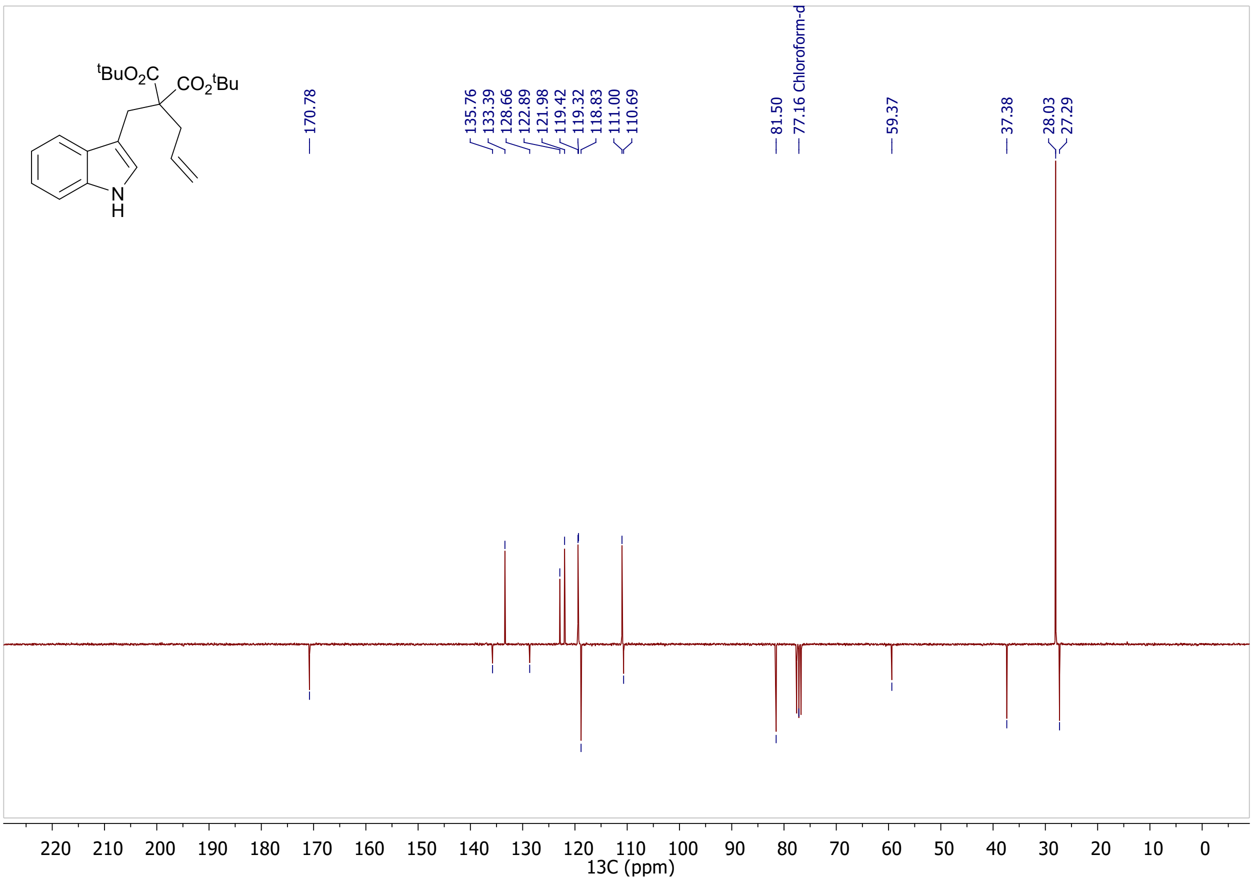

^{1}H NMR (300 MHz, $CDCl_3$) of **3e**

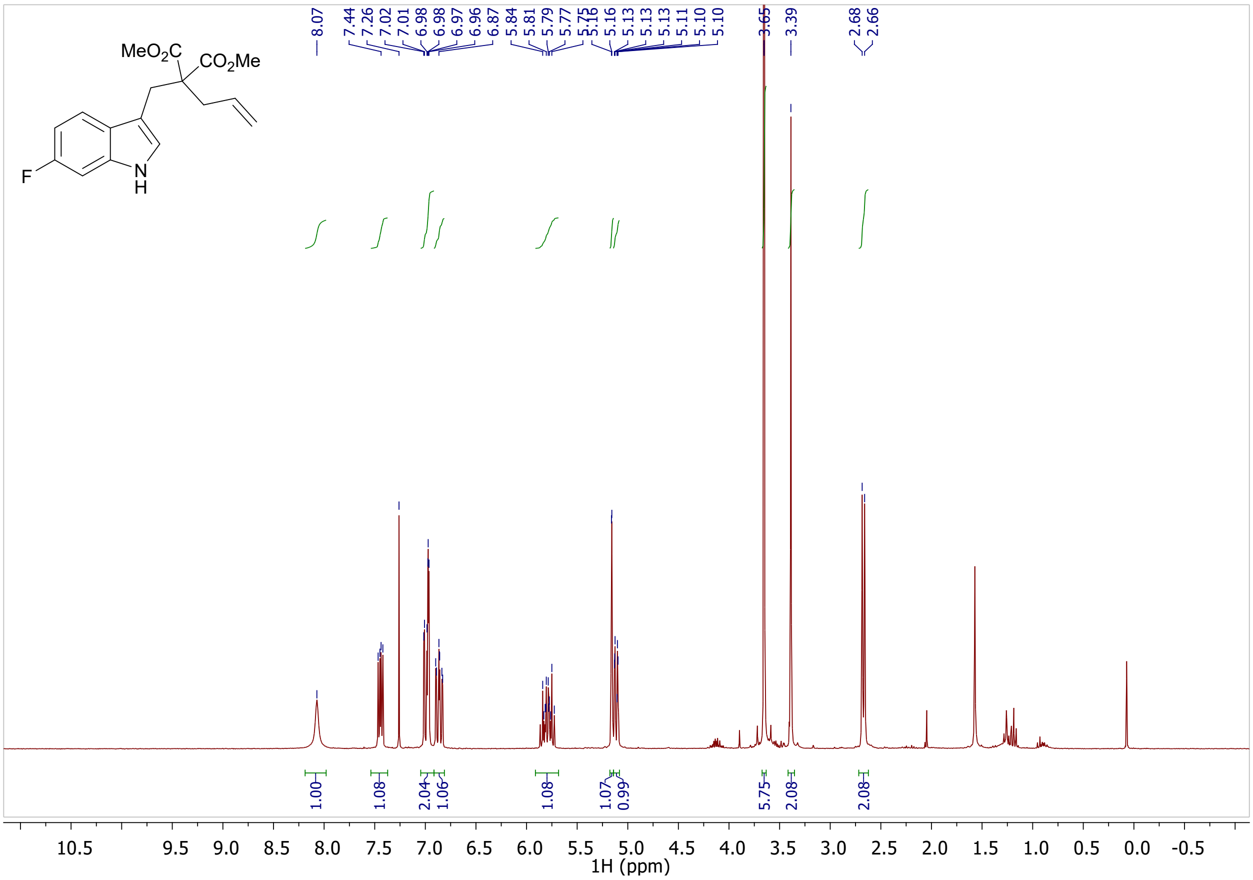

^{13}C NMR (75 MHz, $CDCl_3$) of **3e**

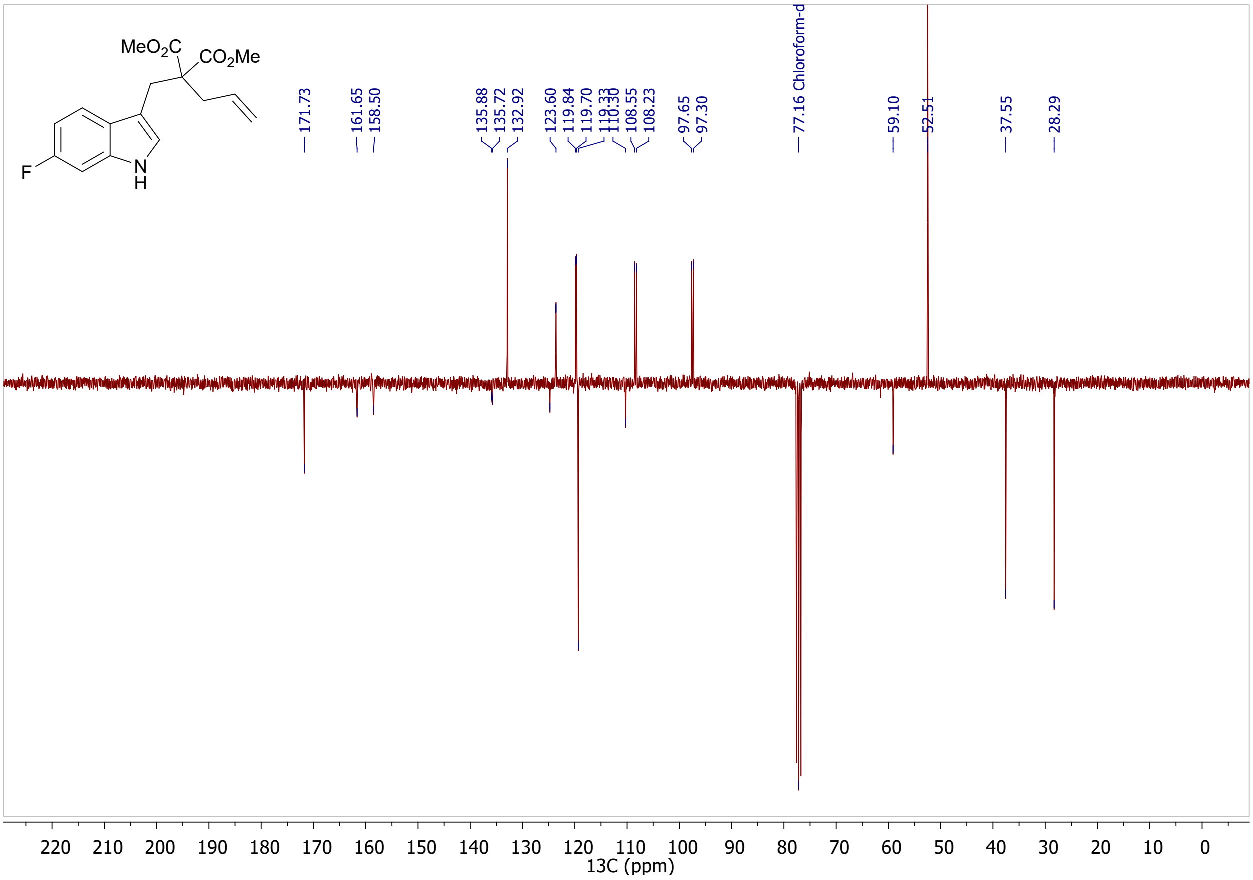

^{19}F NMR (377 MHz, $CDCl_3$) of **3e**

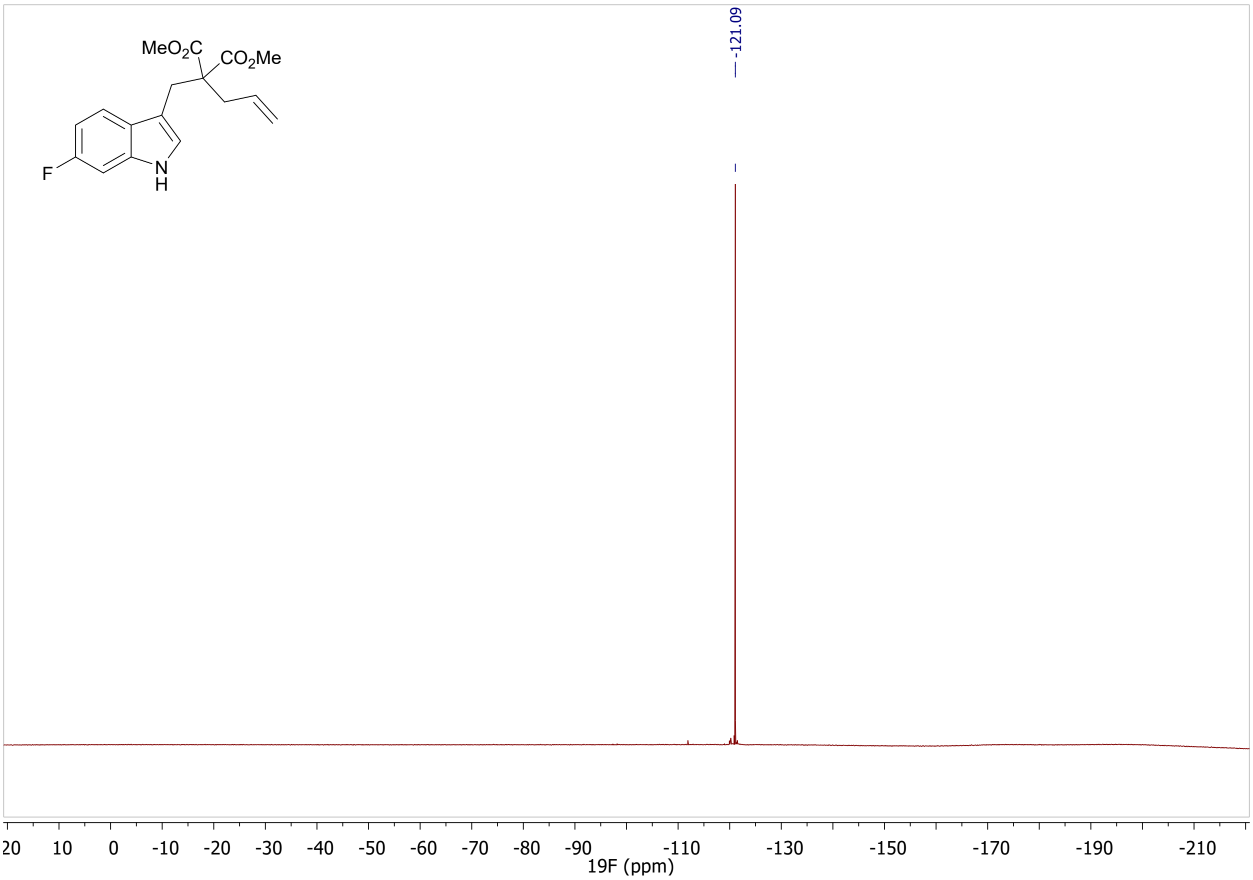

^1H NMR (300 MHz, $CDCl_3$) of **3g**

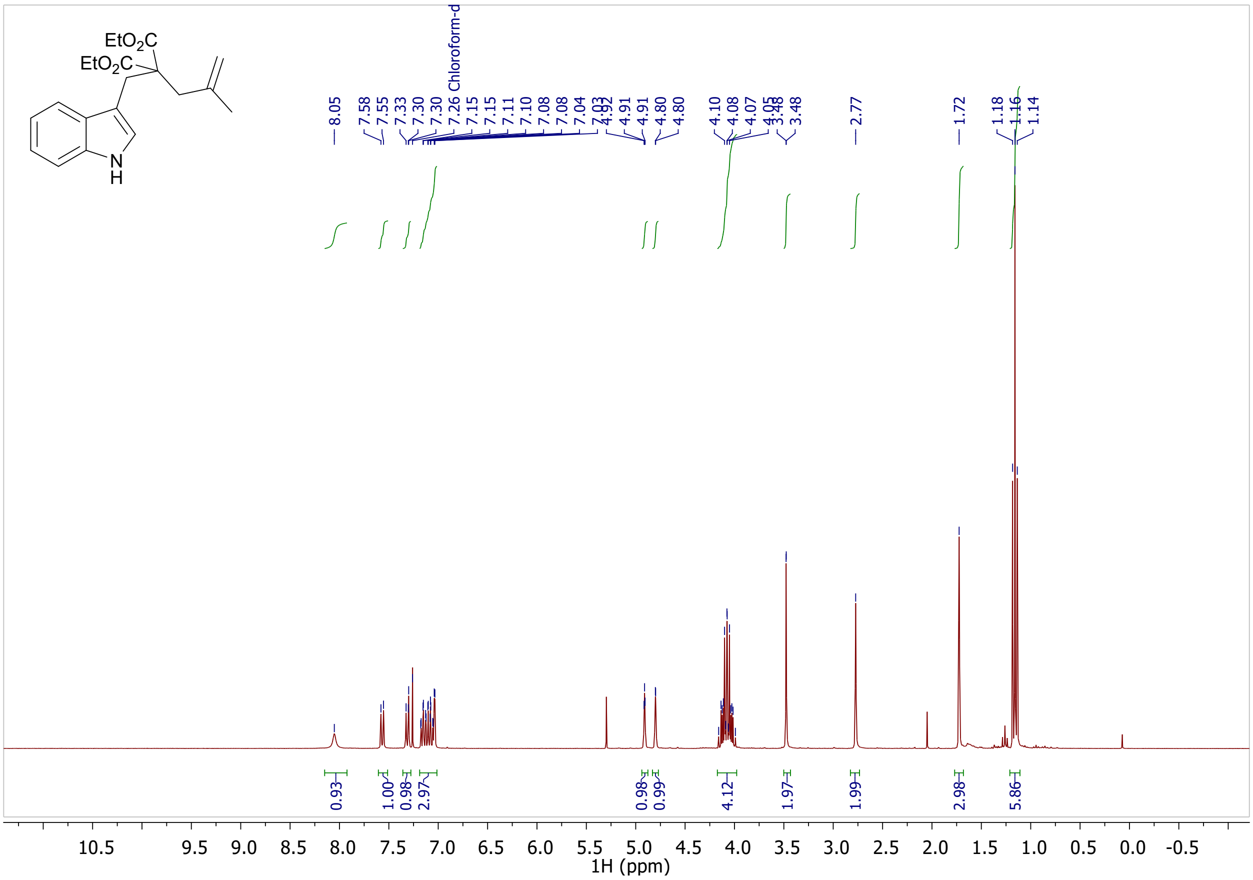

^1H NMR (300 MHz, $CDCl_3$) of **3i**

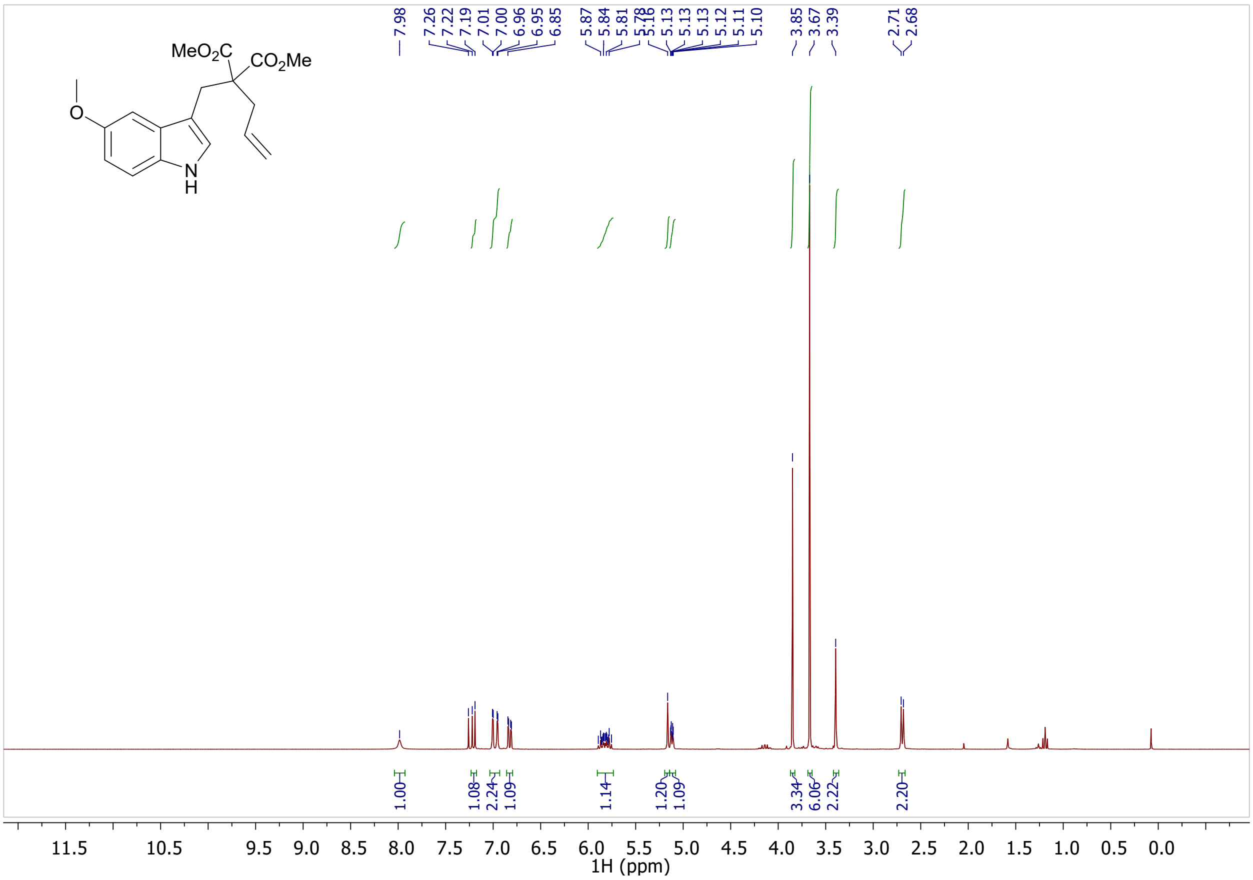

^{13}C NMR (75 MHz, $CDCl_3$) of **3i**

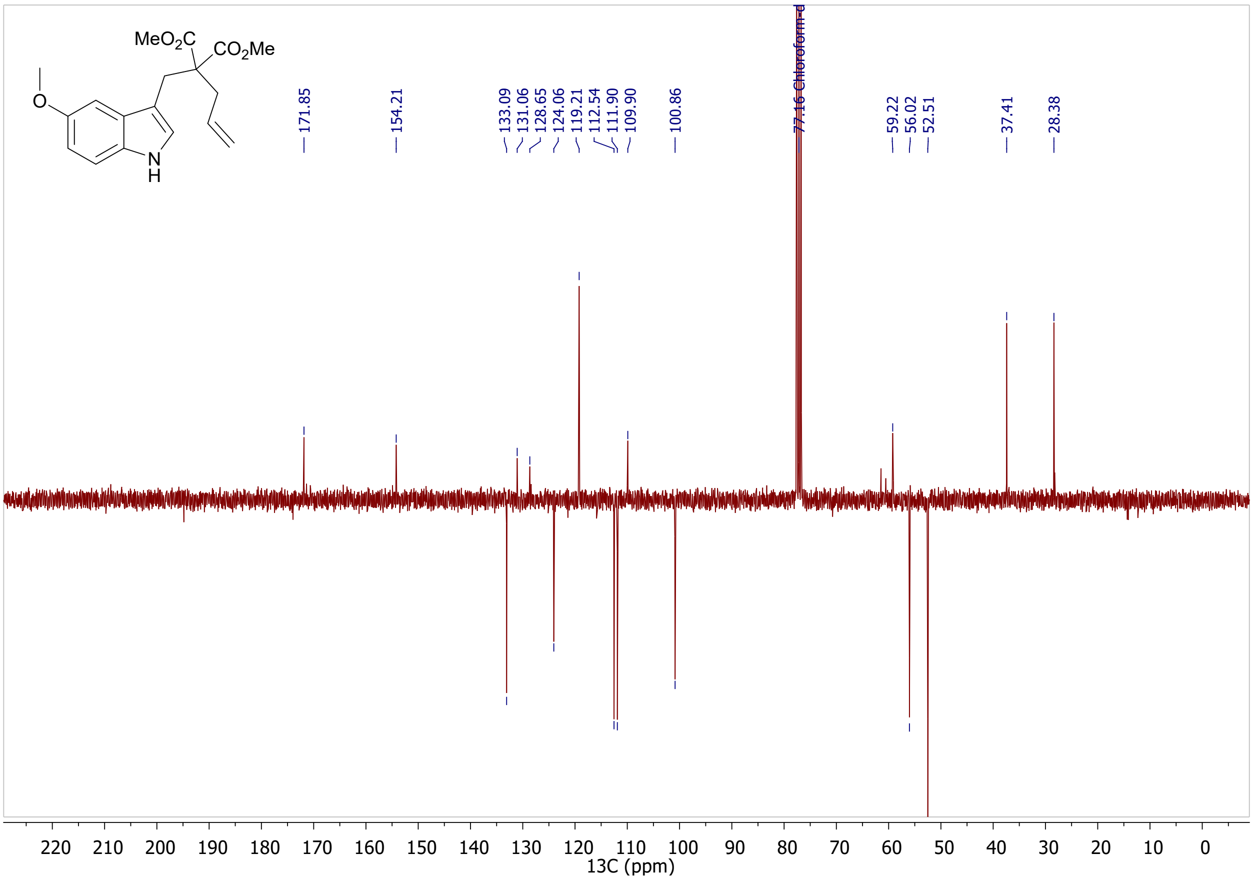

^{1}H NMR (300 MHz, $CDCl_3$) of **3j**

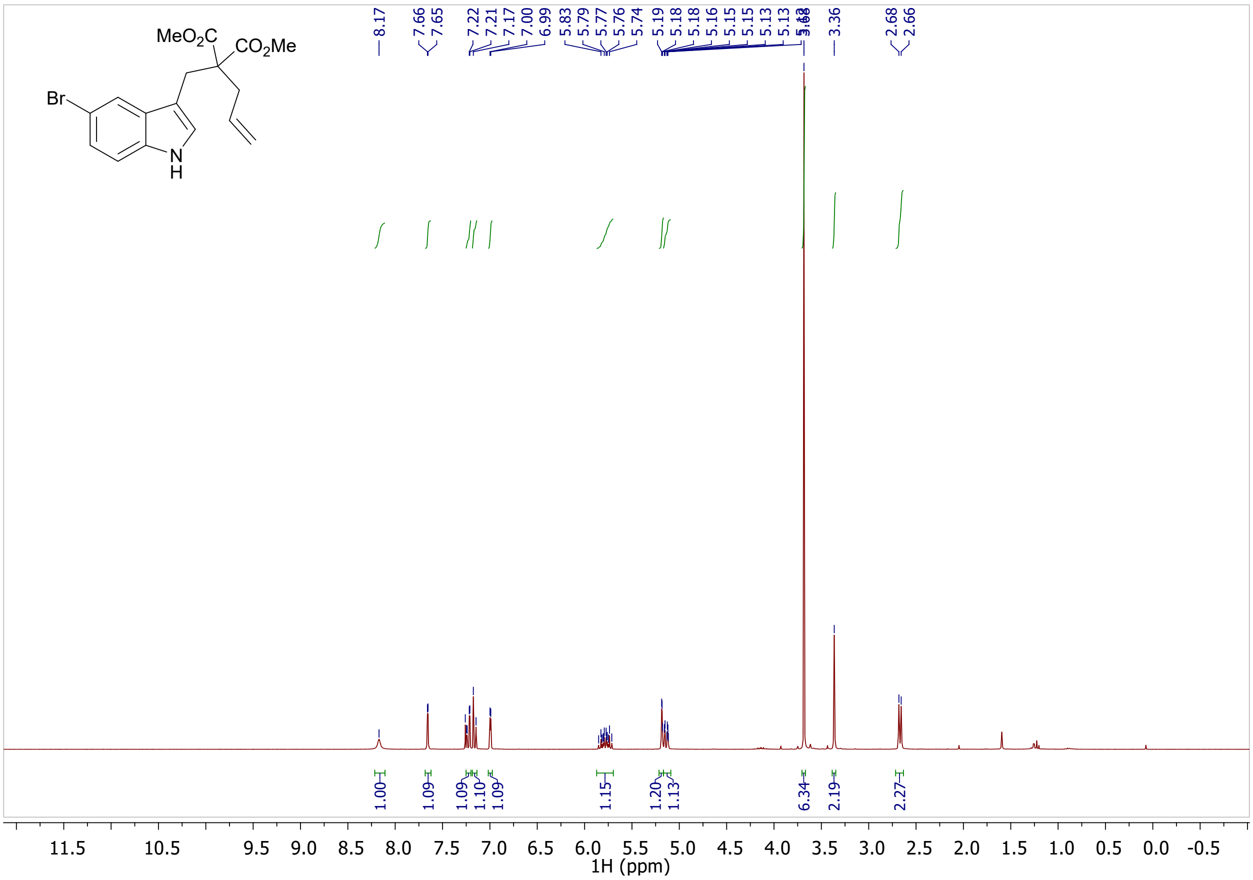

^{13}C NMR (75 MHz, $CDCl_3$) of **3j**

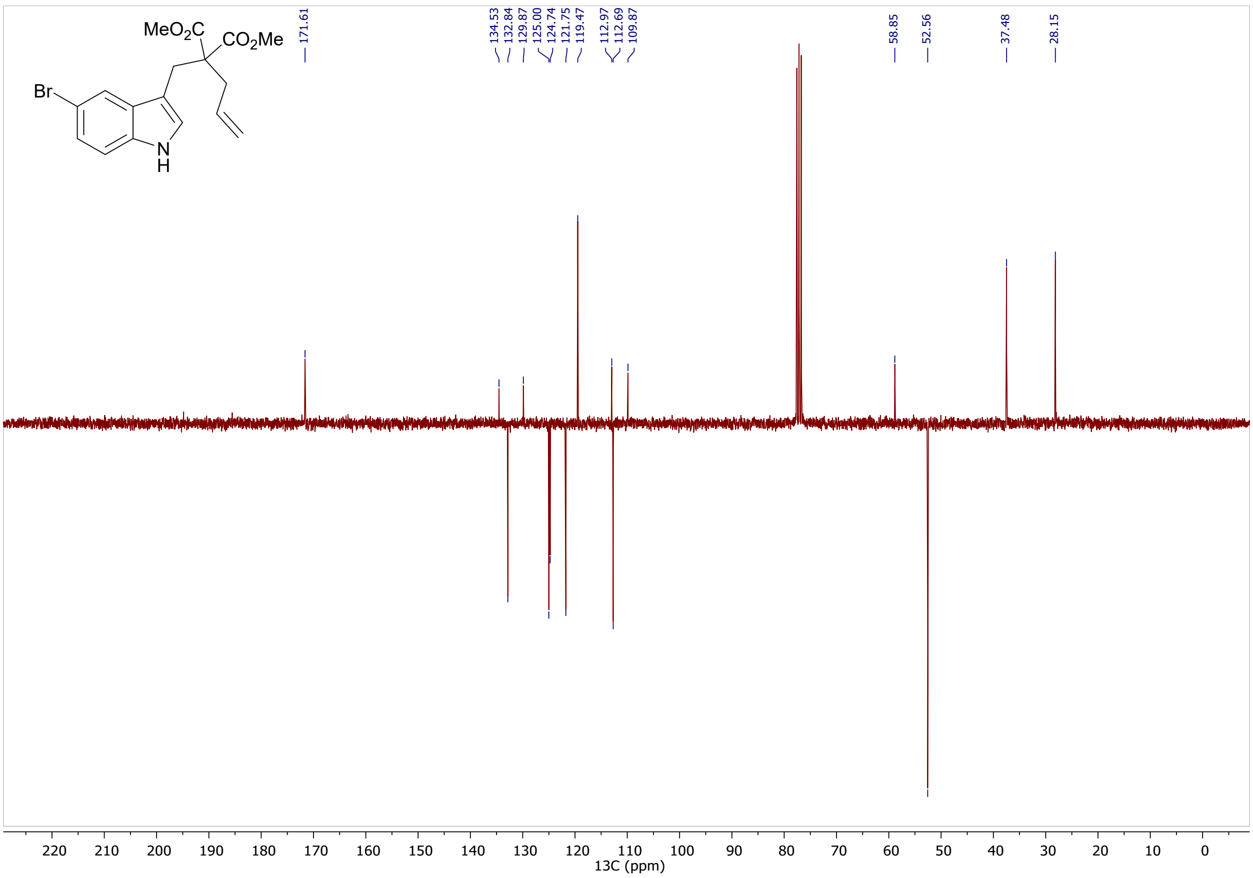

^{1}H NMR (300 MHz, $CDCl_3$) of **3k**

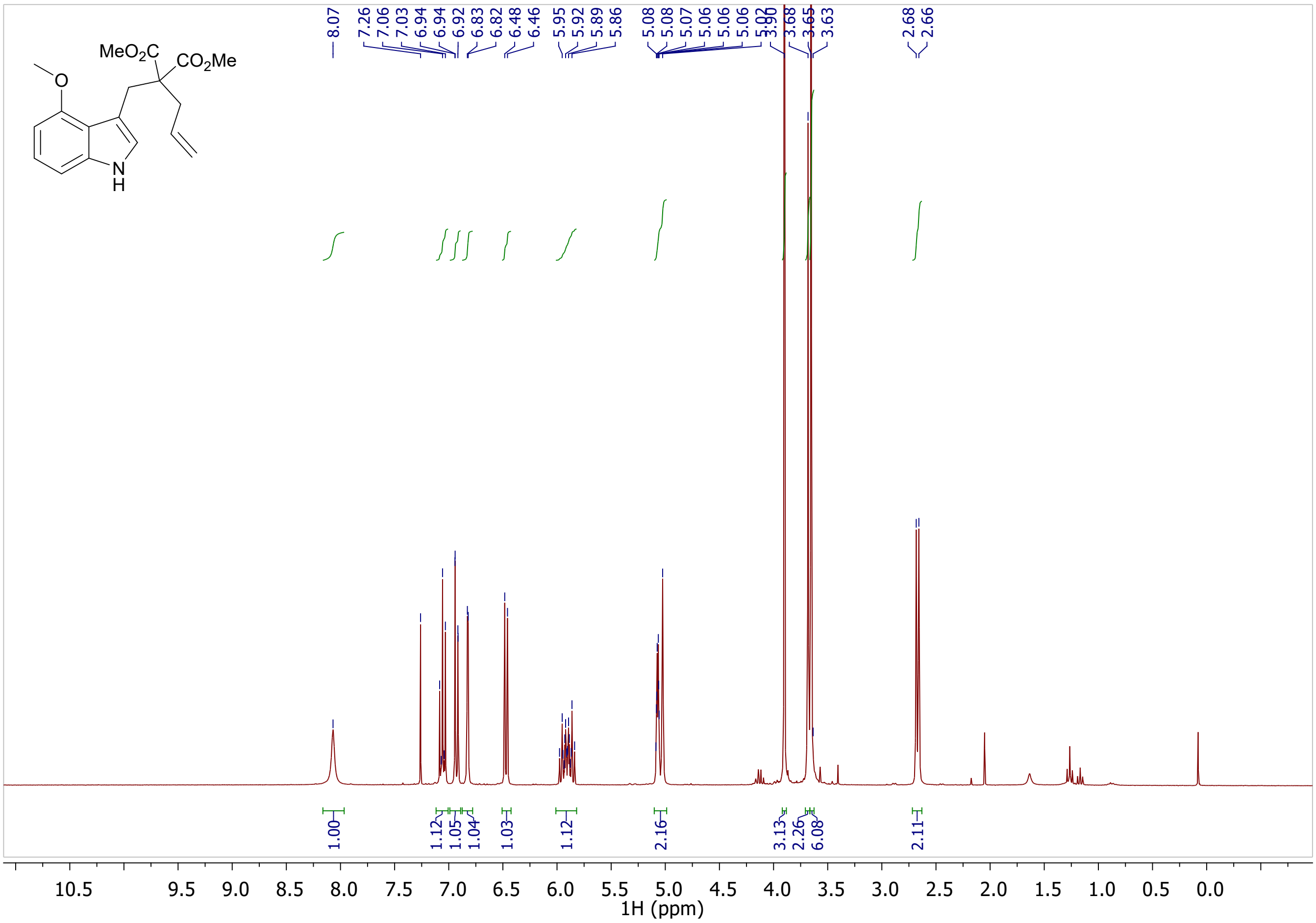

^{13}C NMR (75 MHz, $CDCl_3$) of **3k**

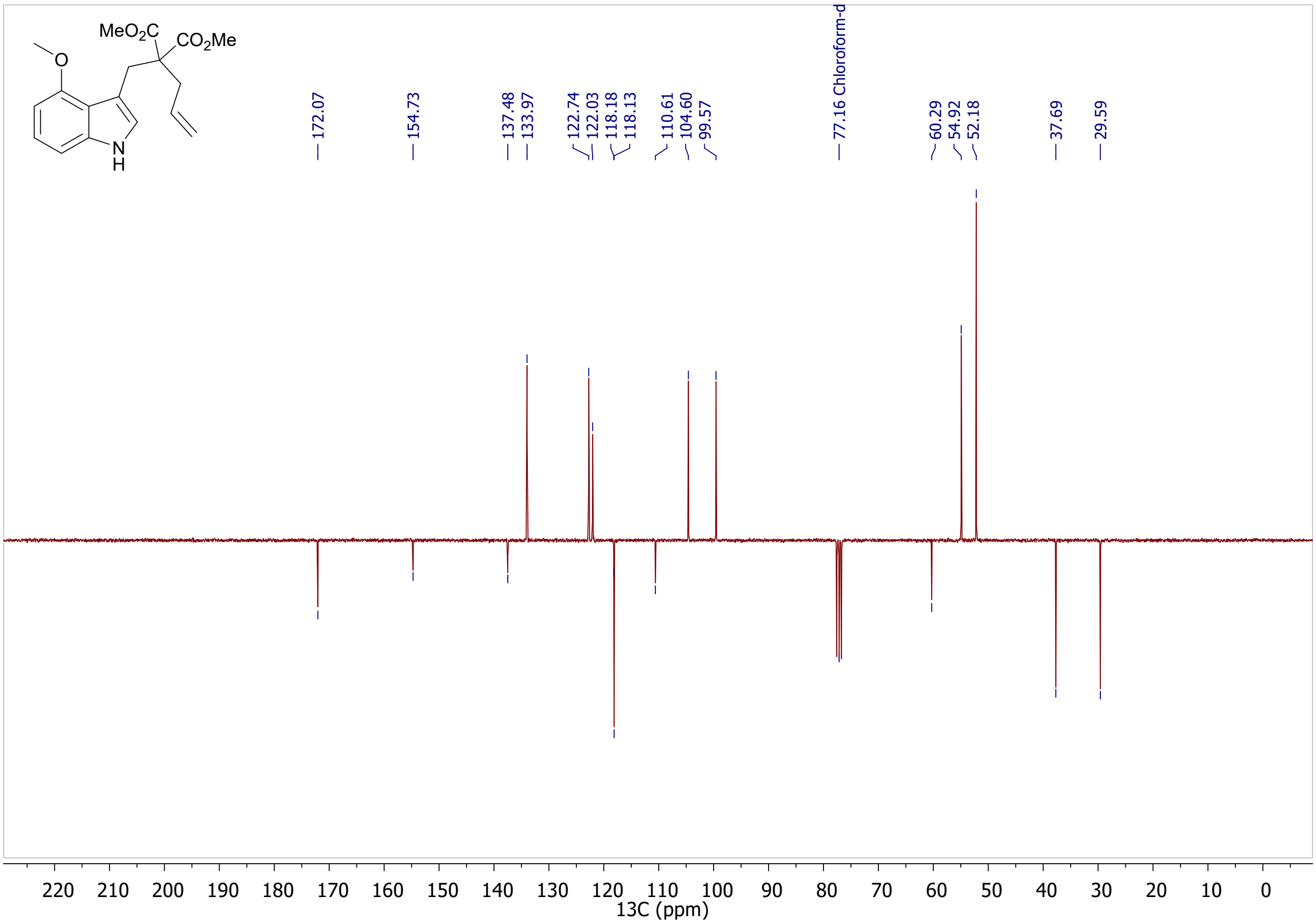

^{1}H NMR (300 MHz, $CDCl_3$) of (1R*,5S*,6R*,7S*) Diastereomer (major product) of **2a:**

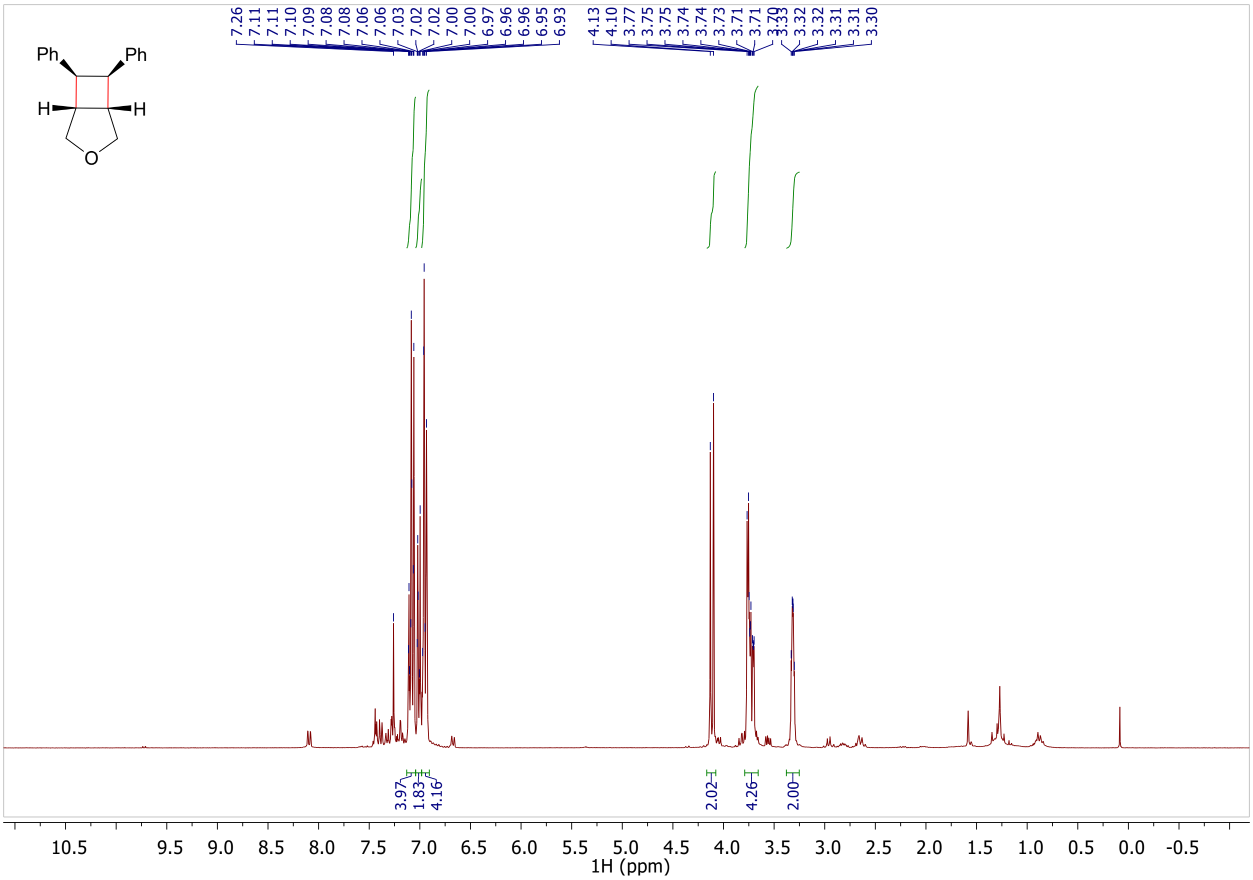

^{1}H NMR (300 MHz, $CDCl_3$) of (1R*,5S*,6S*,7S*) Diastereomer (minor product) of **2a:**

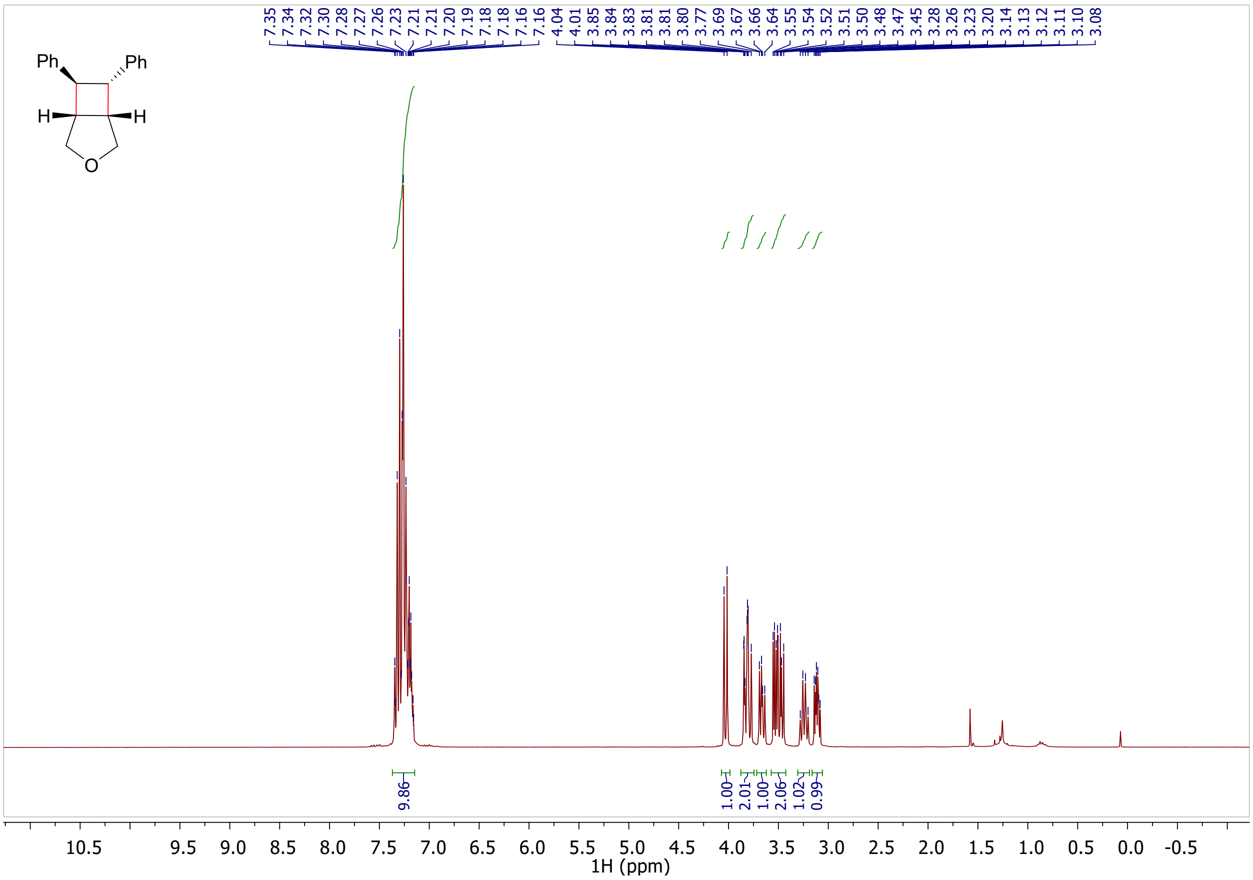

^{1}H NMR (300 MHz, $CDCl_3$) of (1S*,5R*,6S*)-Diastereomer (major product): **2b:**

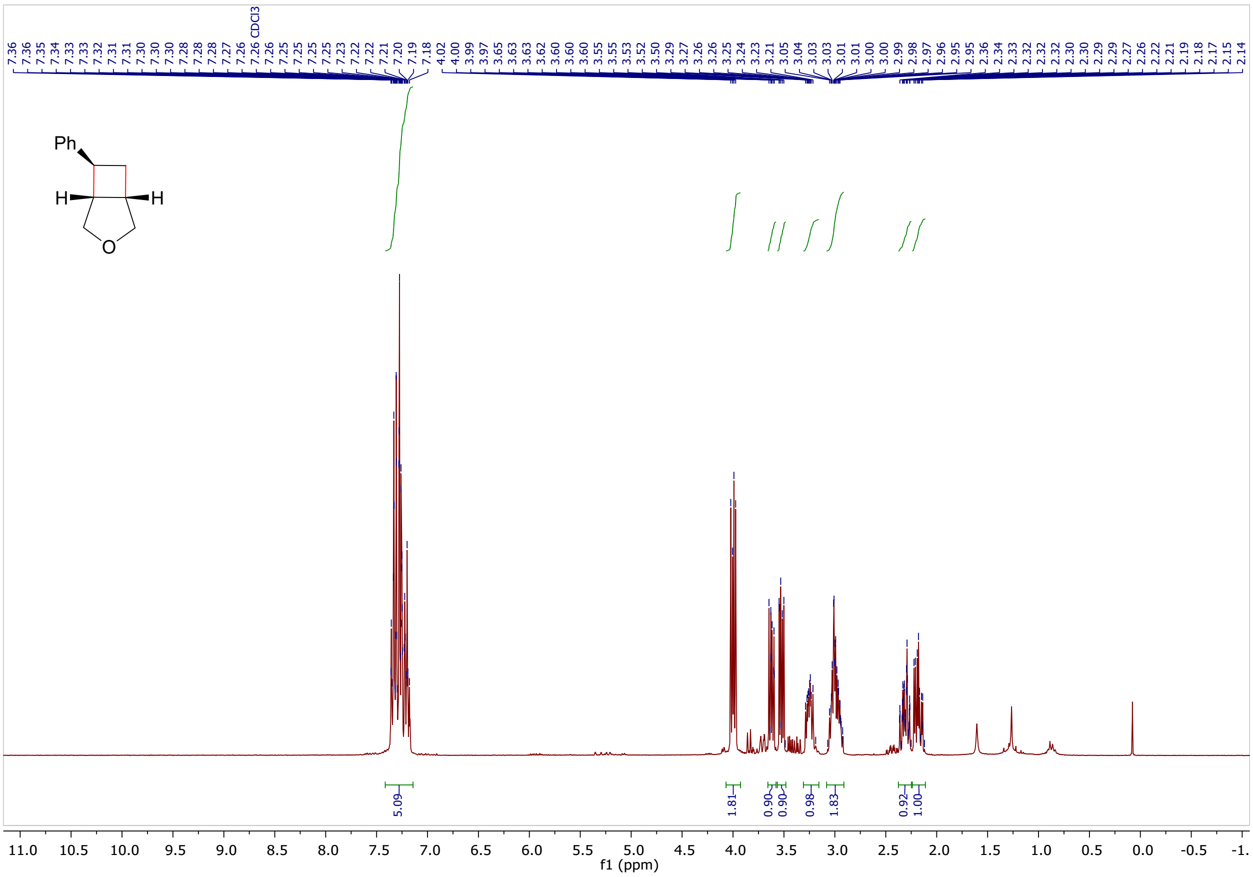

^{1}H NMR (300 MHz, $CDCl_3$) of (1R*,5R*,7R*)-Diastereomer (major product) **2c:**

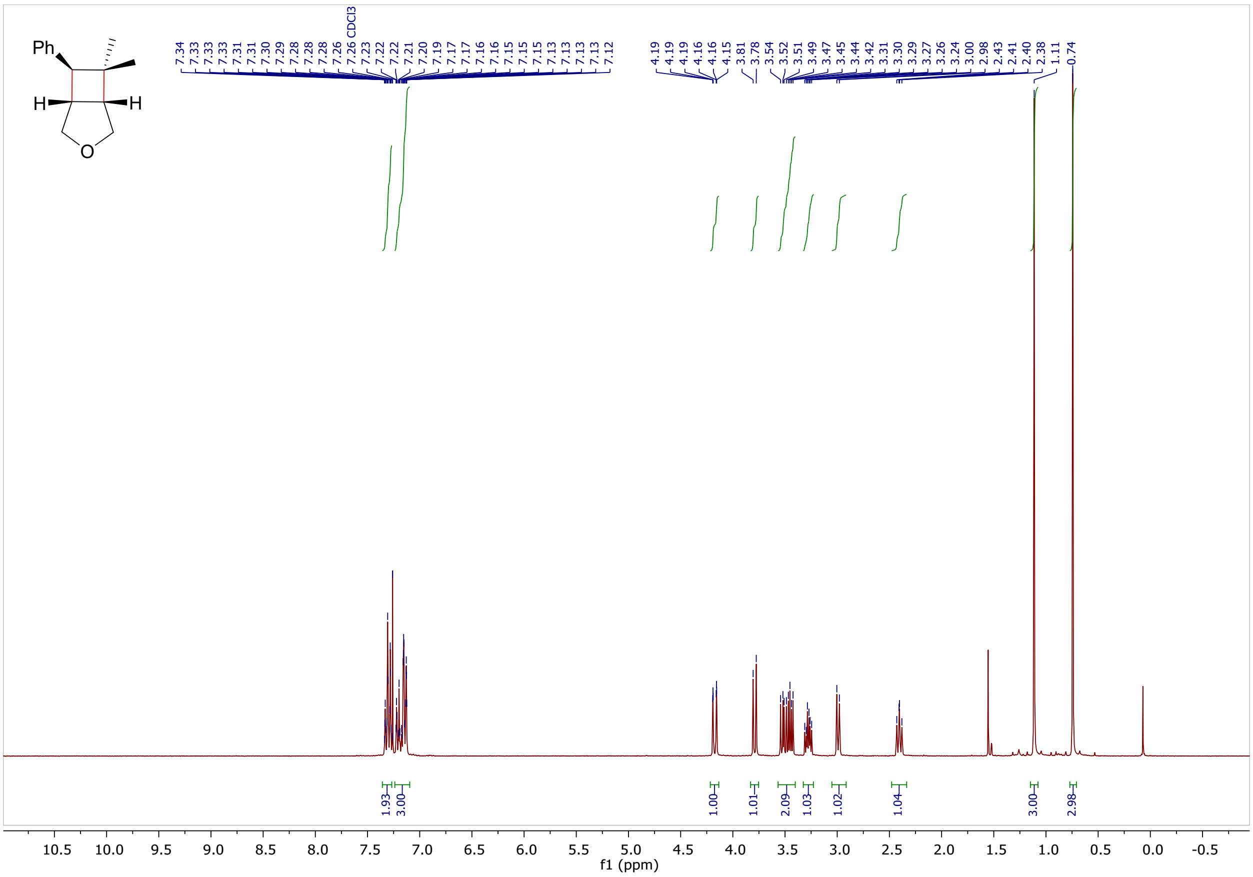

^{1}H NMR (300 MHz, $CDCl_3$) of (1S*,5R*,6S*) Diastereomer (major product) **2d:**

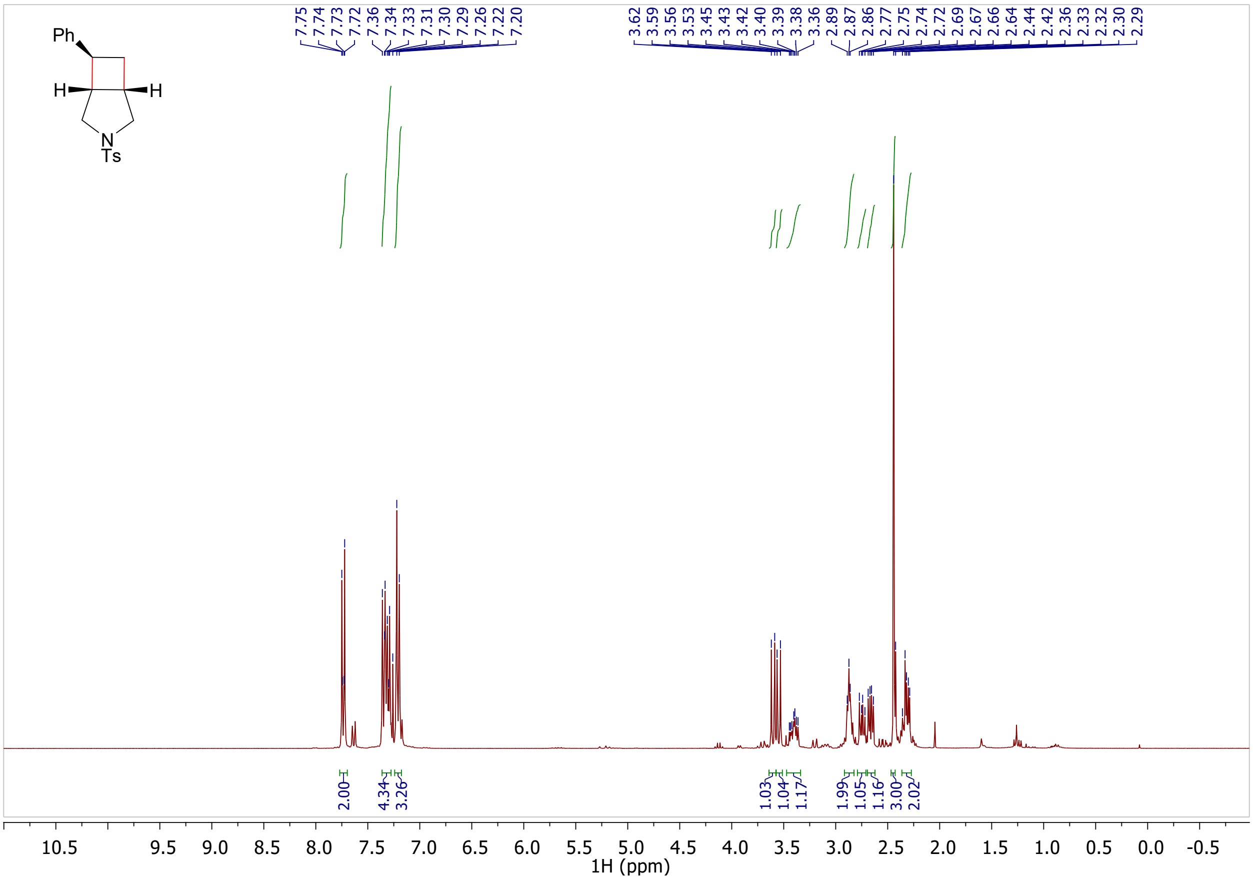

^{1}H NMR (300 MHz, $CDCl_3$) of (1S*,5R*,7R*) Diastereomer (major product) **2e:**

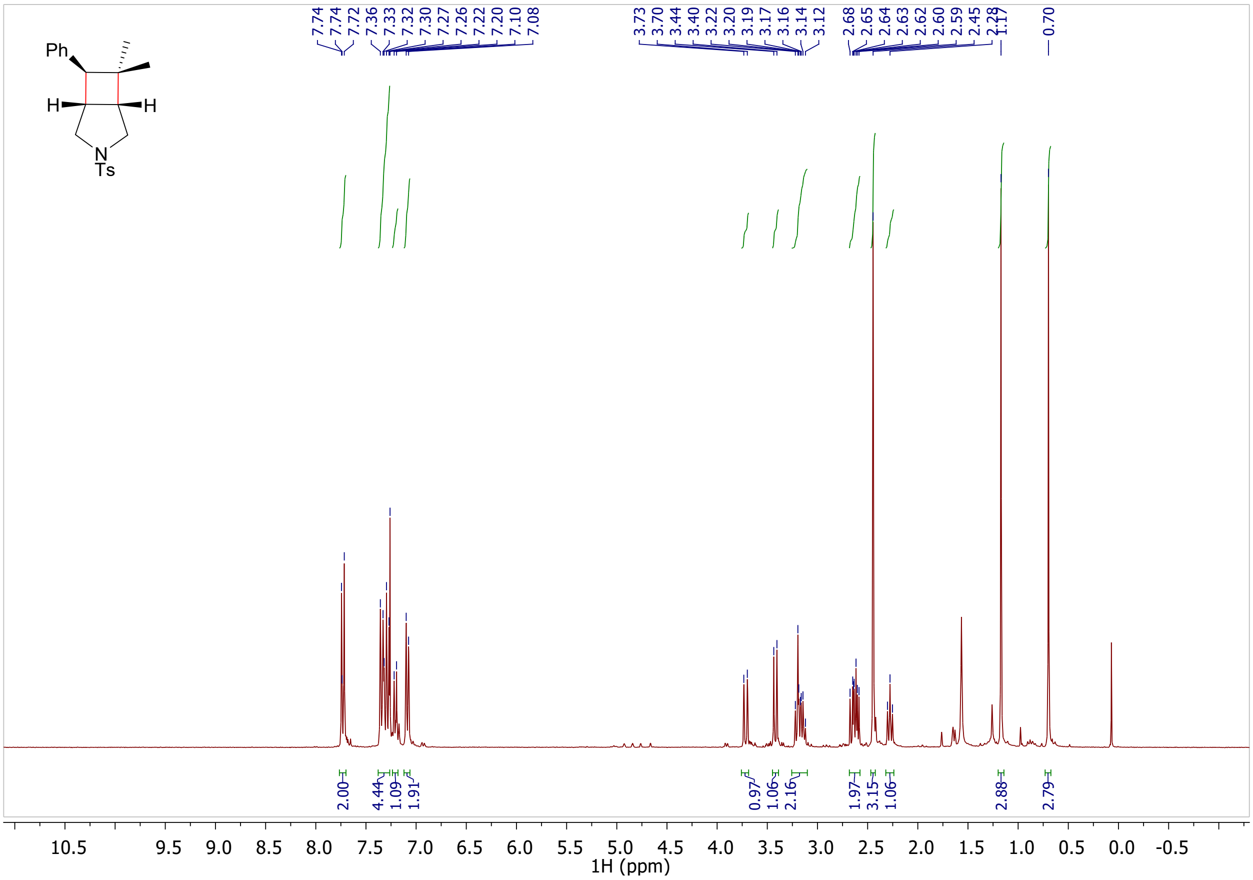

^{1}H NMR (300 MHz, $CDCl_3$) of (1R*, 5S*, 7R*) Diastereomer (major product) **2f:**

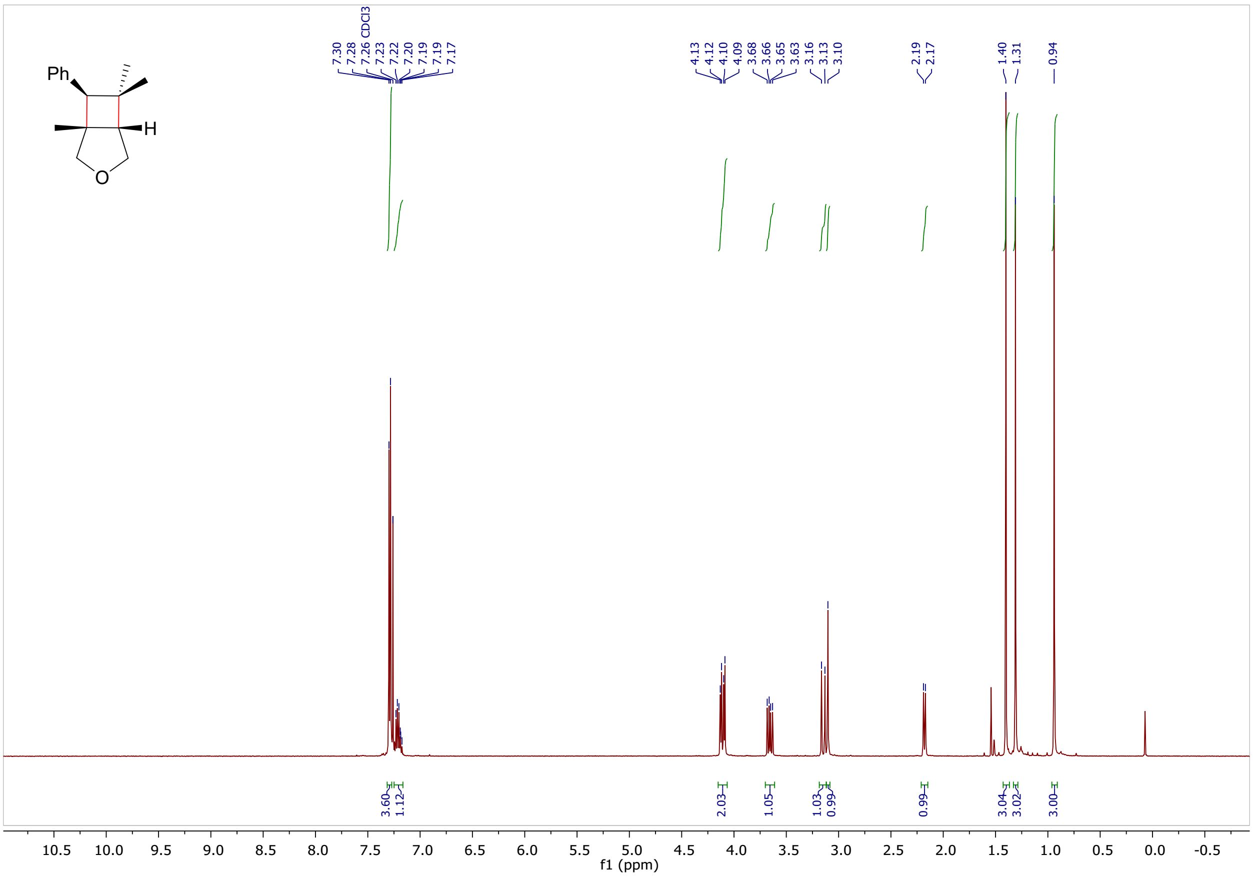

^{1}H NMR (300 MHz, $CDCl_3$) of (1R*, 5S*, 7S*) Diastereomer (minor product) **2f:**

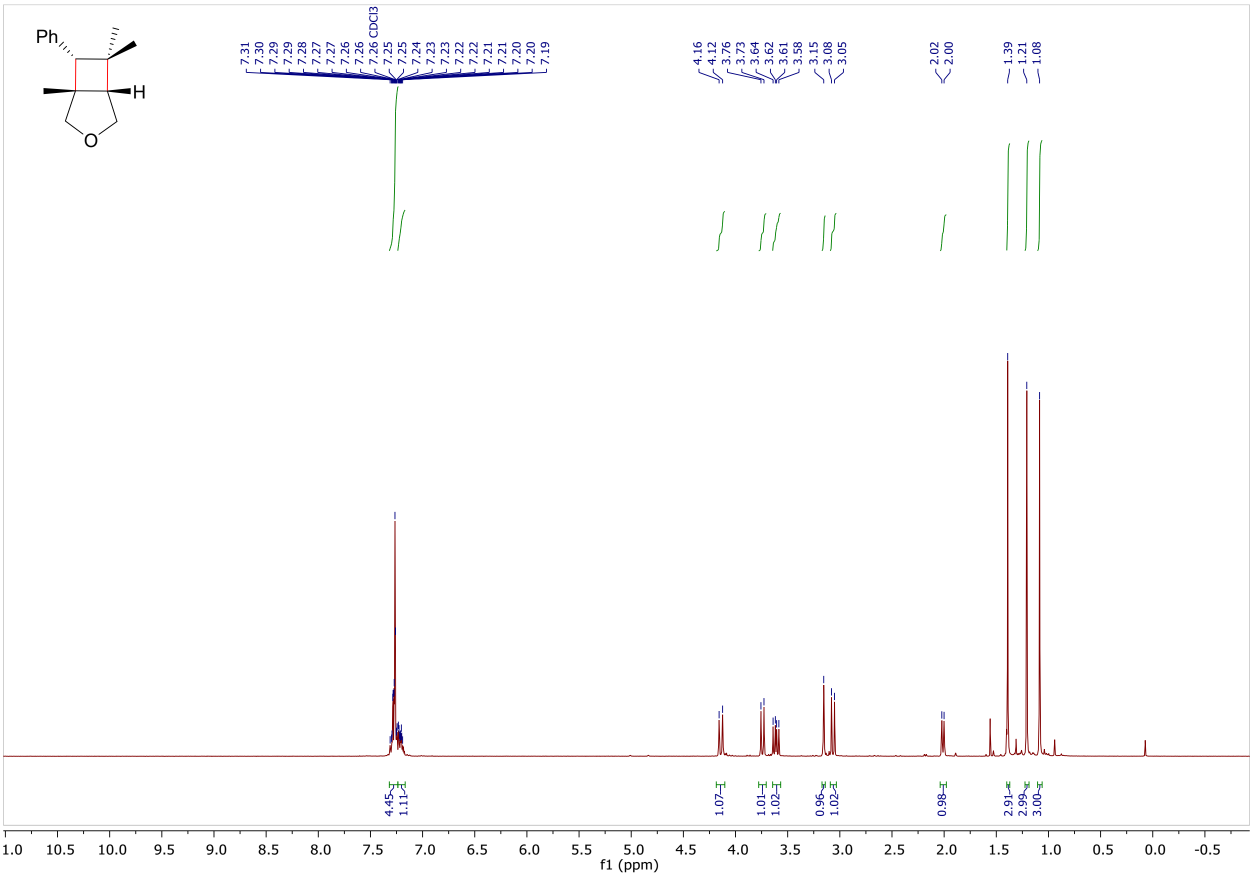

^{1}H NMR (300 MHz, $CDCl_3$) of (1S*,2S*,5R*,6S*) Diastereomer (major product) **2g:**

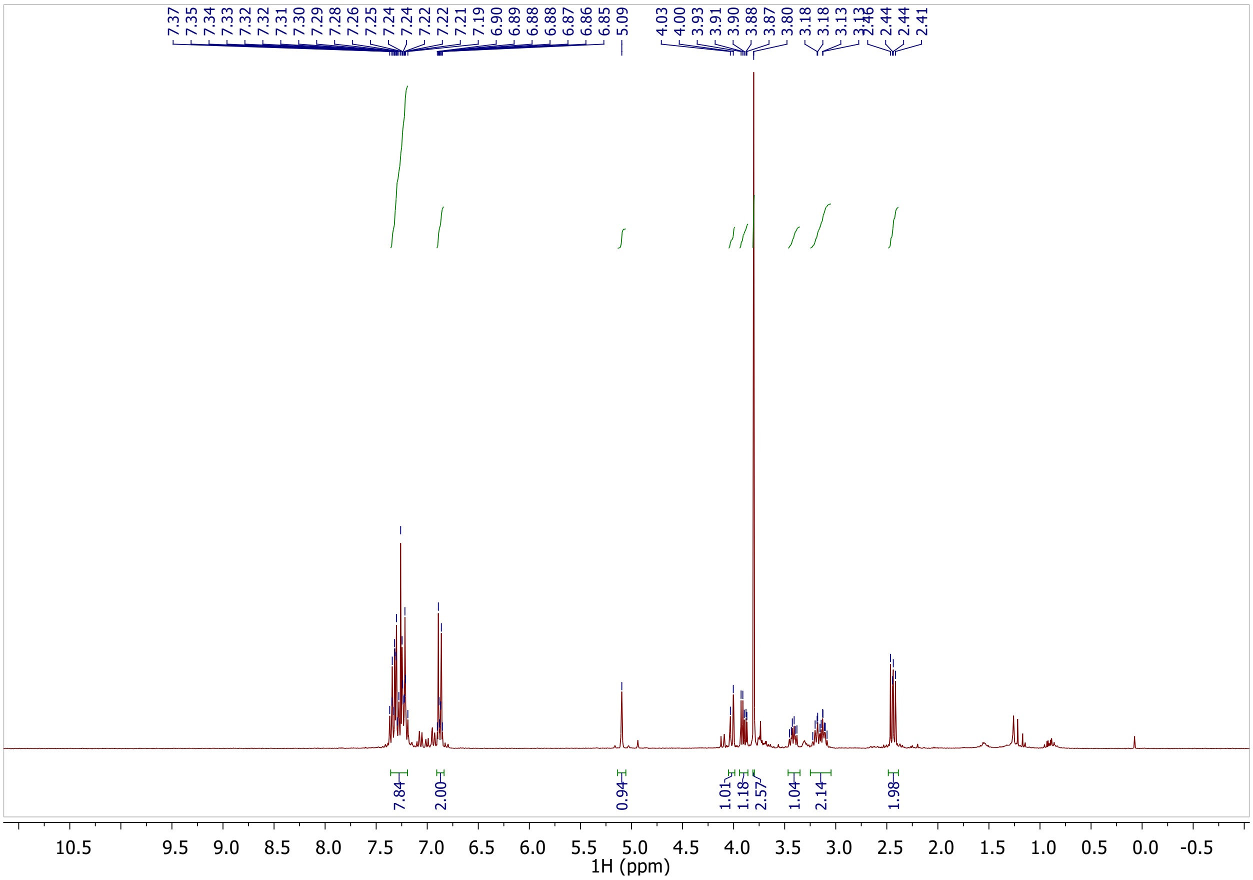

^{1}H NMR (300 MHz, $CDCl_3$) of (1S*, 5R*, 7R*) Diastereomer (major product) **2h:**

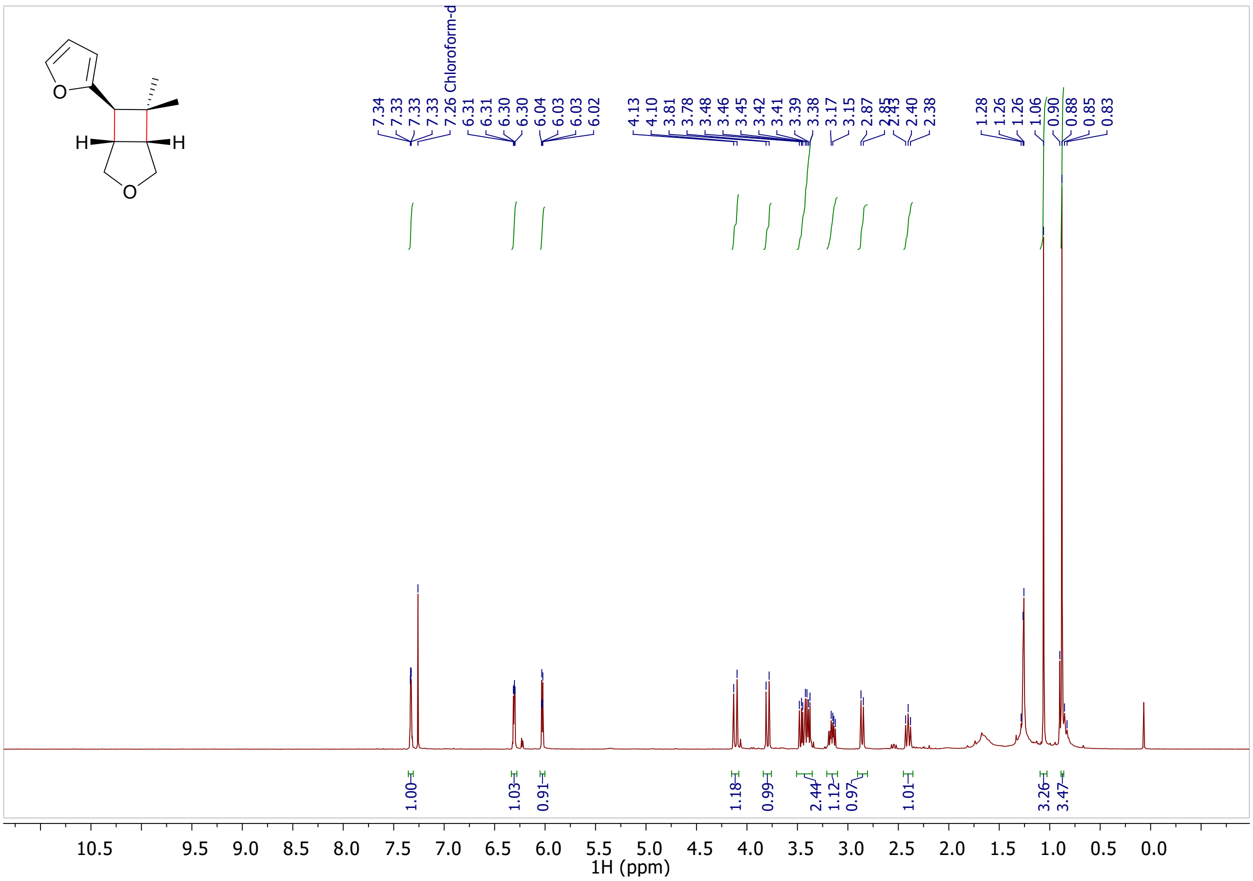

^{1}H NMR (300 MHz, $CDCl_3$) of (1R*,5R*,7R*)-Diastereomer (major product) **2i:**

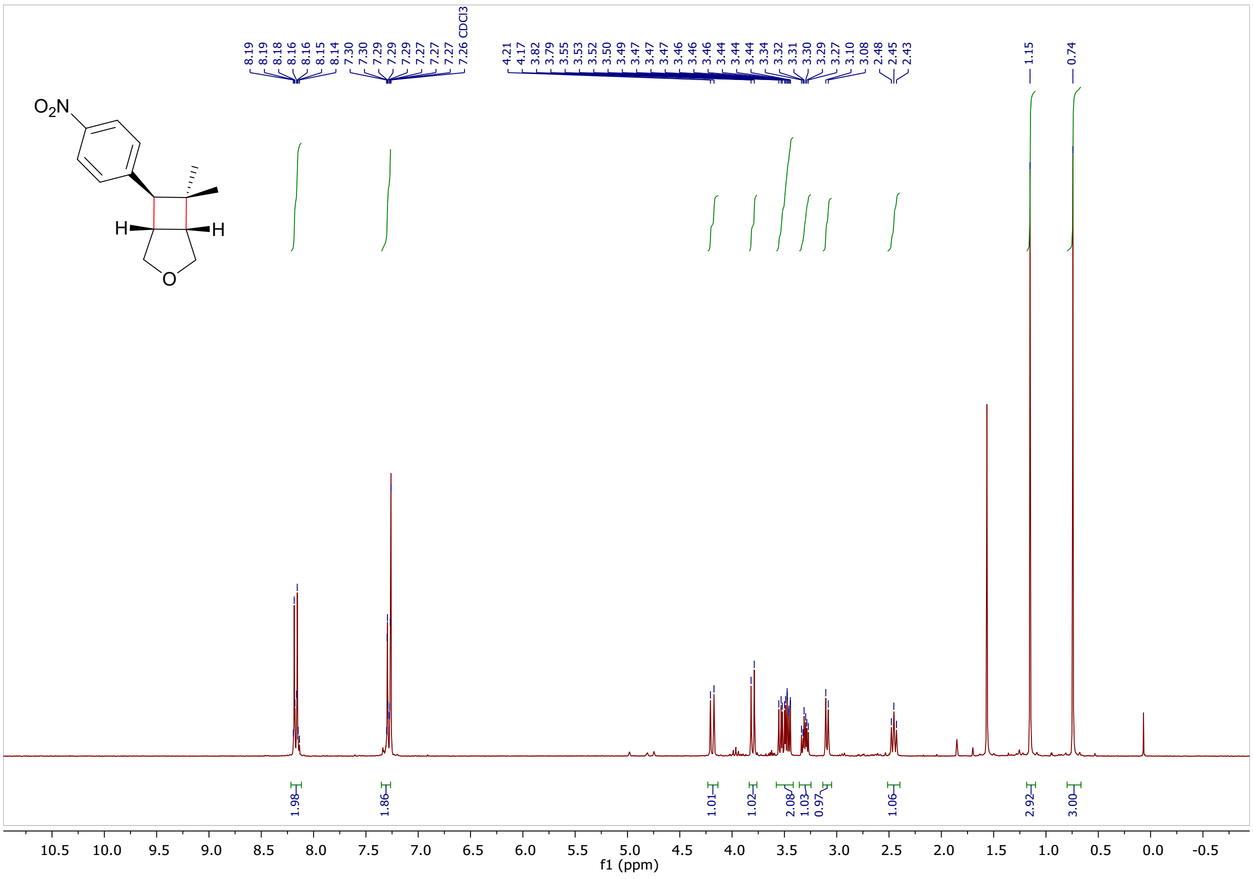

^{1}H NMR (300 MHz, $CDCl_3$) of (1R*,5R*,7S*)-Diastereomer (major product) **2j:**

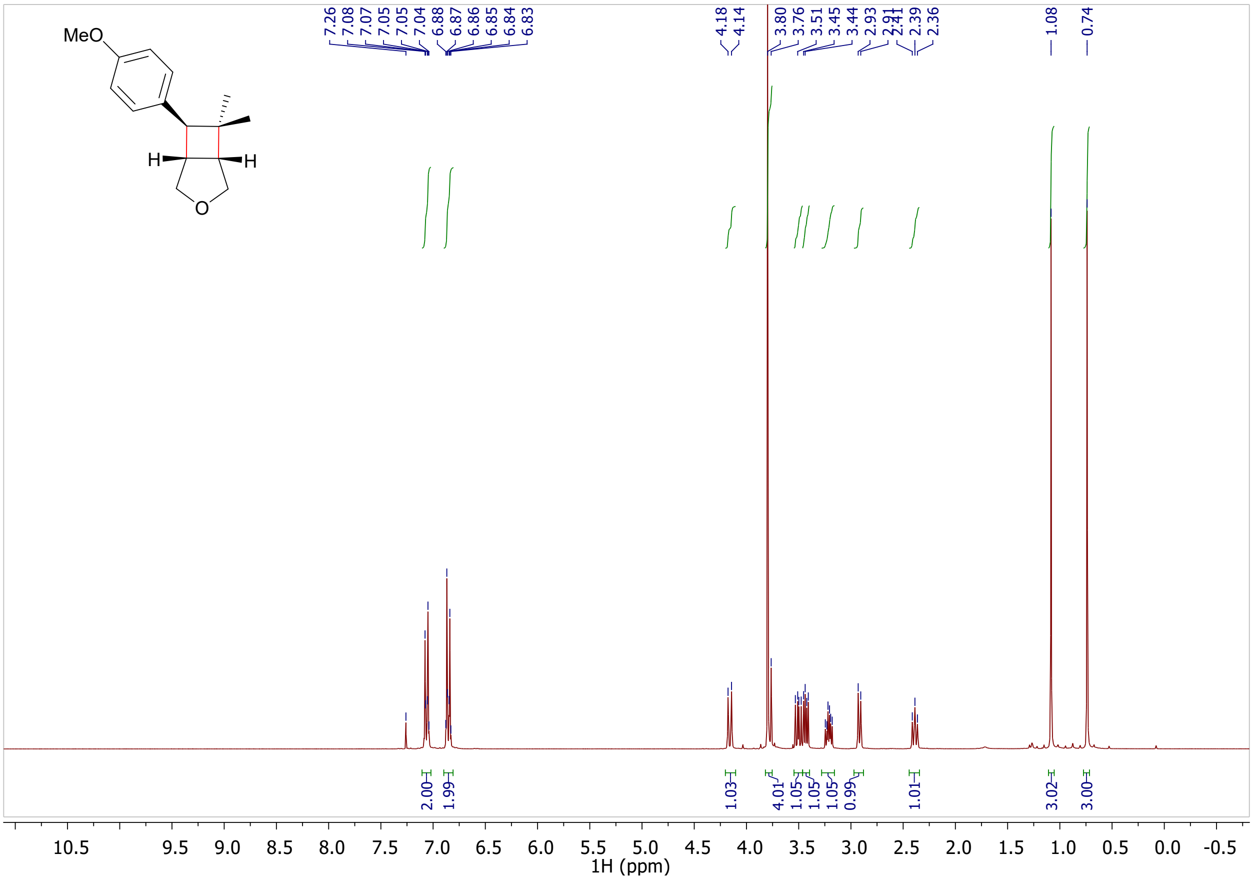

^{1}H NMR (400 MHz, $CDCl_3$) of **4a**

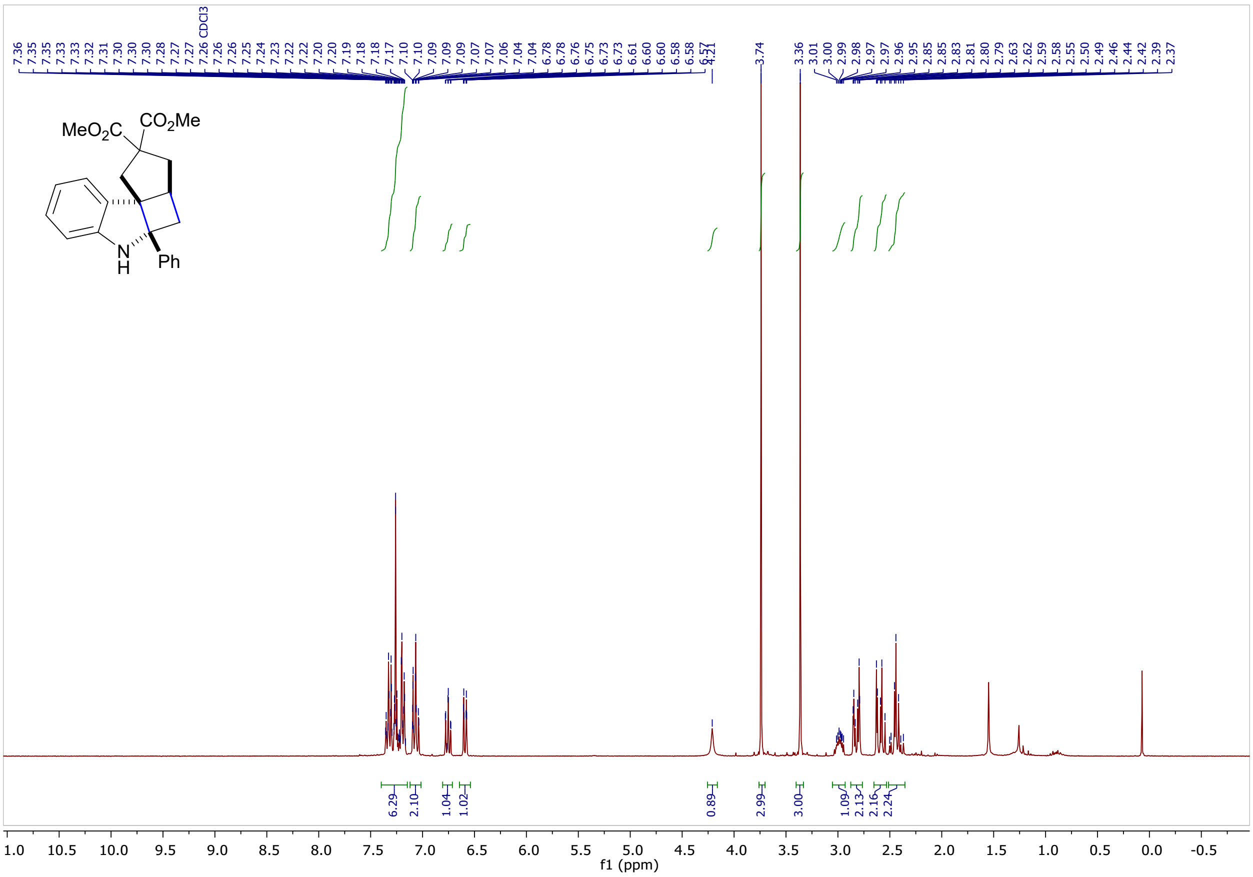

^{1}H NMR (300 MHz, $CDCl_3$) of **4b**

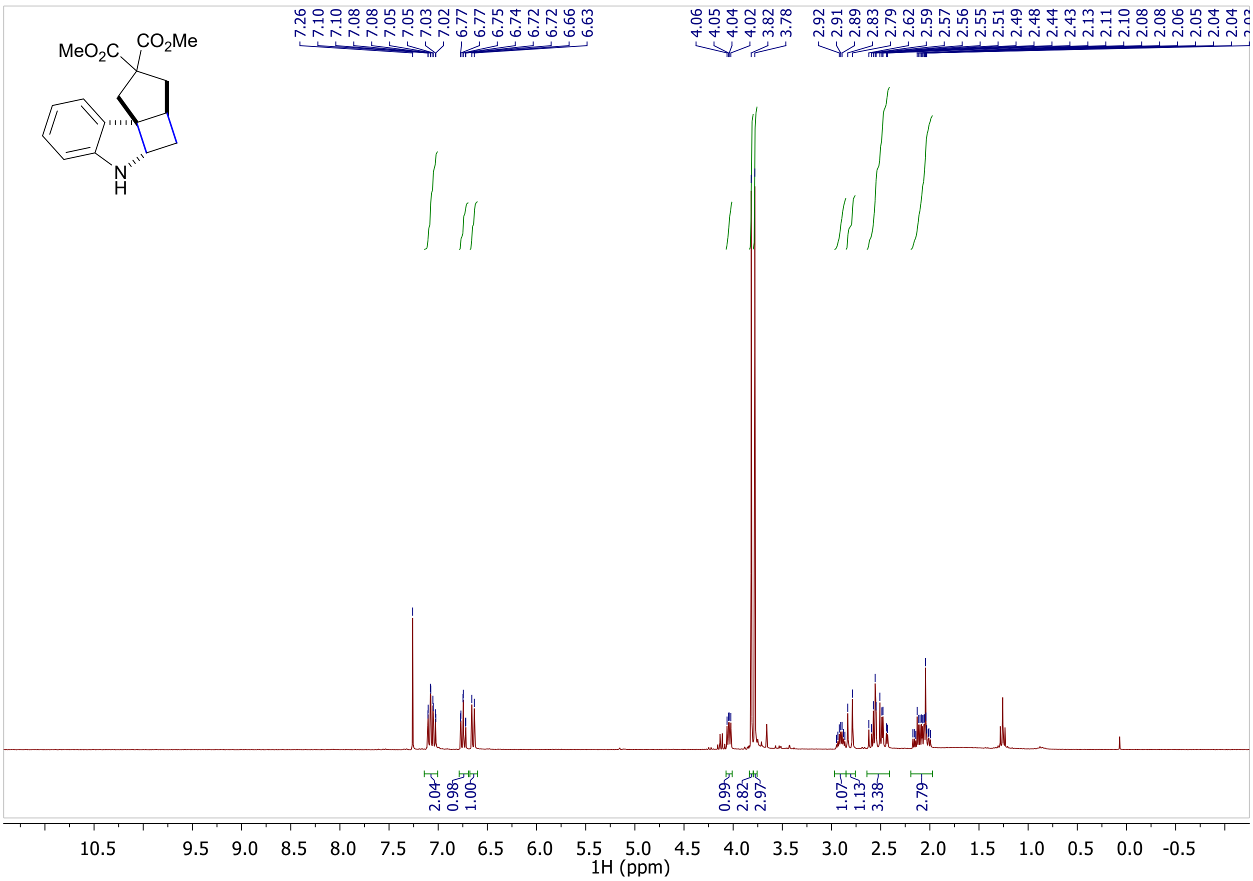

^{13}C NMR (75 MHz, $CDCl_3$) of **4b**

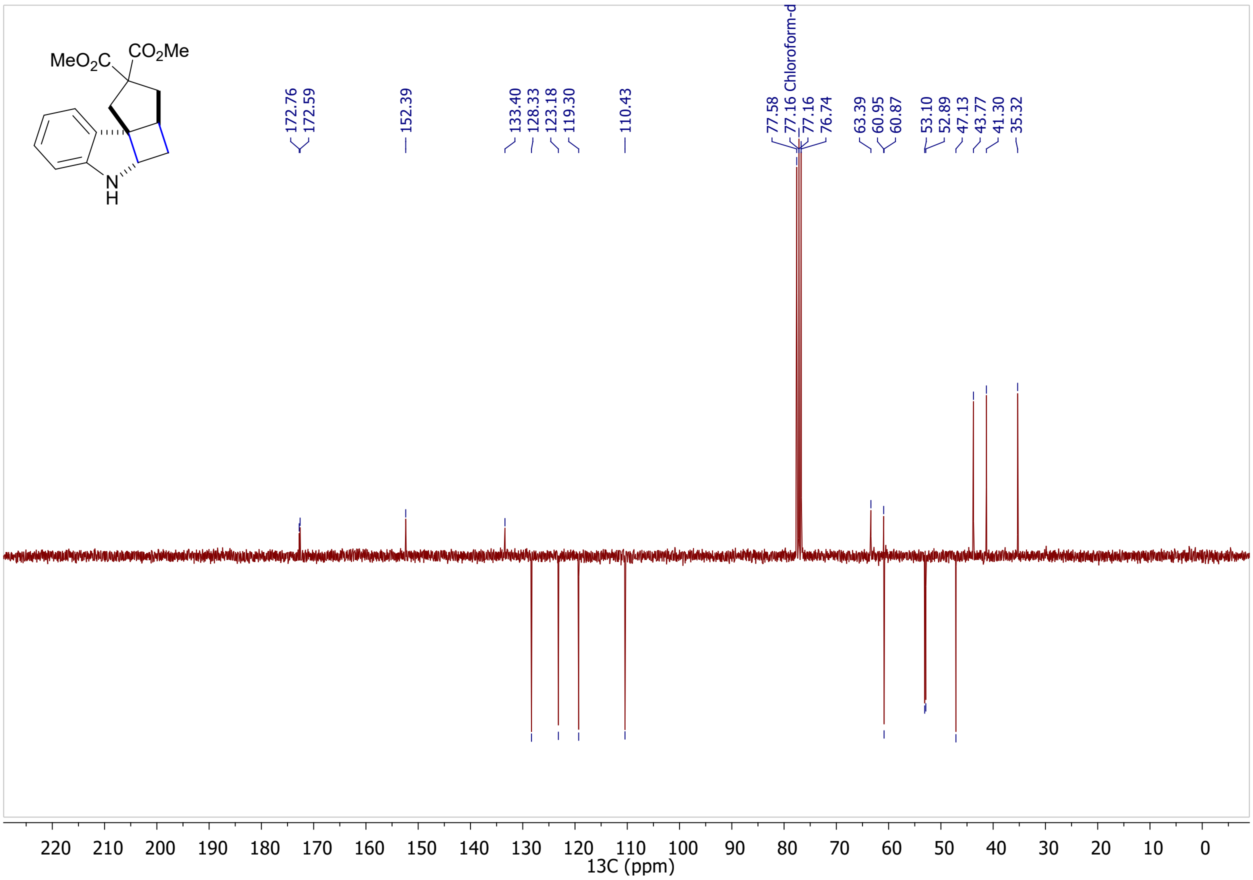

^1H NMR (300 MHz, $CDCl_3$) of **4c**

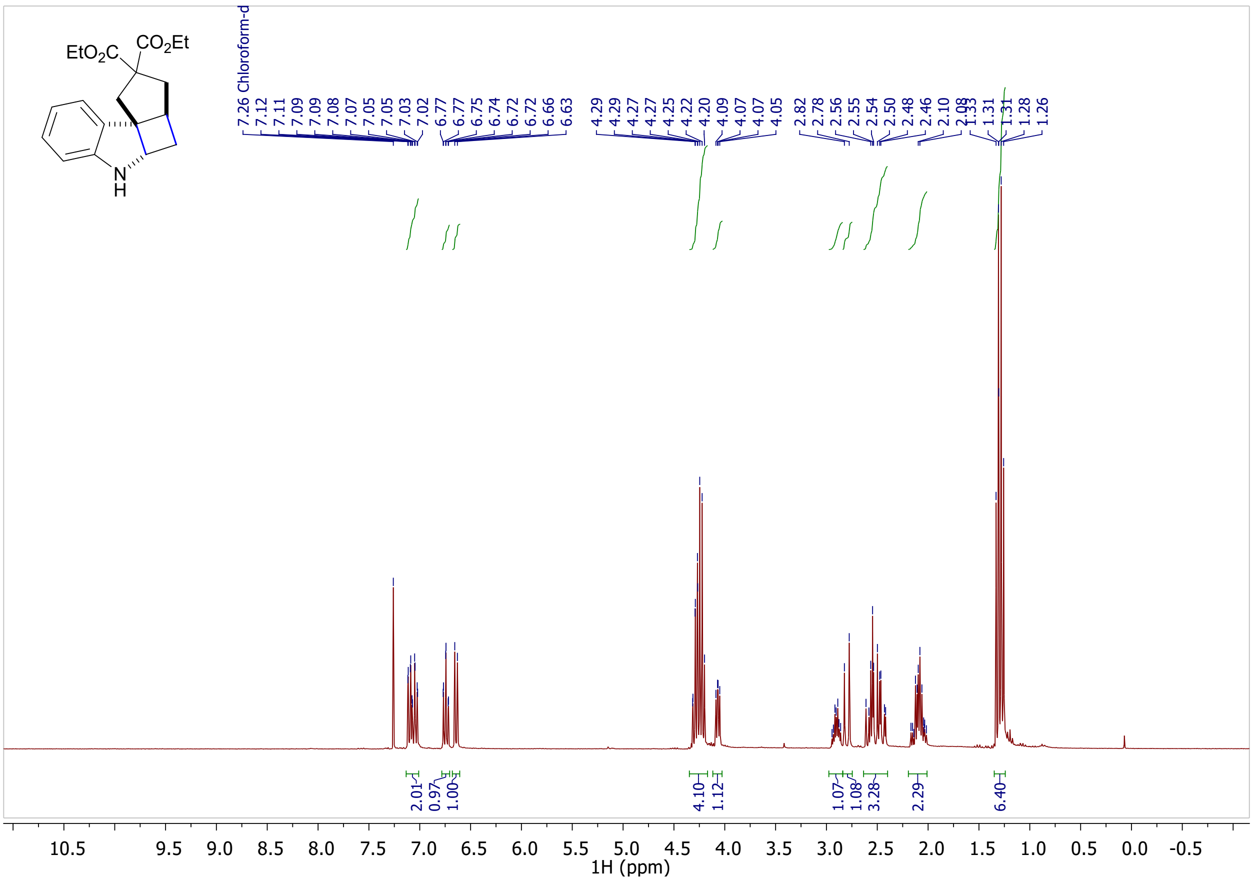

^{13}C NMR (75 MHz, $CDCl_3$) of **4c**

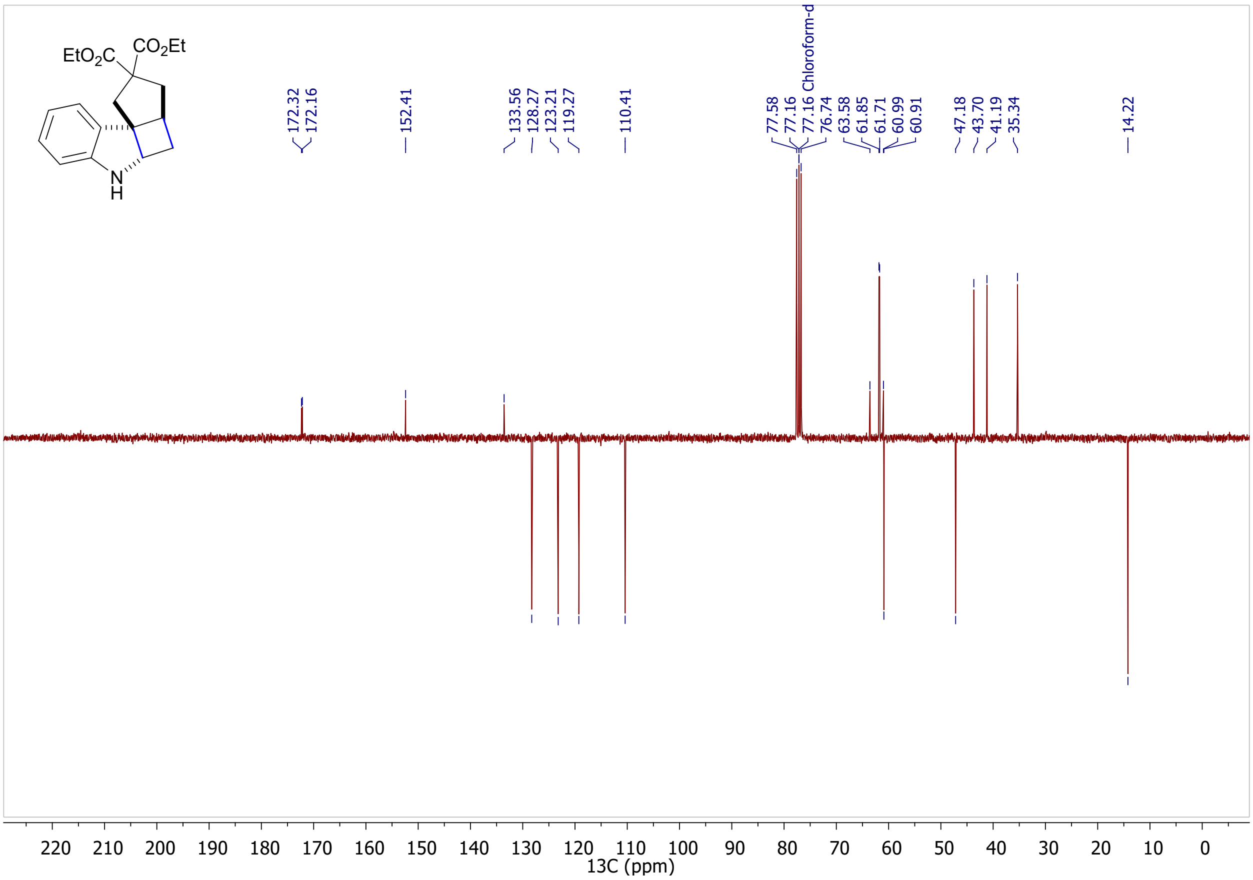

^{1}H NMR (300 MHz, $CDCl_3$) of **4d**

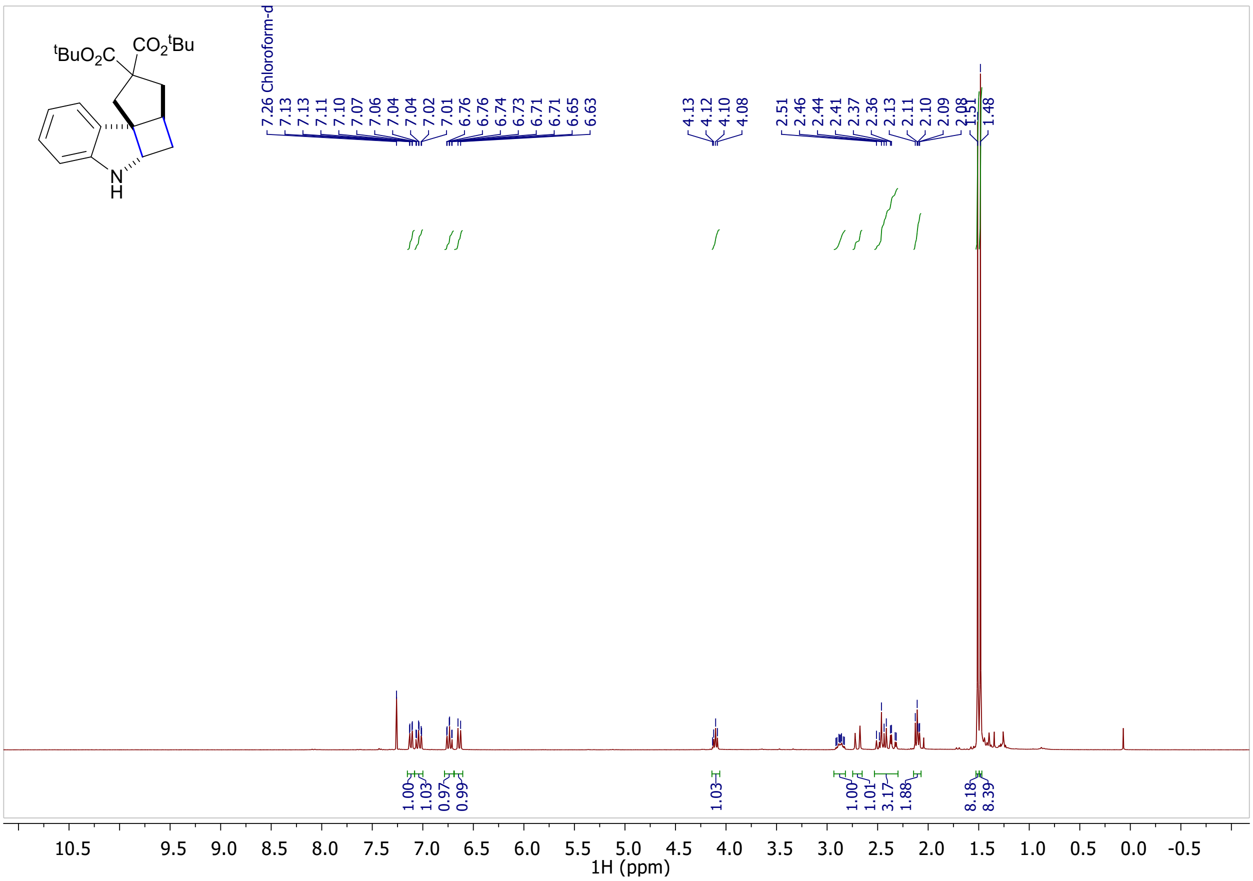

^{13}C NMR (75 MHz, $CDCl_3$) of **4d**

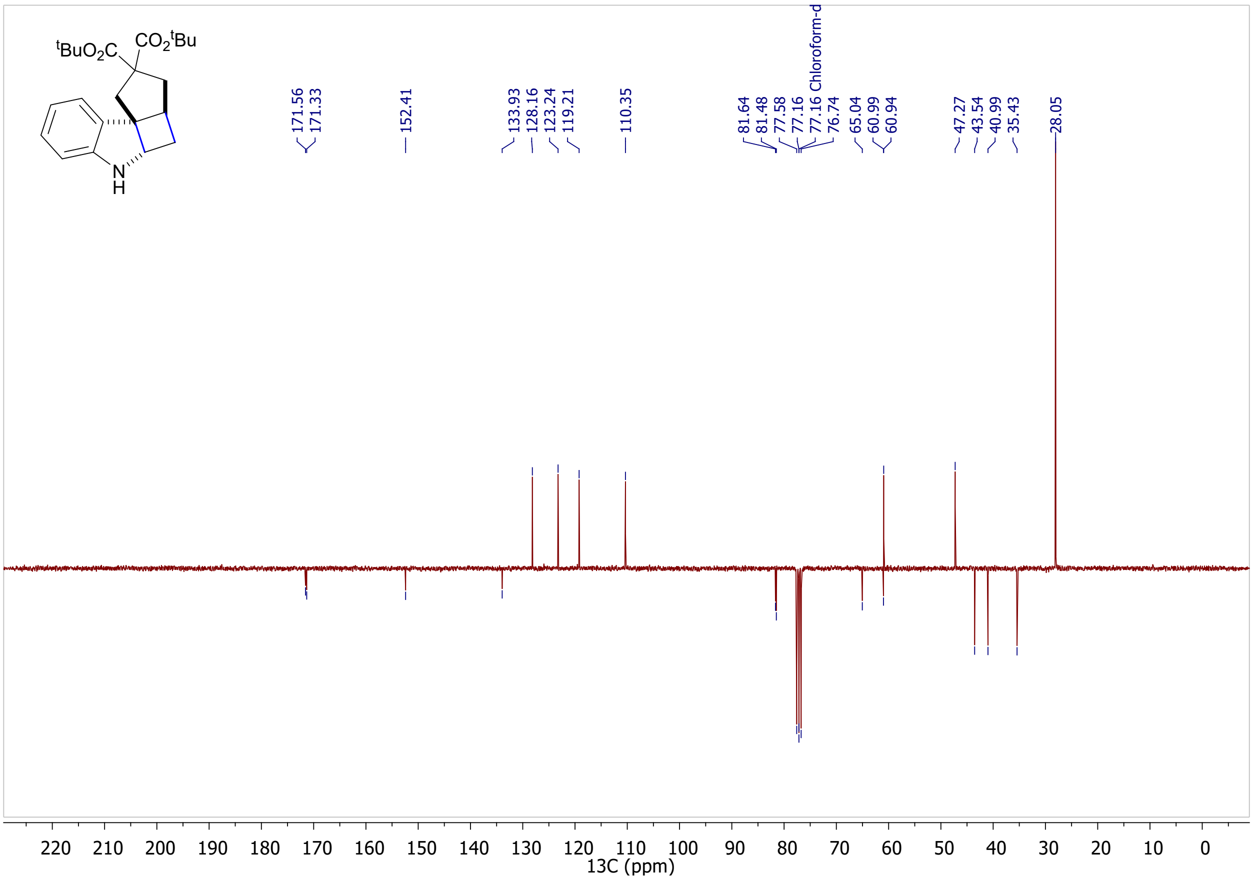

^{1}H NMR (300 MHz, $CDCl_3$) of **4e**

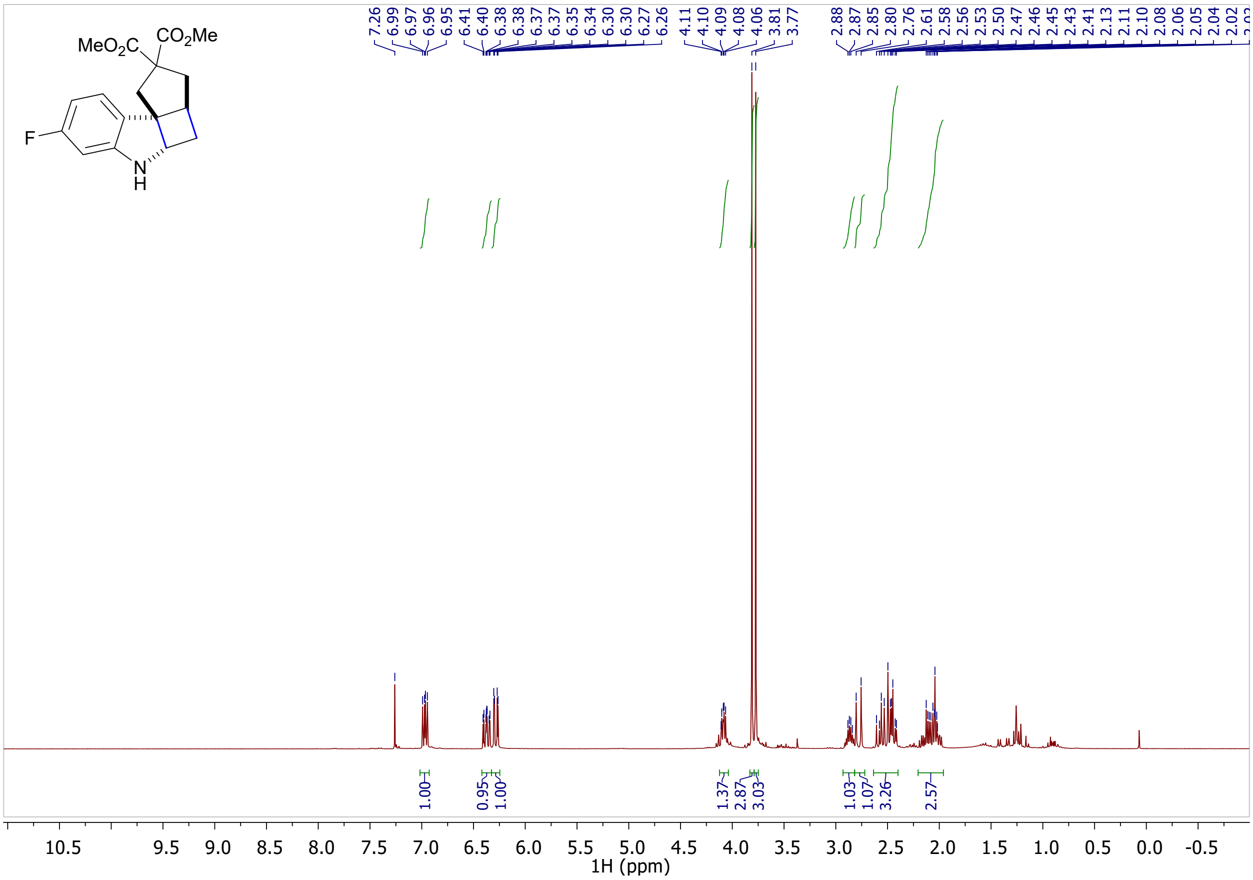

^{13}C NMR (75 MHz, $CDCl_3$) of **4e**

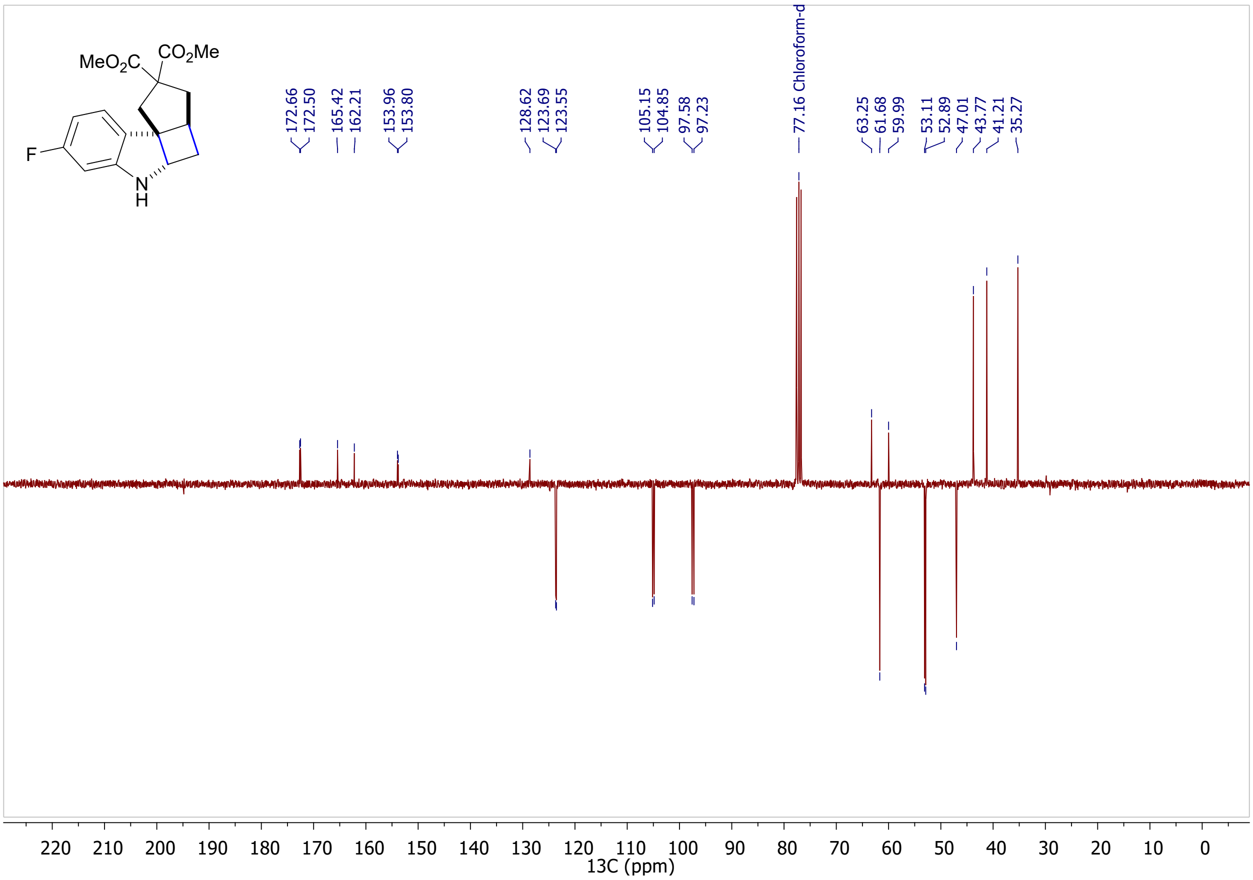

^{19}F NMR (377 MHz, $CDCl_3$) of **4e**

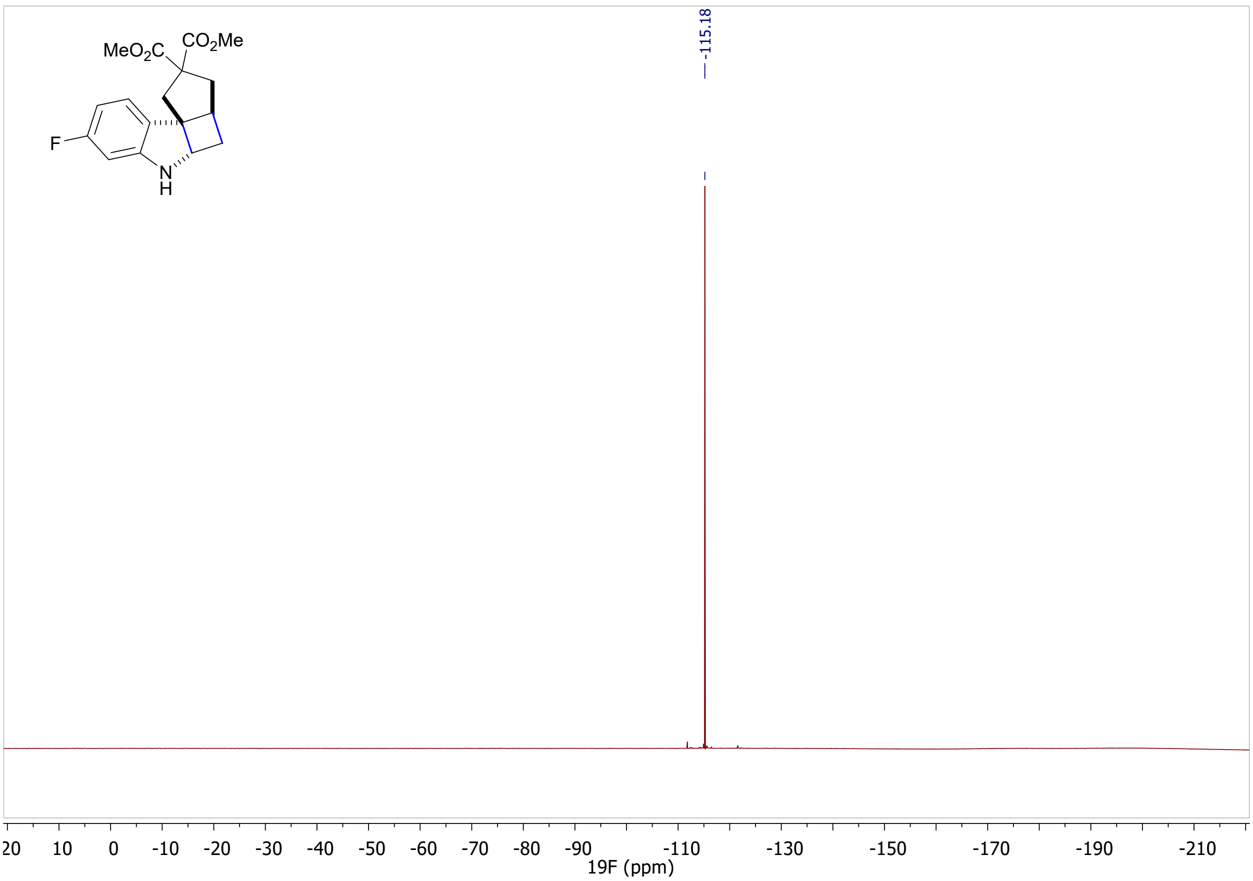

^{1}H NMR (300 MHz, $CDCl_3$) of **4f**

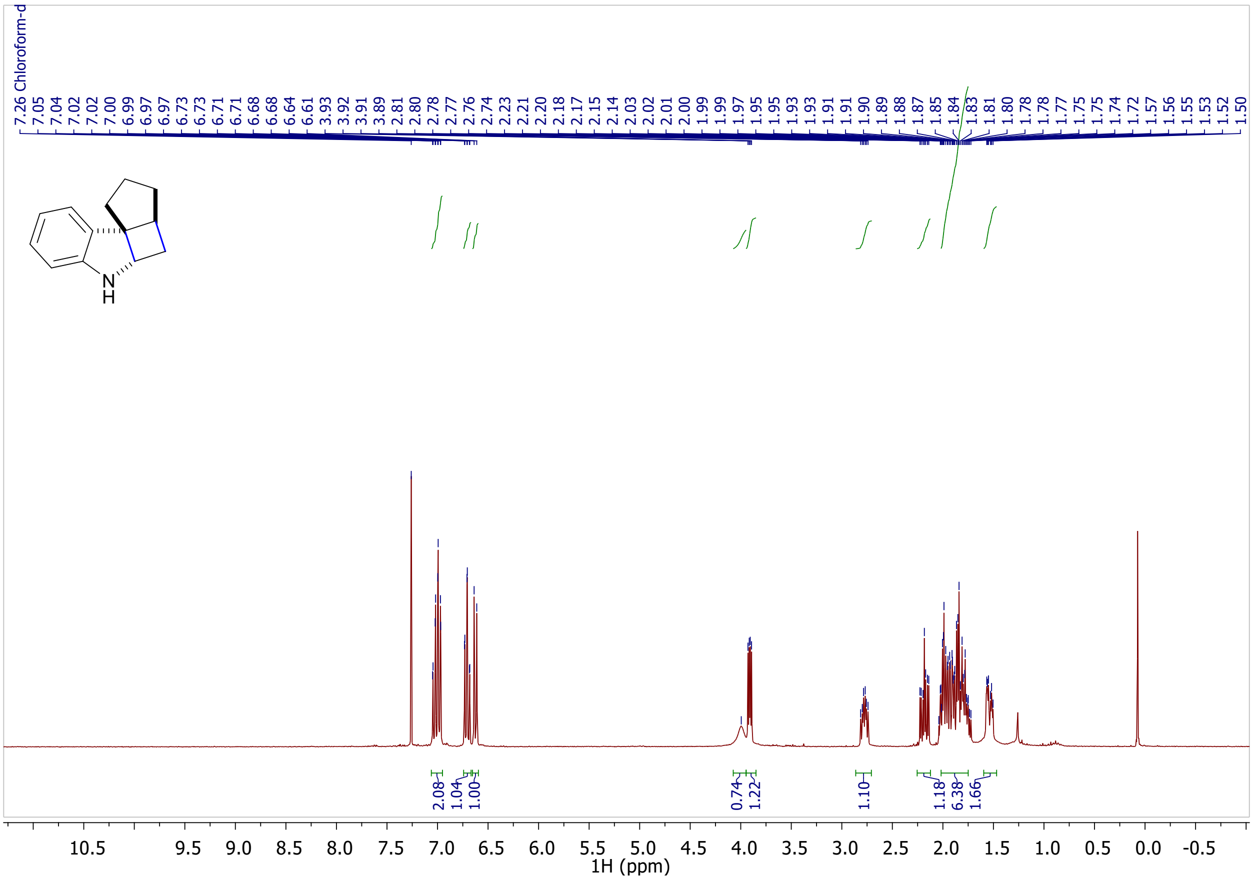

^{13}C NMR (75 MHz, $CDCl_3$) of **4f**

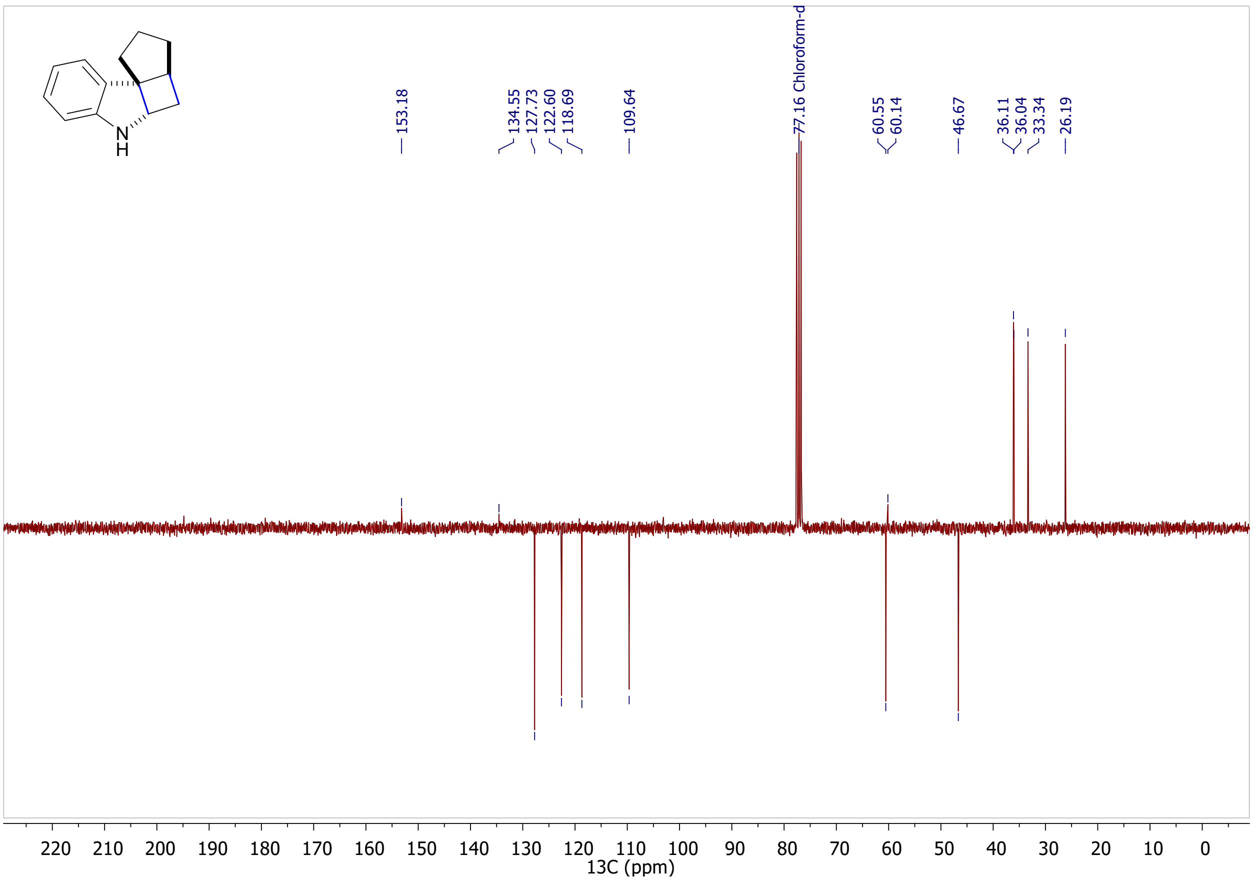

^{1}H NMR (300 MHz, $CDCl_3$) of **4g**

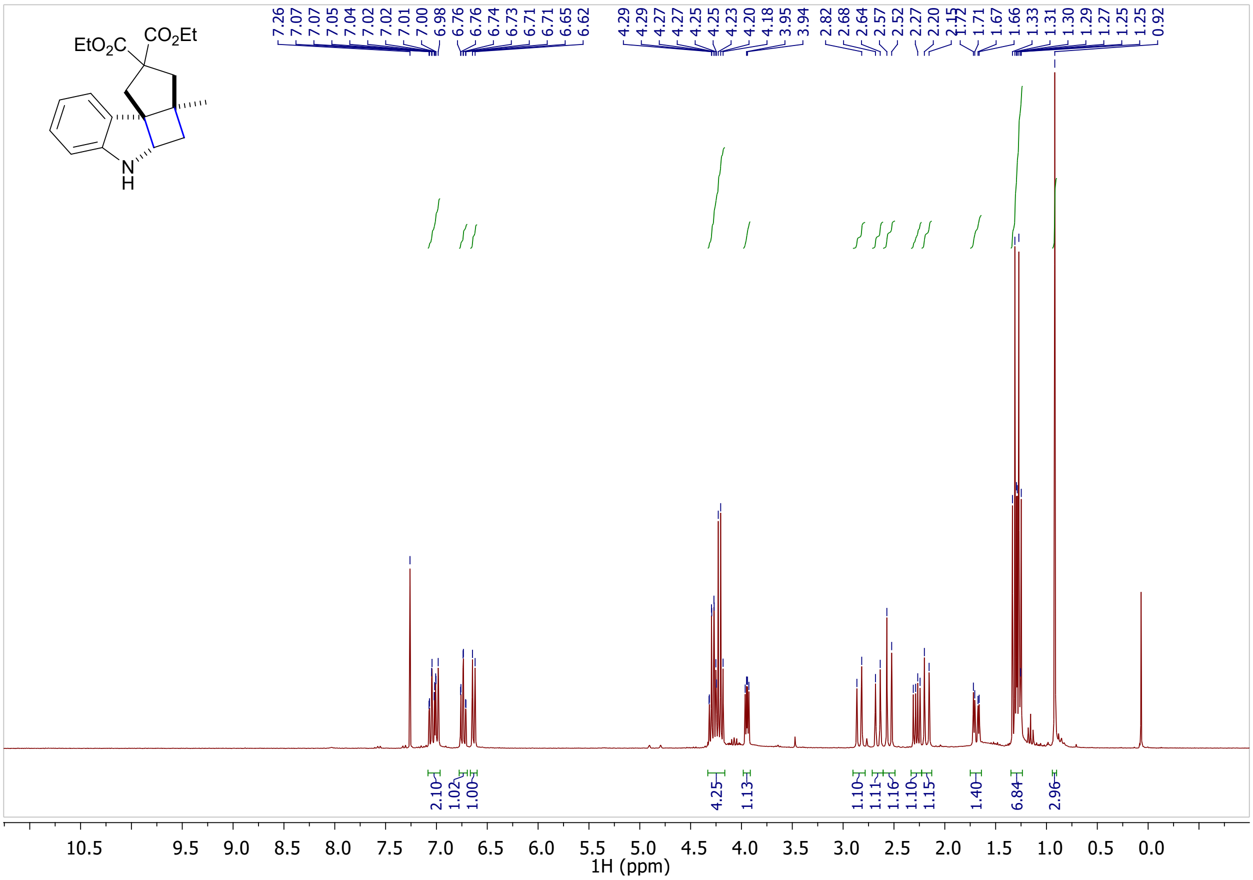

^{13}C NMR (75 MHz, $CDCl_3$) of **4g**

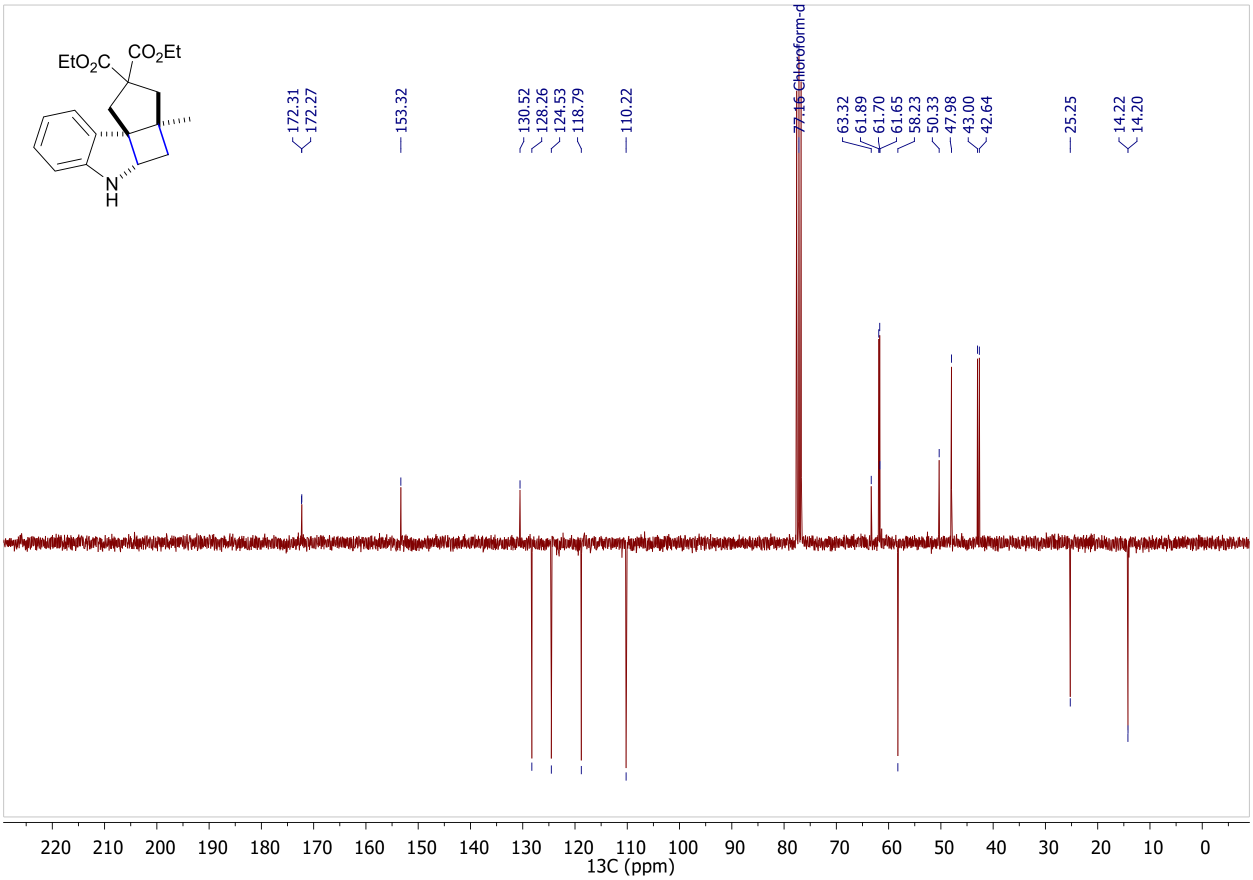

^{1}H NMR (300 MHz, $CDCl_3$) of **4k**

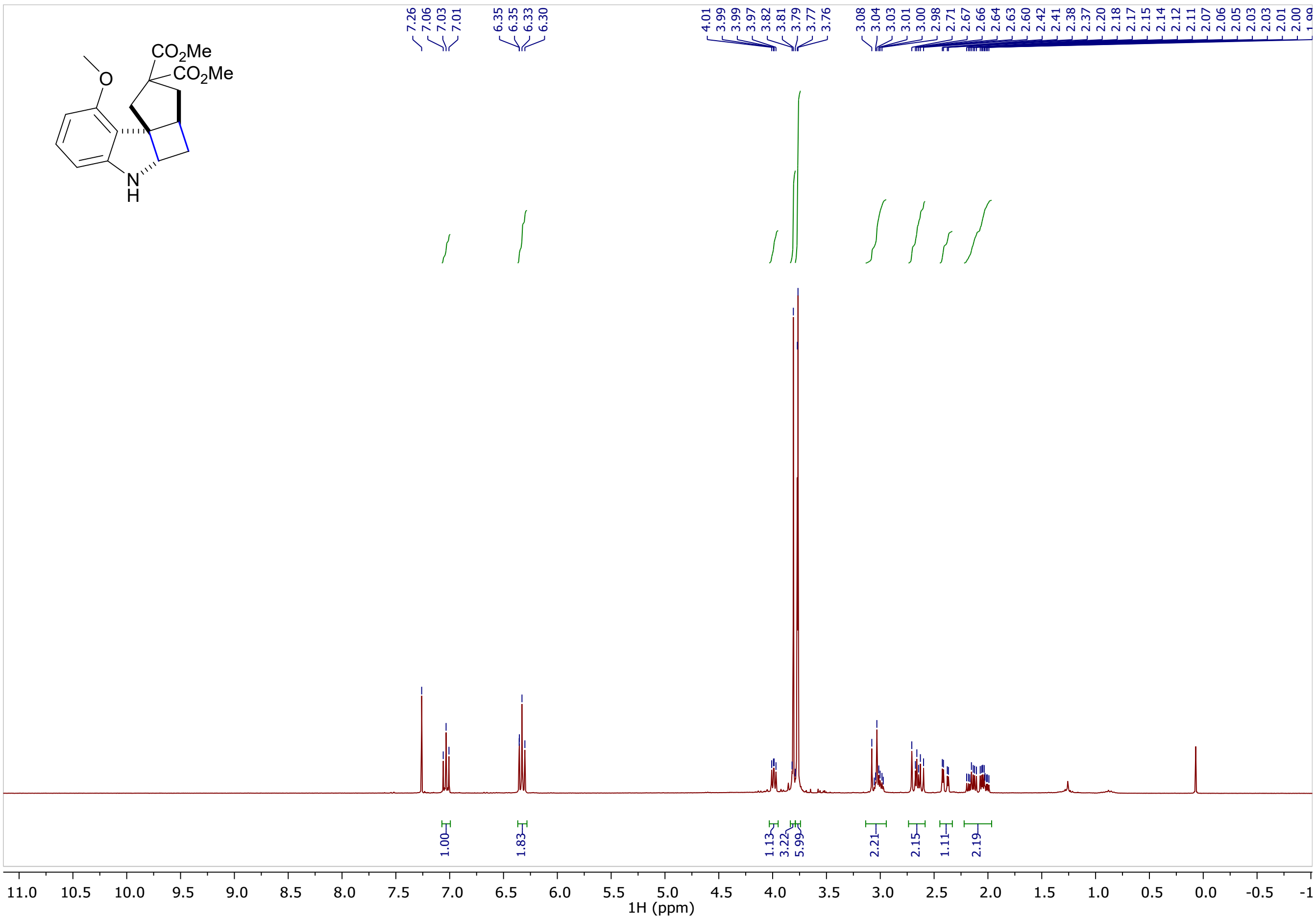

^{13}C NMR (75 MHz, $CDCl_3$) of **4k**

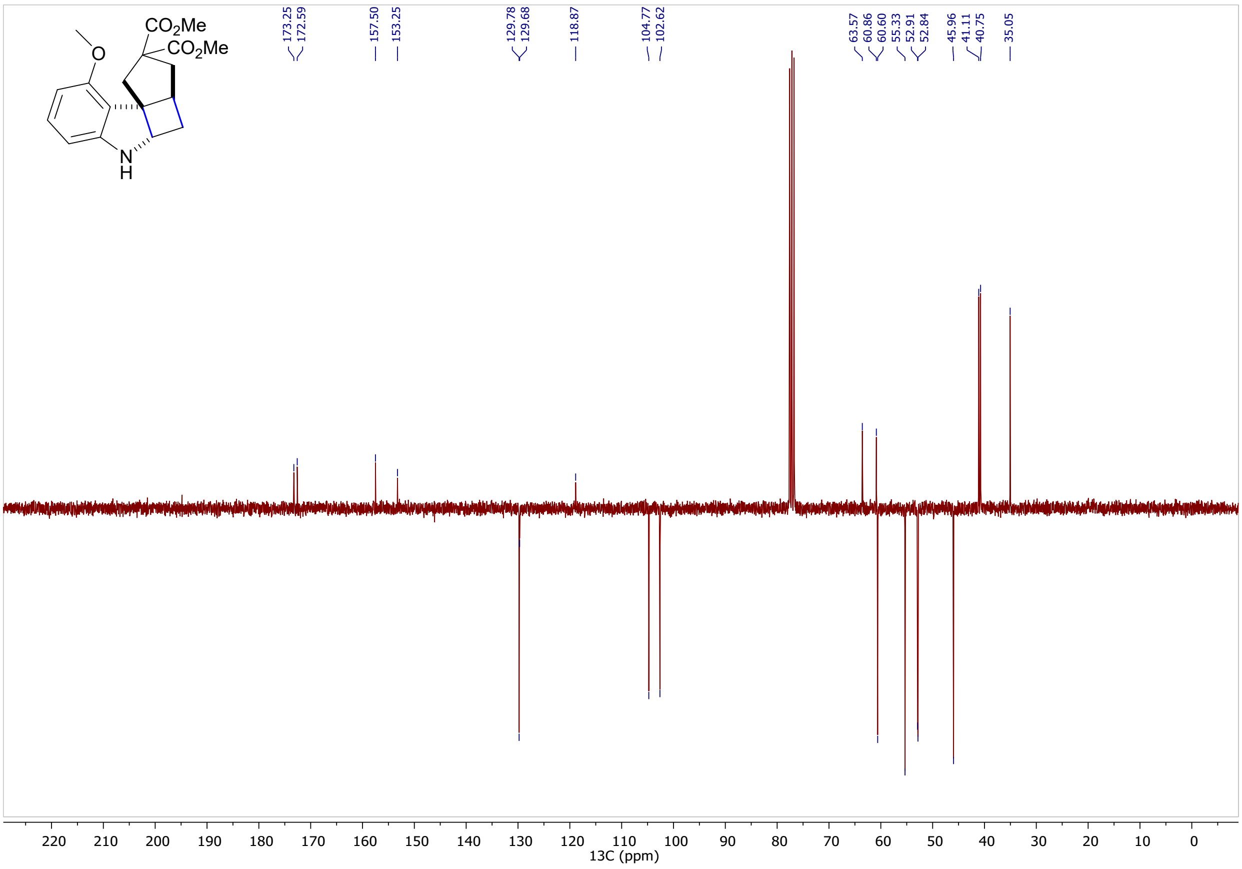

^{1}H NMR (300 MHz, $CDCl_3$) of **5g**

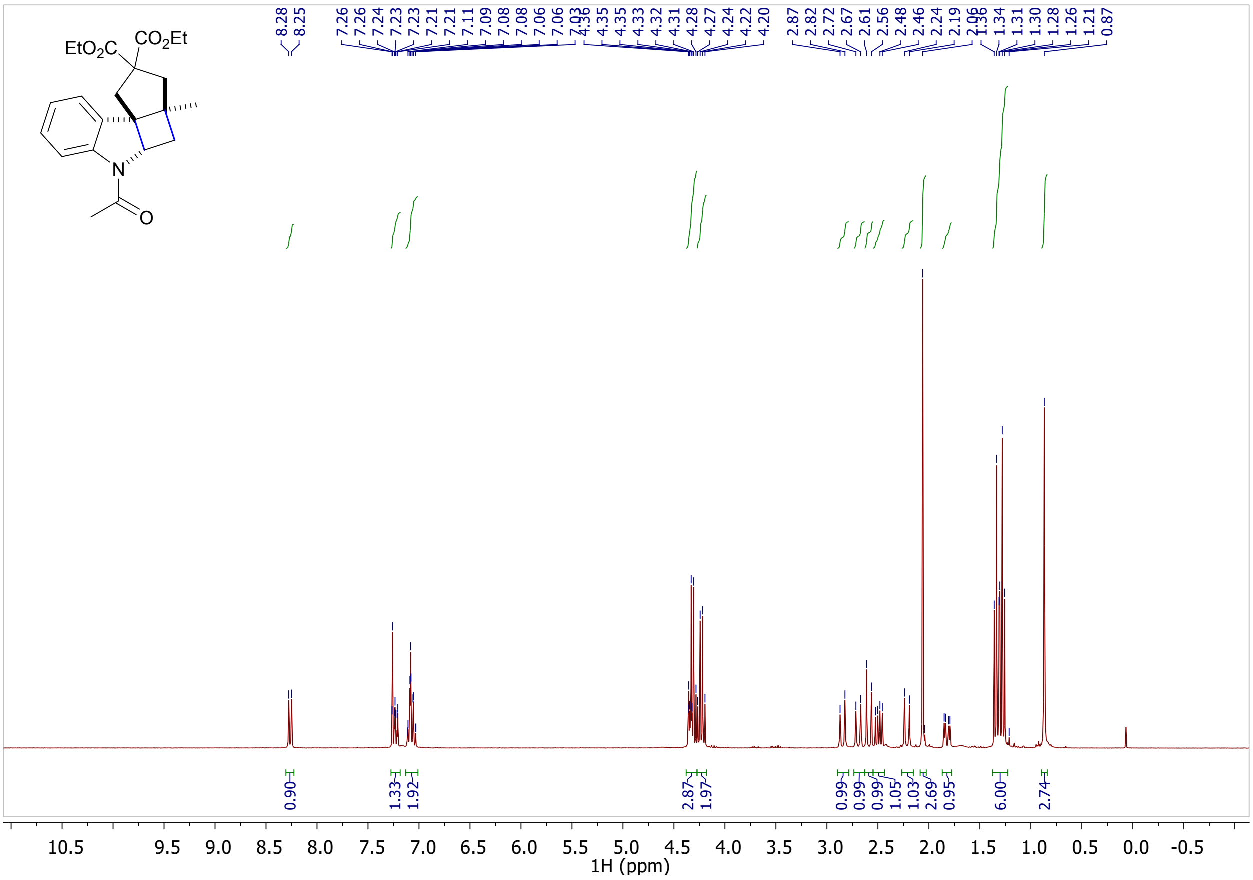

^{13}C NMR (75 MHz, $CDCl_3$) of **5g**

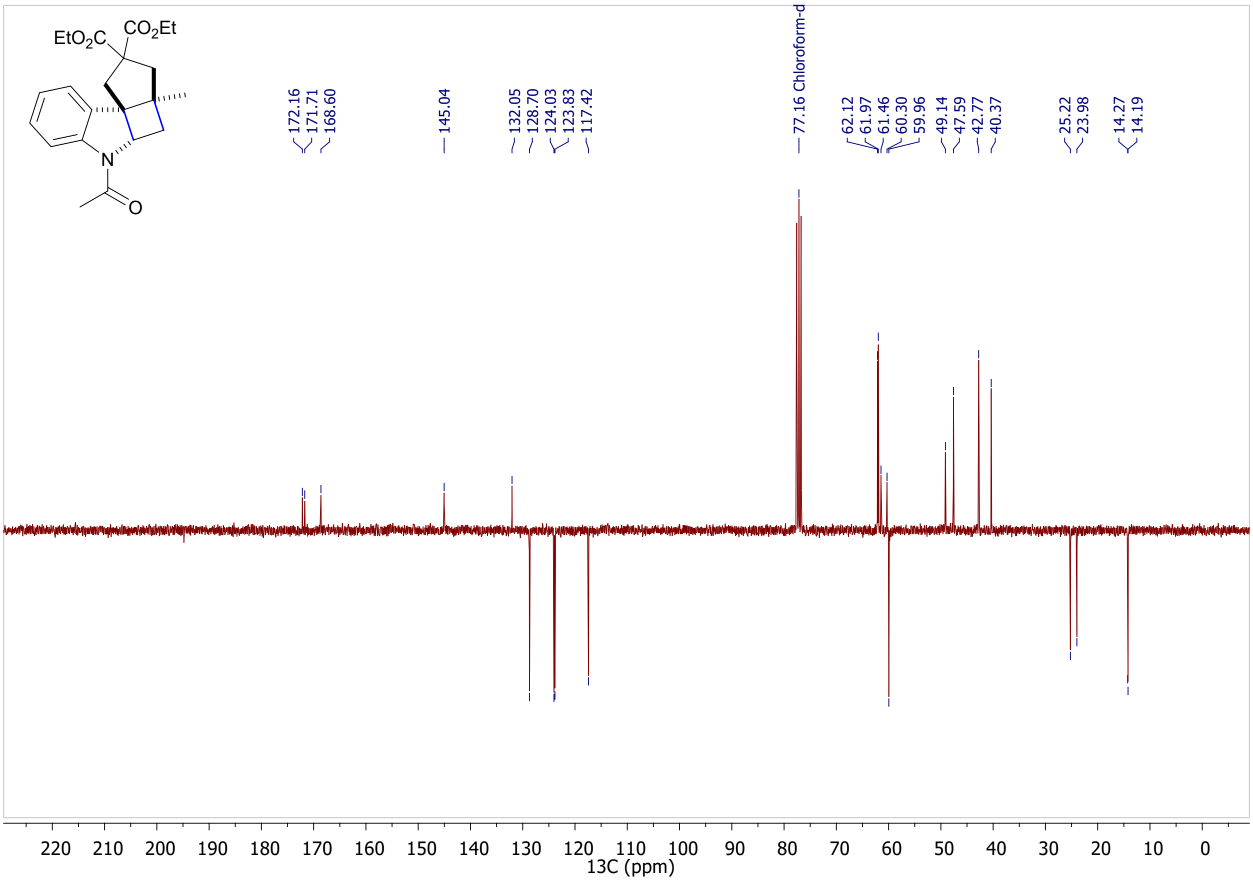

Supplement: SC-013-D2SC00864E-s001 [file SC-013-D2SC00864E-s001.pdf]
